# Perturbed iron biology in the prefrontal cortex of people with schizophrenia

**DOI:** 10.1038/s41380-023-01979-3

**Published:** 2023-02-07

**Authors:** Amit Lotan, Sandra Luza, Carlos M. Opazo, Scott Ayton, Darius J. R. Lane, Serafino Mancuso, Avril Pereira, Suresh Sundram, Cynthia Shannon Weickert, Chad Bousman, Christos Pantelis, Ian P. Everall, Ashley I. Bush

**Affiliations:** 1 Melbourne Dementia Research Centre, Florey Institute of Neuroscience and Mental Health, The University of Melbourne, Melbourne, VIC 3010 Australia; 2 Department of Psychiatry and the Biological Psychiatry Laboratory, Hadassah-Hebrew University Medical Center, Jerusalem, Israel; 3 Melbourne Neuropsychiatry Centre, Department of Psychiatry, The University of Melbourne & Melbourne Health, Carlton, VIC Australia; 4 Department of Psychiatry, School of Clinical Sciences, Monash University, Melbourne, VIC Australia; 5 Mental Health Program, Monash Health, Melbourne, VIC Australia; 6 Schizophrenia Research Laboratory, Neuroscience Research Australia, Randwick, NSW Australia; 7 School of Psychiatry, Faculty of Medicine, University of New South Wales, Sydney, NSW Australia; 8 Hotchkiss Brain Institute, Cumming School of Medicine, University of Calgary, Calgary, AB Canada; 9 Alberta Children’s Hospital Research Institute, Cumming School of Medicine, University of Calgary, Calgary, AB Canada; 10 Departments of Medical Genetics, Psychiatry, Physiology & Pharmacology, University of Calgary, Calgary, AB Canada; 11 The Cooperative Research Centre (CRC) for Mental Health, Melbourne, VIC Australia; 12 North Western Mental Health, Melbourne, VIC Australia; 13 Institute of Psychiatry, Psychology and Neuroscience, King’s College London, London, UK

**Keywords:** Neuroscience, Schizophrenia

## Abstract

Despite loss of grey matter volume and emergence of distinct cognitive deficits in young adults diagnosed with schizophrenia, current treatments for schizophrenia do not target disruptions in late maturational reshaping of the prefrontal cortex. Iron, the most abundant transition metal in the brain, is essential to brain development and function, but in excess, it can impair major neurotransmission systems and lead to lipid peroxidation, neuroinflammation and accelerated aging. However, analysis of cortical iron biology in schizophrenia has not been reported in modern literature. Using a combination of inductively coupled plasma-mass spectrometry and western blots, we quantified iron and its major-storage protein, ferritin, in post-mortem prefrontal cortex specimens obtained from three independent, well-characterised brain tissue resources. Compared to matched controls (*n* = 85), among schizophrenia cases (*n* = 86) we found elevated tissue iron, unlikely to be confounded by demographic and lifestyle variables, by duration, dose and type of antipsychotic medications used or by copper and zinc levels. We further observed a loss of physiologic age-dependent iron accumulation among people with schizophrenia, in that the iron level among cases was already high in young adulthood. Ferritin, which stores iron in a redox-inactive form, was paradoxically decreased in individuals with the disorder. Such iron-ferritin uncoupling could alter free, chemically reactive, tissue iron in key reasoning and planning areas of the young-adult schizophrenia cortex. Using a prediction model based on iron and ferritin, our data provide a pathophysiologic link between perturbed cortical iron biology and schizophrenia and indicate that achievement of optimal cortical iron homeostasis could offer a new therapeutic target.

## Introduction

Current treatments for schizophrenia do not address its most debilitating symptoms such as blunting of affect and volition and impairment in executive and social functioning [1]. Moreover, these treatments do not target the rapid loss of grey matter volume most prominent in the first few years following illness onset [2–7]. Notably, these changes coincide with the emergence of distinct cognitive deficits and disruptions in late maturational and early-adulthood reshaping of the prefrontal cortex (PFC) [7–9]. Consistent with imaging data, molecular markers are also indicative of accelerated cortical aging in schizophrenia [10], possibly contributing to a markedly increased rate of dementia in late life for people with schizophrenia [11]. Accordingly, deciphering the mechanisms through which chronic stress [12, 13], neuroinflammation [14–18], redox dysregulation [19, 20], drug abuse [21–23] and antipsychotic treatment [24, 25] could accelerate cortical tissue loss during late neurodevelopment becomes critical [8, 9]. Iron, the most abundant transition metal in the brain, has been implicated in these potential insults [26–33].

Distinct from heme-iron, most tissue iron is bound to the intracellular protein ferritin and stored in neurons and glia [34]. When required, iron in ferritin can be mobilized to serve as an essential cofactor for energy production, myelination, and catecholamine synthesis [34]. The blood-brain barrier (BBB) conducts homeostatic regulation of iron that is largely independent of peripheral stores [34]. Iron concentration varies considerably between brain regions and is typically higher in the basal ganglia [35]. Brain iron content dramatically increases during childhood and adolescence, and in the iron-rich striatum, iron levels in young-adulthood have been positively correlated with cognitive function [36]. This is consistent with iron supply being a major limiting factor for neurodevelopment and consistent with the evolution of the BBB to retain iron under conditions of malnutrition [37].

For yet-to-be-identified purposes, most brain regions continue to accumulate iron, albeit at a slower rate, throughout adult life [38, 39]. Given its redox properties, continuous iron build-up can instigate oxidative stress and neuroinflammation [40]. Beyond normal aging, accelerated iron accumulation that echoes spatial disease patterns emerged as a key pathophysiologic component of both rare monogenic disorders (collectively known as neurodegeneration with brain iron accumulation) [41] and common neurodegenerative diseases, including Alzheimer’s disease (AD), Parkinson’s disease (PD) and amyotrophic lateral sclerosis (ALS) [40, 42]. In AD, characterized by iron elevation in tangle-bearing neurons [43], post-mortem brain iron content was strongly associated with the rate of cognitive decline [44]. While neuronal degeneration is not obvious in schizophrenia, the rapid loss of PFC volume around the time of disease onset is thought to reflect a reduction in interneuronal neuropil, putatively arising from exaggerated synaptic pruning during adolescence [45]. As neuroinflammation, oxidative stress and increased dopamine stimulation of the mesocortical pathway could trigger abnormal brain maturation in early-course schizophrenia [46–50], and in light of their relationship with brain iron [26–30], we hypothesized that perturbed cortical iron biology might also contribute to the pathophysiology of schizophrenia.

In the present study, we focused on the PFC, whose dysfunction is a hallmark of schizophrenia [51]. In healthy individuals, the phylogenetically young PFC has lower iron content compared to other cortical and subcortical regions [52], and convergent post-mortem and imaging data indicate that iron accumulation in the PFC markedly slows after the third decade of life [39, 52]. As the third decade of life coincides with the modal time of first onset of schizophrenia and with the plateauing of a rapid iron accumulation period, we attempted to analyse post-mortem brain samples across a wide age range, including a subset of individuals who died in young adulthood. As accelerated structural [53] and molecular [10] aging is already evident in early schizophrenic psychosis, this young subset facilitated a neurodevelopmental-perspective assessment of how an age-by-disease interaction shapes PFC iron.

Based on specimens from three independent brain tissue resources, we show here that prefrontal iron content in individuals with schizophrenia is elevated compared to matched controls. We demonstrate that this iron elevation, which likely reflects the disorder itself rather than confounders such as demographic variables, lifestyle, or antipsychotic medications, confers the highest disease risk in individuals who died as young adults (<35). Next, we show that protein levels of ferritin, which stores iron in a redox-inactive form, are decreased in schizophrenia patients, and this decrease can predict disease more accurately in individuals with otherwise low iron levels. Finally, we show that a classification based on tissue levels of iron and ferritin and their ratio is superior to any one of these measures alone in discriminating cases from controls, providing a rationale for implicating perturbed cortical iron biology in schizophrenia.

## Methods

### Brain samples

Post-mortem human brain samples were derived from three independent brain tissue resources: New South Wales Brain Tissue Resource Center (NSW-BTRC), Victorian Brain Bank Network (VBBN) and National Institute of Mental Health Human Brain Collection Core (NIMH-HBCC). Individuals with a diagnosis of schizophrenia or schizoaffective disorder and healthy controls were matched for age, sex, brain pH and post-mortem interval (PMI). Ascertainment and exclusions are described in the Supplementary Methods, with clinical and post-mortem characteristics summarized in Tables S1, S2. All procedures were conducted in accord with principles expressed in the Declaration of Helsinki and approval was obtained from appropriate Ethics Committees.

### Tissue collection and processing

Brains were obtained at autopsy (see Supplementary Methods for details relating to specific brain banks), stored frozen (−80° C) and PFC tissue was dissected and processed as previously described [28]. Briefly, tissue was homogenized 1/5 (w/v) in 50 mM Tris-HCl pH 7.5 containing 1% NP-40 (v/v) for NSW-BTRC samples, glycerol 50% (v/v) for VBBN samples, or Tris-HCl followed by Tris-HCl and 1% NP-40 (v/v) (sequential extraction) for NIMH-HBCC samples. The homogenization buffers for NSW-BTRC and NIMH-HBCC samples also contained 10 mM DTT, 150 mM NaCl, 10 mM *N*-ethylmaleimide (NEM), Roche cOmplete™, EDTA-free Protease Inhibitor Cocktail (Cat 5056489001, Sigma-Aldrich, USA) and phosphatase inhibitors cocktail, Roche PhosSTOP (Cat 4906837001, Sigma-Aldrich, USA). The homogenization buffer for VBBN samples also contained Roche cOmplete™, EDTA-free Protease Inhibitor Cocktail (Cat 5056489001, Sigma-Aldrich, USA) and aprotinin (0.1 mg/mL). Homogenized samples were centrifuged at 10,000 *g* for 10 min at 4 °C. Supernatants were collected and protein concentrations determined using BCA Protein Assay Kit (Pierce, USA). Supernatants were aliquoted and stored at −80 °C until use.

### Metal quantification

50 μL of soluble fractions were lyophilized for 12 h and then digested with 50 μL of nitric acid (65% Suprapur, Merck) overnight (~12 h) at ~22 °C. Further digested the samples by under heating conditions (90 °C for 20 min). Samples were then removed from the heating block and an equivalent volume of 50 µL hydrogen peroxide (H_2_O_2_) (30% Aristar, BDH) was added to each sample. Samples were allowed to stop effervescing, for 30 minutes, before heating again for a further 15 minutes at 70 °C. Then cooled to ~22 °C. Samples were further diluted with 1% HNO_3_ diluent up to 500 μL (dilution factor 1:10) and iron and copper were measured by inductively coupled plasma-mass spectrometry (ICP-MS), using an Agilent Technologies 7700× ICP-MS system (Agilent Technologies, Australia) under routine multi-element operating conditions. As previously described [29], helium (3 ml/min) was used as the collision gas to minimize polyatomic interferences with all elements. The instrument was calibrated using 0, 5, 10, 50, 100 and 500 ppb of certified multi-element ICP-MS standard calibration solutions (ICP-MS-CAL2-1, ICP-MS-CAL-3 and ICP-MS-CAL-4, Accustandard) for a range of elements. Used a certified internal standard solution containing 200 ppb of Yttrium (Y89) as an internal control. The samples were analysed without identification. For each sample, iron, copper and zinc levels were corrected for protein concentration determined by BCA protein assay (Cat 23225, Thermo Fisher Scientific, USA) and expressed in μmol/g protein.

### Protein quantification

Soluble fractions (30 µg protein) of each sample were loaded in 4–12% SDS-polyacrylamide gels for 1 h at 120 V. Proteins were transferred to a PVDF membrane for 1 h at 20 V. Blots were blocked with 5% (w/v) skim milk in Tris-buffered saline containing 0.1% Tween-20 (TBST), and were then incubated overnight with the primary antibodies diluted in TBST containing 3% (w/v) BSA (Table S3) at 4 °C. After four washes with TBST, blots were incubated with IR680- and IR800-conjugated secondary antibodies (1:10,000) in TBST, 0.1% SDS for 1 h at room temperature protected from light. Blots were washed four times with TBST for 5 min each and immersed in PBS. Images were acquired in a Li-Cor imager (Li-Cor, Lincoln, USA). Using Odyssey 4.0, boxes were manually placed around each band of expected molecular weight to obtain integrated intensity values. Data were expressed as arbitrary units relative to loaded control proteins.

### Statistical analysis

Demographic and tissue-quality variables were compared across diagnostic groups using independent-samples t-tests for continuous variables or Pearson’s-chi^2^-test for categorical variables. Data from the three independent brain banks were combined using z-scores derived from the control distribution. To address the presence of outliers in our dataset, linear analyses were based on a robust regression approach using MM-estimators, which combine high resistance to outliers and high efficiency [54]. Analyses were performed with and without covariates, with propensity score matching applied when relevant. In logistic models, highly influential observations were identified using regression diagnostics [55], leading, on average, to exclusion of two samples (~1%) per analysis. Statistics were carried out in Stata 16 (StataCorp LLC, USA) and visualized with Prism 9 (GraphPad Software, USA). Further details depicting the statistical tools used at each step of the analysis are available in the Supplementary Methods.

## Results

**Fig. 1:**
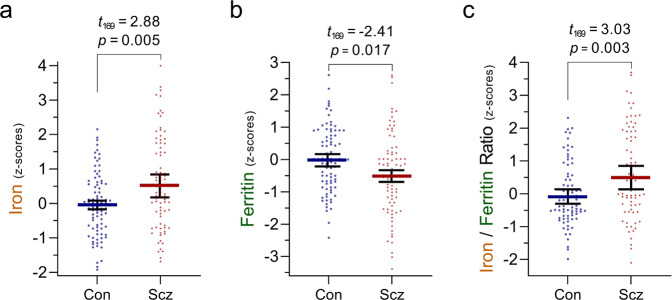
Prefrontal levels of iron, ferritin, and iron-to-ferritin ratio in schizophrenia. Comparing prefrontal levels of iron, ferritin, and iron-to-ferritin ratio across diagnostic groups. Scatter plots with bars depicting means (±95%CI) of prefrontal cortex **a** iron, **b** ferritin and **c** iron-to-ferritin ratio, presented as z-scores, among control individuals and schizophrenia cases. Values above bars represent *t*-statistics and corresponding significance of diagnosis effect. Based on robust (IRWLS MM-estimators (regression models). z-scores were derived using controls’ distribution). n_controls_ = 85, n_schizophrenia_ = 86.

In a preliminary analysis of NSW-BTRC samples, mean PFC iron among schizophrenia cases (16.4 [95%CI 14.6 to 18.2] µmol/g, n = 38) was higher by 29% (95%CI 11% to 47%) compared to matched controls (12.7 [95%CI 11.2 to 14.2] µmol/g, *n* = 37, Figure S1a). Following replication in an independent sample set from VBBN and NIMH-HBCC (n = 48/clinical group, mean group difference = 2.15 [95%CI 0.07 to 4.22] µmol/g, Figure S1b), we pooled all samples together. Since analytical samples from the three cohorts were prepared by slightly different extraction protocols that could impact on iron levels, we normalized each cohort by its mean iron level, generating z-scores for each individual sample. In the combined cohort (n_control_ = 85, n_scz_ = 86, Table 1), iron distributions satisfied robust normality assumptions (Figure S2a, Table S4), yet there were some individuals with high values (i.e > 4-5x SD), which led us to use a robust linear regression approach to evaluate mean differences. Iron in schizophrenia cases was higher by 0.58 (95%CI 0.18 to 0.97) standard deviations (SDs) compared with controls (Fig. 1a and S2b, Table S5).

**Table 1:** Clinical and postmortem characteristics of the Combined Brain Cohort.

		Control		Schizophrenia		*p*-value^a^
		*N*	Fraction	*N*	Fraction	
Number of samples		85		86		
Brain Bank	NSW-BTRC VBBN NIMH-HBCC	37 18 30	0.44 0.21 0.35	38 19 29	0.44 0.22 0.34	
Sex	Female Male	24 61	0.28 0.72	27 59	0.31 0.69	0.652
Ancestry	Caucasian Non-Caucasian	69 16	0.81 0.19	70 16	0.81 0.19	0.971
BMI^b^, kg/m^2^ (mean ± s.d.)		29.3 ± 6.1		27.9 ± 6.2		0.376
Smokers^b,c^	No Yes	37 18	0.67 0.33	15 43	0.26 0.74	**<0.001**
Alcohol users^b,d^	No Yes	18 5	0.78 0.22	17 15	0.53 0.47	0.058
Death circumstances^e^	Natural Non-natural	79 6	0.93 0.07	55 31	0.64 0.36	**<0.001**
Age of death, years (mean ± s.d.) [range]		54.4 ± 14.9 [17–85]		52.6 ± 16.2 [17–84]		0.441
pH (mean ± s.d.)		6.54 ± 0.29		6.49 ± 0.28		0.187
PMI, hours (mean ± s.d.)		33.1 ± 15.3		36.5 ± 18.4		0.189

^a^Significance of between-group comparison (control vs. schizophrenia) based on independent-samples t-test (continuous variables) or Pearson-chi^2^ (categorical variables).

^b^Data for BMI, smoking and alcohol use were available for a sizeable subset of individuals (Table S1).

^c^Smokers were defined as current smokers, heavy ex-smokers or individuals with a positive postmortem toxicology essay for nicotine.

^d^Alcohol users were defined as those with a history of drinking an average of ≥20 g ethanol/day.

^e^As no suicide cases have been documented in the control group, for ‘death circumstances’ to be a meaningful covariate, deaths by accidents and suicides were pooled together into a ‘Non-natural’ category.

*NSW-BTRC* New South Wales Brain Tissue Resource Centre, *VBBN* Victoria Brain Bank Network, *NIMH-HBCC* National Institute for Mental Health Human Brain Collection Core, *BMI* body mass index, *PMI* post-mortem interval.

Following the notion that mean and variance measures provide complementary insights, examining within-group heterogeneity is increasingly emphasized in schizophrenia research [56]. We identified markedly increased PFC iron heterogeneity in schizophrenia patients, manifested as a wider distribution (Figure S3a), with a variance more than double of that in controls (Figure S3b, Table S6). Reflecting clinical heterogeneity, gene-environment interactions, secondary disease factors, or any combination of the above, this finding is consistent with recent reports indicating that relative to healthy controls, people with schizophrenia display increased interindividual differences in cortical structure [57, 58] and function [56].

Controlling for diagnosis, neither demographic (age and mode of death, sex, and ethnicity) nor sample-quality (pH and PMI) variables were associated with PFC iron (Table S7). The effect of diagnosis on iron displayed only minimal changes when controlling for these covariates individually or simultaneously (Table S7) or following regression-adjusted propensity-score matching (Tables S8, S9 and Figures S4, S5), consistent with the effect on iron reflecting the disease itself. For sizeable subsets of individuals (one to two thirds of entire cohort), data relating to smoking habits, alcohol use and body-mass-index (BMI) were available (Table S1). As expected [59, 60], smoking and alcohol use were more prevalent among schizophrenia cases (Table 1), yet consistent with a previous report [38], these habits were not associated with PFC iron, and when included as covariates, the effect of diagnosis remained significant (Tables S10, S11). BMI did not differ between the matched groups analysed here (Table 1), and iron discriminated cases from controls when controlling for BMI (Table S12).

Based on limited animal data indicating that chronic treatment with typical neuroleptics altered the BBB and facilitated brain iron uptake [32, 33], a small iron elevation previously noted among schizophrenia patients was intuitively ascribed to neuroleptic treatment, rather than to the pathophysiology of the disease itself [61]. While a causal link between antipsychotics and iron elevation cannot be unequivocally addressed in postmortem tissue, based on data available for a subset of cases in our cohort (*n* = 43, Table S2), PFC iron levels were not correlated with mean antipsychotic daily dose or cumulative lifetime exposure (Figure S6b, Table S13). These results remained similar after adjusting for age-of-onset and treatment duration (Table S14). Moreover, while animal data indicated that atypical neuroleptics could have less profound effects on brain iron compared to typical agents [32], we did not find an effect of neuroleptic (a)typicality on PFC iron levels (Table S15). Consistent with a previous report that indicated no correlation between a neuroleptic-free period before death and brain iron [62], we did not find an association between presence of antipsychotic drugs (post-mortem toxicology assay) and PFC iron (Table S16). Finally, while lithium pharmacotherapy was recently reported to increase iron levels in the substantia nigra and hippocampus of teenagers who were at high risk of psychosis [63], the paucity of patients in our sample with positive lithium toxicology (*n* = 4) rendered this drug an unlikely confounder. Taken together, our data are consistent with the notion that in schizophrenia, PFC iron elevation represents a primary disease-related, rather than a secondary drug-induced, abnormality.

Like iron, copper is a redox-active transition metal with myriad roles in brain function, including monoamine metabolism, mitochondrial activity, and myelination [64]. Reduced copper activity results in schizophrenia-like behavioral impairments [65], yet copper dyshomeostasis in schizophrenia brain tissue has also rarely been studied. PFC copper, quantified in nearly 80% of our samples (Table S1), was normally distributed (Table S17). In contrast to a recent study reporting copper deficiency in midbrain specimens from schizophrenia cases [66], in the PFC we observed no such difference (Figure S7a, Tables S18, S19). Moreover, brain iron is thought to be modulated by ceruloplasmin, a multi-copper ferroxidase [67], and consistent with correlational data in rodents [68], a positive effect of copper on iron levels was evident across both control individuals and schizophrenia cases (Figure S7b, Table S20). As iron elevation in the schizophrenia PFC remained significant throughout the physiological range of copper levels (Figure S7b), our data do not demonstrate an overt copper perturbation in schizophrenia PFC and are consistent with a primary disturbance of iron in this disorder.

The divalent cation zinc is the second most abundant metal in the human body and is indispensable for life. Like iron, zinc concentrations must be tightly regulated as deficiencies are associated with multiple pathological conditions while an excess can be toxic [69]. Although a peripheral disturbance of zinc homeostasis in individuals with schizophrenia was reported [70], zinc content in schizophrenia brain tissue has rarely been studied. Normally distributed (Table S21), PFC zinc was marginally higher among patients (0.28 [95%CI 0.03 to 0.54] SDs, Figure S8a, Table S22), possibly reflecting elevated mRNA levels of zinc transporter SLC39A12 [71]. Unlike iron, zinc distribution among patients and controls was similar (Table S23). Likely reflecting a brain iron-zinc interdependence [68, 72], we observed a positive association between iron and zinc (Table S24, Figure S8b). However, structural equation modelling indicated that zinc was unlikely to mediate the effect of iron on diagnosis (Figure S9, Table S25), consistent with a primary disturbance of iron in schizophrenia.

To appreciate the relevance of iron perturbation in schizophrenia, we decided to quantify ferritin. Highly conserved across the animal kingdom, it forms a ~480 kDa cage complex consisting of 24 subunits (~20 kDa each) of ferritin heavy chain 1 (FTH1) and ferritin light chain (FTL), which incorporates up to several thousand Fe2^+^ ions, oxidizes them to Fe3^+^, and stores them as mineral oxides in its cavity [73]. In grey matter, ferritin is expressed in all cells to store and detoxify labile cytoplasmic Fe^2+^, rendering it chemically inactive and redox-insensitive [40]. Quantified by western blots (Figure S10a, antibody recognizes both heavy and light chains), PFC ferritin normally distributed across individuals (Table S26). We observed lower ferritin in tissue from schizophrenia cases (−0.45 [95%CI −0.82 to −0.08] SDs, Fig. 1b, Figure S10b, Table S27). This difference remained prominent following adjustment for relevant covariates (Table S28). As an elevated iron-to-ferritin ratio has been associated with iron toxicity and accelerated aging [74], we quantified individual iron-to-ferritin ratios in our sample, and noted that the magnitude by which this ratio was elevated among schizophrenia cases (0.62 [95%CI 0.22 to 1.02] SDs, Fig. 1c, Figure S10c, Table S29) was larger than the group-differences observed for iron and ferritin alone.

**Fig. 2:**
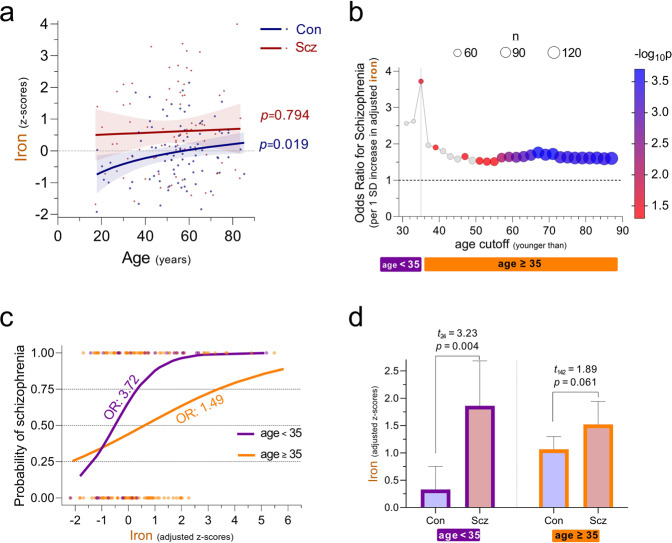
Association of prefrontal iron with age and schizophrenia. **a** Relationship between age and iron in control individuals and schizophrenia cases. Individual data points depicting prefrontal iron (*y*-axis, z-scores) against age of death (*x*-axis, years) are presented alongside model-based predictions (±95%CI) for each diagnostic group. Among control individuals, a linear-logarithmic model was superior at fitting the data. Based on robust (IRWLS MM-estimators) regression models. z-scores were derived using controls’ distribution. n_controls_ = 85, n_schizophrenia_ = 86. **b** Odds ratio of a schizophrenia diagnosis per unit iron increase, according to age of death. Based on serial logistic regression analyses, the odds ratio of having been diagnosed with schizophrenia (as compared to being a control) attributed to a 1 SD increase in covariate (sex, ethnicity, sample pH and post-mortem interval)-adjusted iron (*y*-axis) is plotted against age cutoff (*x*-axis, years). Using two-year steps beginning at age 31, each analysis included only individuals younger than the designated age cutoff. For each analysis, symbol size represents number of individuals included (n, upper-center legend), and symbol colour represents significance of iron in predicting diagnosis (-log_10_-p-value, right-hand legend, non-significant analyses were colour-coded grey). As the odds ratio was maximal when the analysis included only individuals younger than 35, this age was selected as a cutoff for generating subcohorts (visualised by purple and orange bars) to be contrasted in subsequent panels. *N* = 170. **c** Predicted probability of a schizophrenia diagnosis according to prefrontal iron levels. Using logistic regression models, predicted probabilities of having been diagnosed with schizophrenia (*y*-axis) across a continuum of prefrontal iron (*x*-axis, z-scores adjusted for covariates as above) among the subcohorts of individuals who had died younger than or older than 35 years are depicted by the purple and orange regression lines, respectively. The odds ratio of having been diagnosed with schizophrenia per unit iron increase (i.e., slope of log-transformed regression lines) is depicted for each subcohort. Circles, which represent individual iron values in the control and schizophrenia groups (assigned probabilities of 0 and 1, respectively), are coloured according to subcohort. n_age<35_ = 26, n_age≥35_ = 144. **d** Iron by diagnosis stratified by age subcohort. Bar graphs depict means (±95%CI) of prefrontal iron, adjusted for covariates as above, presented as z-scores derived from controls’ distribution and mean-centred at 1. Values above bars represent significance of diagnosis effect, based on robust (IRWLS MM-estimators) regression models. n_age<35/con_ = 9, n_age<35/Scz_ = 17; n_age≥35/con_ = 75, n_age≥35/Scz_ = 69.

In PFC tissues from control individuals, increasing age of death was associated with higher iron levels. As the rate of iron accumulation decreased with age, this relationship was best estimated using a linear-logarithmic model (*t*_83_ = 2.39, *p* = 0.019, Fig. 2a and S11a, Table S30). This observation is consistent with findings from postmortem [52] and imaging studies [38, 39, 75–77], in which the rate of age-dependent iron accumulation in the PFC slowed after the third decade of life. Notably, among schizophrenia cases PFC iron remained stable across age (*t*_84_ = 0.26, *p* = 0.794, Fig. 2a, Figure S11a, Table S31).

To further probe how differential trajectories of age-dependent iron accumulation affect schizophrenia risk, we performed a series of logistic regression analyses (Figure S11b). Using sequential age cutoffs, we examined the odds ratio (OR) of a schizophrenia diagnosis attributed to a 1 SD increase in covariate-adjusted iron (Table S32). As the OR was maximal when the analysis included only individuals younger than 35 (Fig. 2b), this age was selected as a cutoff for generating younger (age<35) and older (age≥35) subcohorts. While in both cohorts the probability of having been diagnosed with schizophrenia increased as PFC iron rose (Fig. 2c, Table S33), the effect of postmortem iron in predicting whether an individual had been previously diagnosed with schizophrenia was larger among individuals younger than 35 (OR = 3.72 [95%CI 1.16 to 11.9]) compared to the older individuals (OR = 1.49 [95%CI 1.14 to 1.95]). Mirroring the prediction models, between-group comparison revealed that the difference in covariate-adjusted iron between cases and controls was very large in the young subcohort (1.53 [95%CI 0.55 to 2.51] SDs), yet marginal in the older subcohort (0.46 [95%CI -0.02 to 0.94] SDs, Fig. 2d, Table S34). Taken together, our data are consistent with an accelerated iron accumulation in the developing schizophrenia prefrontal cortex, culminating in a pathological iron elevation whose peak occurs during the typical age of schizophrenia onset [78].

**Fig. 3:**
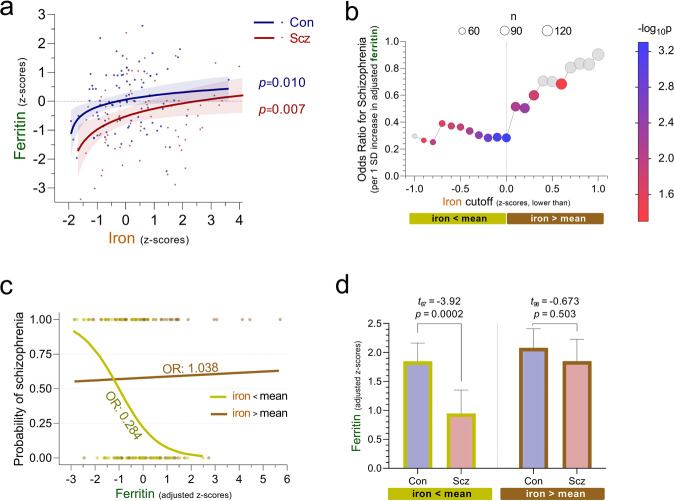
Association of prefrontal ferritin with iron and schizophrenia. **a** Relationship between iron and ferritin in control individuals and schizophrenia cases. Individual data points depicting prefrontal ferritin (*y*-axis, z-scores) against prefrontal iron (*x*-axis, z-scores) are presented alongside model-based predictions (±95%CI) for each diagnostic group. Among both groups, linear-logarithmic models were superior at fitting the data. Significance values were derived using robust (IRWLS MM-estimators) regression models. z-scores were derived using controls’ distribution. n_controls_ = 85, n_schizophrenia_ = 86. **b** Odds ratio of having schizophrenia per unit ferritin increase, according to iron status. Based on serial logistic regression analyses, the odds ratio of having a diagnosis of schizophrenia (as compared to being a control) attributed to a 1 SD increase in covariate (age, sex, ethnicity, sample pH and post-mortem interval)-adjusted ferritin (*y*-axis) is plotted against iron cutoff (*x*-axis, z-scores). Using 0.1 SD steps beginning at −1 SD, each analysis included only individuals with cortical iron levels lower than the designated cutoff. For each analysis, symbol size represents number of individuals included (n, upper-center legend), and symbol colour represents significance of cortical ferritin levels in predicting diagnosis (-log_10_-*p*-value, right-hand legend, non-significant analyses were colour-coded grey). As ferritin’s significance as a predictor was maximal when the analysis included only individuals with iron below control-group mean, the value 0 was selected as a cutoff for generating subcohorts (visualised by olive-green and brown bars) to be contrasted in subsequent panels. *N* = 169. **c** Predicted probabilities of a schizophrenia diagnosis according to ferritin. Using logistic regression models, predicted probabilities of having been diagnosed with schizophrenia (*y*-axis) across a continuum of prefrontal ferritin (*x*-axis, z-scores adjusted for covariates as above) among the subcohorts of individuals with cortical iron levels below or above mean are depicted by the olive-green and brown regression lines, respectively. The odds ratio of having been diagnosed with schizophrenia per unit ferritin increase (i.e., slope of log-transformed regression lines) is depicted for each subcohort. Circles, which represent individual ferritin values in the control and schizophrenia groups (assigned probabilities of 0 and 1, respectively), are coloured according to subcohort. n_iron<mean_ = 69, n_iron>mean_ = 100. **d** Ferritin by diagnosis stratified by iron subcohort. Bar graphs depict means (±95%CI) of prefrontal ferritin, adjusted for covariates as above, presented as z-scores derived from controls’ distribution and mean-centred at 2. Values above bars represent significance of diagnosis effect, based on robust (IRWLS MM-estimators) regression models. n_iron<mean/con_ = 43, n_iron<mean/Scz_ = 26; n_iron>mean/con_ = 42, n_iron>mean/ Scz_ = 58.

Consistent iron driving ferritin expression [79], among both control individuals and schizophrenia cases higher levels of PFC iron were mirrored by higher ferritin (*t*_83_ = 2.65, *p* = 0.010 and *t*_84_ = 2.74, *p* = 0.007, respectively, Fig. 3a and S12a). Likely reflecting the capacity of this storage protein to continue incorporating iron at higher iron-to-ferritin ratios [80], in both groups we observed a decline in the rate of ferritin accumulation upon increasing iron levels, best estimated using linear-logarithmic models (Tables S35, S36). At iron corresponding to the control group’s mean, our model predicted that ferritin among cases would be lower by 0.79 (95% CI 0.31 to 1.27) SDs (Table S37).

To probe if differential patterns of iron-dependent ferritin accumulation affected disease risk, we performed a series of logistic regression analyses using sequential iron cutoffs, each examining the OR of a schizophrenia diagnosis attributed to a 1 SD increase in covariate-adjusted ferritin (Figure S12b, Table S38). As the OR was farthest from 1 when the analysis included only individuals with iron levels below the control group’s mean (Fig. 3b), this value was selected as a cutoff for generating low-iron and high-iron subcohorts. While in the low-iron cohort the probability of having been diagnosed with schizophrenia markedly decreased as PFC ferritin rose (OR = 0.284 [95%CI 0.139 to 0.578]), in the high-iron subcohort ferritin did not predict an individual’s disease status (Fig. 3c, Table S39). Mirroring the prediction models, the difference in covariate-adjusted ferritin between cases and controls was large in the low-iron subcohort (−0.96 [95%CI −1.46 to −0.47] SDs), yet minimal (-0.17 [95%CI ‒0.67 to 0.33] SDs) in the high-iron subcohort (Fig. 3d, Table S40). Extending our previous findings indicating that elevated iron in young adulthood predicted disease status, these data reveal that even in the context of low prefrontal iron, a deficiency in iron’s protective storage protein markedly increased the risk for a schizophrenia diagnosis.

**Fig. 4:**
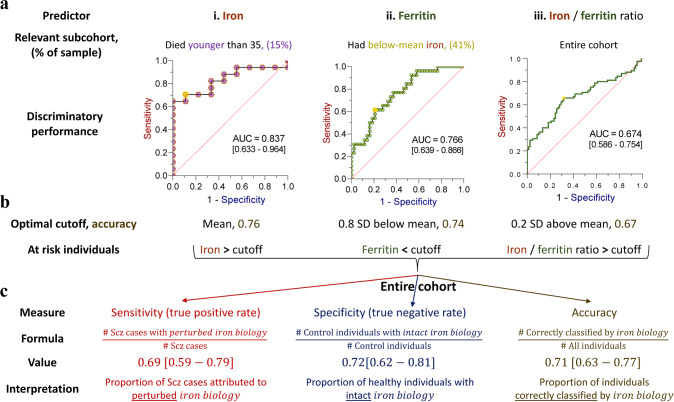
Perturbed iron biology in the prefrontal cortex discriminates schizophrenia cases from controls. **a** Discriminatory performance of covariate-adjusted (i) iron levels among the subcohort of individuals who had died younger than 35 years, (ii) ferritin levels among the subcohort of individuals with below-mean iron, and (iii) iron-to-ferritin ratio among the entire cohort, assessed using receiver operating characteristic (ROC) curve analyses. Area-under-curve (AUC) parameters [95% bias-corrected CIs] are presented. Gold circles denote optimal cutoffs. **b** Optimal cutoffs (z-score values) and their corresponding accuracy are provided for each ROC curve, alongside values that define high-risk individuals. **c** Sensitivity, specificity, and accuracy of a schizophrenia diagnosis based on an optimal cutoff derived from a logistic regression model combining all three predictors. *N* = 168.

To gauge the proportion of schizophrenia cases that could be attributed to perturbed iron biology, we quantified the discriminatory performance of iron, ferritin, and iron-to-ferritin ratio, focusing on relevant subcohorts (Figs. 2–3). As expected, while PFC iron could predict disease status in the entire cohort (Figure S13a), its discriminatory performance among individuals younger than 35 was higher (AUC = 0.837 [95%CI 0.633 to 0.964], Figure 4a_i_). Similarly, although ferritin only marginally predicted disease status in the entire cohort (Figure S13b), its discriminatory performance among individuals with low iron improved (AUC = 0.766 [95%CI 0.639 to 0.866], Figure 4a_ii_). Although iron-to-ferritin ratio was more efficient at discriminating cases from controls when focusing on younger individuals (Figure S14), it was not superior to iron alone in this age group (Fig. 2b). However, across the entire cohort, the discrimination offered by the iron-to-ferritin ratio (AUC = 0.674 [95%CI 0.586 to 0.754], Figure 4a_iii_) was higher than that of iron or ferritin alone (Figure S13). Having focused on the discriminatory performance of iron and ferritin in specific subcohorts (Figure 4a_i, ii_), by deriving optimal cutoffs (Fig. 4b, Table S41) we could assess all three predictors simultaneously (Table S42). Such a model was superior to two-predictor models (Table S43), consistent with iron, ferritin and their ratio capturing (partially) distinct pathophysiological components. 69% of schizophrenia cases were classified as having perturbed iron biology while in 72% of healthy individuals iron biology was intact (Fig. 4c, Table S44). Overall, classification based on prefrontal iron biology was 71% (95%CI 63 to 77%) accurate in predicting schizophrenia (Fig. 4c).

## Discussion

In the current study, we found that iron levels in the PFC are elevated in schizophrenia compared to age-matched controls. Ferritin, which stores iron in a redox-inactive form, is paradoxically decreased in individuals with the disorder. Such iron-ferritin uncoupling could alter free, chemically reactive, tissue iron in key reasoning and planning areas of the schizophrenia cortex. Among schizophrenia cases, we observed a loss of age-dependent iron-accumulation that characterised control individuals, in that iron levels were already high in cortices of young adults with schizophrenia. Accordingly, the difference in iron between groups was largest among young adults, with high iron levels conferring a major risk for being diagnosed with disease in this age group. Notably, individuals with low iron could still be at risk for being diagnosed with schizophrenia if their ferritin levels were diminished. A model combining tissue iron and ferritin in selected subcohorts and iron-to-ferritin ratio in the entire cohort could discriminate cases from controls with moderately good precision, highlighting a potential pathophysiological link between perturbed cortical iron biology and schizophrenia.

A perturbance of iron in schizophrenia postmortem tissue was reported a century ago, where frontal cortex specimens from individuals with paranoid schizophrenia exhibited higher iron content compared to specimens obtained from people whose diagnosis likely corresponded to the disorganized and catatonic subtypes of schizophrenia [81]. However, subsequent postmortem reports, which focused mainly on subcortical regions, were small in sample size and mostly inconclusive [61, 62, 82]. Thus, the prominent cortical iron elevation reported here could reflect enhanced availability of high-quality postmortem brain tissue coupled with robust quantification technology.

Several quantitative MRI studies reported decreased paramagnetic iron in the basal ganglia [83], midbrain [84] and thalamus [83, 84] in early phases of psychotic disorders. A conceptual difference between these imaging studies and our findings relates to regional specificity of iron dyshomeostasis in schizophrenia. Previously thought to be local, recent data indicate that regulation of brain iron depends on a complex, activity-dependent transport between functionally associated yet anatomically distinct regions [85, 86]. It is thus possible that perturbed trafficking systems in the schizophrenia brain could lead to a redistribution of iron between cortical and subcortical structures. From a methodological perspective, the ferritin pool of iron is the principal contributor to the grey matter signal measured by quantitative susceptibility mapping (QSM) [87], and we find that although iron levels are elevated, the ferritin levels are decreased. This could alter the magnetic properties of iron leading to altered MRI signals by either R1/R2* or QSM as compared with our direct measurement of absolute tissue iron. Finally, recent imaging studies focusing on patients with chronic schizophrenia reported paramagnetic iron elevation in the thalamus [88] and putamen [89], highlighting the relevance of disease phase in this context.

The cortical iron elevation we observed was unlikely to be driven by systemic iron overload. By decoupling iron levels in the periphery from the brain, the BBB renders brain iron homeostasis mostly independent of systemic iron [40], so that changes in the brain compartment do not necessarily echo systemic changes and vice versa. In fact, an inverse iron status, with lower circulating iron coupled with iron increases localised to disease-specific brain regions, was described in AD and PD [90–92]. Such recompartmentalization of peripheral and central iron could reflect decreased loading of iron into transferrin in the plasma [93], coupled with region-specific up-regulation of cellular iron import [94], accelerated breakdown of heme released from damaged mitochondria [95], accumulation of iron-rich protein aggregates [96–98], and down-regulation of cellular iron export [99–101]. As peripheral iron is often decreased in schizophrenia [102], our data are consistent with iron recompartmentalization manifesting also in this disorder.

Adequate iron nutriture is essential for motor, cognitive and social development [103]. However, emerging data indicate that the effects of peripheral iron on neurodevelopment are better described as an inverted ‘U’ [104–106]. In weanling rats, neurobehavioral dysfunctions associated with dietary iron overload were similar to those observed in iron-deficient animals [107], and a review of preclinical data identified a consistent adverse effect of high neonatal iron intake on brain-health-related outcomes in adulthood [108]. In humans, both low and high fetal iron status were associated with poor intelligence at the age of five [109], and administration of high-iron-fortified formula to infants without anemia was associated with poor performance across multiple neurodevelopmental outcomes assessed at childhood [110], adolescence [111] and young adulthood [112]. Finally, while epidemiological and translational data indicate that maternal and perinatal iron deficiency may predispose to schizophrenia [113–115], animal data reveal that upon institution of iron-replete diets, early iron deficiency may rapidly turn into excessive iron accumulation in the PFC and other brain regions [116, 117]. Thus, whether representing a primary perturbation or reflecting a compensatory event following earlier iron deficiency, our findings are consistent with the premise that, in addition to the well-appreciated effects of iron deficiency, abnormally elevated levels of iron during neurodevelopment could also exert long-lasting deleterious effects on the brain.

While iron-related genes that alter schizophrenia risk are yet to be identified (Table S45) [118], iron physiology has predominantly been characterized on the somatic side of the BBB. Brain iron homeostasis is tightly regulated but is far from fully characterized. We hypothesize that the genetics of schizophrenia, being highly polymorphic, may involve functions in maintaining brain iron homeostasis that are currently unknown. With the exception of rare genetic disorders leading to brain iron accumulation and neurodegeneration [119], deciphering the genetic architecture of cortical iron regulation has proved elusive in both rodents [120] and man [121]. Moreover, interaction with environmental factors can modulate schizophrenia penetrance [122], and indeed, alongside emergence of schizophrenia-like phenotypes, social isolation in rats was recently shown to induce elevated PFC iron mirrored by hippocampal iron depletion [123]. Accordingly, it seems plausible to assume that whether genetically determined, environmentally triggered, or both, the perturbations in iron biology that we identified bear mechanistic relevance to schizophrenia. As aging is associated with cortical iron accumulation [124], the accelerated iron accumulation we observed in the schizophrenia PFC could contribute to the “older” structural [5, 53], molecular [10] and lipidome [125] brain states reported in this disorder.

As no association between variants in ferritin genes and schizophrenia has been observed (Table S45) [118], and ferritin mRNA levels were unaltered in the schizophrenia PFC (Figures S15,S16) [126], our data indicating that in patients ferritin was reduced at the protein level (Fig. 1b) is consistent with ferritin’s extensive post-transcriptional regulation. For instance, translation of ferritin mRNAs, repressed at low iron levels via binding of iron-regulatory proteins (IRPs) 1 and 2 (known as aconitase [ACO1] and IREB2, respectively) to 5′ iron-responsive elements, is enhanced when, in the face of abundant cellular iron, the IRPs’ binding affinities decrease [127]. IREB2, the main translational repressor for ferritin [128], was significantly associated with schizophrenia in both CLOZUK and PGC2 genome-wide association studies [118, 129] and survived Mendelian randomization analysis [130, 131] (Table S46). Using GWAS-eQTL co-localization analyses, schizophrenia causality was recently ascribed to this gene [132]. As IREB2 was shown to be marginally overexpressed in schizophrenia PFC (Figure S17) [126], these latter findings could provide a potential link to understanding why ferritin protein levels among patients in our sample were exceptionally low in individuals with below-mean iron content (Fig. 3a).

Accelerated autophagic degradation of ferritin (ferritinophagy) could contribute to the observed reduction in ferritin protein levels (Fig. 1b). Lipid peroxidation products have been shown to activate autophagy, including a robust increase in microtubule-associated protein 1 light chain 3 (LC3), which constitutes a key component of autophagosomes [133]. As cytosolic ferritin was shown to colocalize with LC3 [134], the accelerated lipid peroxidation that has been reported in schizophrenia [135] could lead to excessive ferritinophagy, perhaps as part of a broader dysregulation in autophagy reported in schizophrenia [136, 137]. Of note, nuclear receptor coactivator 4 (NCOA4), previously identified as ferritin’s canonical cargo autophagy receptor [138], was recently shown to drive ferritin phase separation by facilitating formation of liquid-like NCOA4-ferritin condensates [139, 140]. While soluble NCOA4 and ferritin are degraded by the macroautophagy pathway under iron depletion, (paradoxical) delivery of NCOA4-ferritin condensates to the lysosome by a TAX1BP1-dependent non-canonical autophagy pathway could emerge under prolonged iron repletion [141]. Despite elevated tissue iron (Fig. 1a), in light of a nominal genetic association between *TAX1BP1* and schizophrenia (Table S45) [118], alongside hypomethylation (Table S47) [142] and overexpression (Figure S18) [126] in PFC tissue of schizophrenia patients, a pathological upregulation of non-canonical ferritinophagy in schizophrenia could contribute to the ferritin deficiency we observed. Finally, as ferritin can also be degraded via the ubiquitin proteasomal system [143], an enhanced ubiquitination profile recently reported in schizophrenia [144, 145] could also contribute to the observed ferritin deficiency

Consistent with ferritin’s neuroprotection against iron-mediated build-up of toxic lipid peroxides [146], increased lipid peroxidation has been reported in schizophrenia [135], coupled with impaired synthesis of glutathione necessary for detoxifying lipid peroxides [147, 148]. A recent imaging study demonstrated rapid glutathione depletion early in the natural history of schizophrenia, coinciding with the emergence of debilitating symptoms [149], and, notably, with a peak in iron-related disease risk reported above (Fig. 2). Glutathione deficiency has also been reported in the PFC of people with schizophrenia [150] in a cohort overlapping the current (Table S1). As neuroinflammation, oxidative stress, and lipid peroxidation were shown to perturb synaptic plasticity, it is conceivable that in some vulnerable individuals, pathological iron accumulation during neurodevelopment, coupled with impaired capacity for handling labile iron (e.g., ferritin deficiency) and for offsetting toxic lipid peroxides (e.g., glutathione), could push the prefrontal tissue above a threshold that elicits accelerated grey matter loss. In a subset of patients, ongoing iron-dependent lipid peroxidation could lead to progressive deficits [151], and indeed, up-regulation of cell death-related transcripts has also been reported in the PFC of people with schizophrenia [152] in a cohort overlapping the current (Table S1).

During adolescence, excitatory synapses in the PFC may be pruned to generate a proper excitatory/inhibitory balance [50], and an optimal level of dopamine stimulation is required for the proper shaping of synaptic connectivity [153]. Iron serves as a major cofactor for tyrosine hydroxylase, the rate-limiting enzyme in catecholamine synthesis [154], so that PFC iron dyshomeostasis during late neurodevelopment could tip mesocortical dopaminergic stimulation away from its optimal balance and alter synaptic maturation. The aberrant iron biology in the young-adult PFC could also be relevant to other neurochemical events germane to schizophrenia. For instance, iron-induced lipid peroxidation was shown to inhibit dopamine synthesis [155], while the activity of monoamine oxidase B (MAO-B), which plays a major role in catalysing dopamine degradation in the adult cortex [156, 157], is markedly enhanced by iron [158], providing mechanistic links between iron accumulation and the increasingly appreciated role of cortical *hypo*dopaminergia in schizophrenia [159, 160]. Moreover, through its regulation of aconitase 1 (IRP1) activity, a prominent role for iron in modulating glutamate production and secretion has also been reported [161]. Effects of iron on dopamine receptors, on levels of other monoamines and their transporters and on trophic factors have also been described, offering numerous hypothetical links between cortical iron elevation and cognitive-emotional phenotypes [162].

An additional mechanism linking our findings to schizophrenia reflects iron’s emerging roles in neuronal signalling. Cross-talk between N-methyl-D-aspartate receptors (NMDARs) and iron [163], whereby NMDARs mediated neuronal iron homeostasis [164, 165], and iron, in turn, reduced NMDAR-dependent excitability [166], has been implicated in phenotypes related to anxiety and schizophrenia [167]. While ferritin-bound iron is “nanocaged” and largely excluded from participating in such intracellular signalling, part of the excessive PFC iron that we noted may find its way into lysosomes, where ferritin is degraded and iron is released [168]. Thus, the observation that NMDAR-mediated excitation was inhibited by iron that originated from lysosomal storage [166] seems highly relevant to our findings.

Lack of data addressing iron dyshomeostasis in specific cell populations represents a limitation of the current study. For instance, proteomics analyses of the schizophrenia corpus callosum unravelled lower levels of both heavy- and light-chain ferritin [169, 170], indicating possible iron perturbations in oligodendrocytes, the principal iron-staining cell in white matter. Future in-depth assessment of iron neurochemistry in post-mortem schizophrenia tissue is thus warranted. Limited spatial measurement is another limitation of the current study, as iron [51] content in the PFC and in relevant subcortical regions may be weakly correlated [68, 120]. Future measurement of iron levels across numerous brain regions could also be guided by genetic findings, such as a recently reported association between predisposition to low dopamine, PFC iron accumulation, and working memory deficits [171].

We note that of the total tissue iron that we assayed upon acid extraction, only a minor fraction that remains unbound to proteins is implicated in oxidative damage. Unfortunately, this labile iron pool is not easily measured in post-mortem tissue, leaving as analytical markers of cell iron status the total iron (associated with the minor labile iron pool as well as the predominant soluble protein fraction including ferritin) and ferritin itself. The concentration of the labile iron pool is kept steady by mechanisms such as ferritin expression [172], and the suppression of ferritin in schizophrenia might thus increase this potentially adverse pool. However, the possibility that the labile iron pool is decreased in schizophrenia (so lowering ferritin translation and expression), while iron might be abnormally accumulating in yet unidentified pools, cannot yet be excluded. Indeed, a repartitioning of total iron out of ferritin and into redox-active high and low molecular weight fractions has been observed in the *C. elegans* model of aging [173, 174], and similar changes may explain our current findings. Finally, while an assumption that an imbalance between total cell iron cell and ferritin might be potentially toxic seems plausible from a theoretical perspective, our data did not provide direct evidence indicating that such imbalance is biologically deleterious. To this end, future studies employing relevant model systems while examining more direct markers of iron-mediated oxidative damage could directly implicate catalytic or labile iron in schizophrenia-like pathophysiology.

Despite decades-long research, major culprits driving prefrontal deficits in individuals with schizophrenia remain elusive. Focusing on iron biology, in the current study we showed that compared to matched controls, prefrontal tissue from schizophrenia cases was characterised by elevated iron, diminished ferritin, and most notably, an elevated iron-to-ferritin ratio. Using a prediction model based on iron and ferritin, our data are consistent with a potential pathophysiologic link between perturbed cortical iron biology and schizophrenia. As a prognostic biomarker, imaging cortical iron in-vivo could shed light on a potential relationship between iron, clinical course and treatment response, as was recently reported with early psychosis glutathione imaging [175]. Therapeutically, deferiprone, a BBB-permeating iron chelator traditionally used to treat peripheral iron overload [176], has been investigated for the treatment of aceruloplasminemia, pantothenate kinase–associated neurodegeneration, Friedreich’s ataxia, and PD, with preliminary evidence indicating that removal of excess iron from disease-implicated brain regions is accompanied by slowing of disease progression [177–181]. Taken together, our findings could provide a rationale for testing iron-modifying interventions, such as deferiprone, with the aim to restore healthy cortical iron biology and improve cortical function in schizophrenia.

### Supplementary information

SUPPLEMENTAL MATERIAL

Dataset and code

## Data Availability

All data and statistical code relating to the analyses reported in this manuscript are available in the Supplementary Data File (Excel format). Additional methods are elaborated in the Supplementary Methods and Tables.
