# Supplementary material for: Perturbed iron biology in the prefrontal cortex of people with schizophrenia
Source: Mol Psychiatry. 2023 Feb 7;28(5):2058–70. doi: 10.1038/s41380-023-01979-3 (PMC10575779; doi:10.1038/s41380-023-01979-3)
Supplement: Supplementary file 1 — SUPPLEMENTAL MATERIAL [file 41380_2023_1979_MOESM1_ESM.pdf]

# - SUPPLEMENTARY INFORMATION -

Perturbed Iron Biology in the Prefrontal Cortex of People with Schizophrenia

Amit Lotan, Sandra Luza, Carlos M. Opazo, Scott Ayton, Darius Lane, Serafino Mancuso, Avril Pereira, Suresh Sundram, Cynthia Shannon Weickert, Chad Bousman, Christos Pantelis, Ian P. Everall, Ashley I. Bush

Supplementary Methods (pp. 3-9)

Brain samples.

Tissue collection.

Statistical analysis.

Supplementary Tables (pp. 10-59)

Table S1. Clinical and postmortem characteristics of samples obtained from three brain banks.

Table S2. Disease-related characteristics of patients in our samples.

Table S3. Antibodies used for detection and quantification of ferritin in brain tissue.

Table S4. Robust normality statistics for iron distribution in the Combined Cohort, grouped by diagnosis.

Table S5. Robust between-group comparison of PFC iron levels in the Combined Cohort, visualized in Figure 1a.

Table S6. Robust measures of iron distribution in the Combined Cohort.

Table S7. Predicting iron content by diagnosis, controlling for demographic and tissue-quality variables one at a time and simultaneously.

Table S8. PFC iron across diagnosis based on regression-adjusted propensity-score matching.

Table S9. PFC iron across diagnosis based on regression-adjusted propensity-score matching, including mode of death as a covariate.

Table S10. Predicting iron content by diagnosis, controlling for smoking.

Table S11. Predicting iron content by diagnosis, controlling for alcohol use.

Table S12. Predicting disease status based on adjusted PFC iron, controlling for BMI.

Table S13. Predicting iron content by antipsychotic treatment.

Table S14. Predicting iron content by antipsychotic treatment, controlling for onset age and duration of illness.

Table S15. Predicting iron content by type of antipsychotics used.

Table S16. Predicting iron content by results of a postmortem toxicology essay for antipsychotics.

Table S17. Robust normality statistics for copper distribution in the Combined Cohort, grouped by diagnosis.

Table S18. The effect of diagnosis on PFC copper levels in the Combined Cohort, related to Figure S7a.

Table S19. Robust measures of copper distribution in the Combined Cohort.

Table S20. Predicting iron content by copper, controlling for diagnosis and copper × diagnosis interaction term, related to Figure S7b.

Table S21. Robust normality statistics for zinc distribution in the Combined Cohort, grouped by diagnosis.

Table S22. The effect of diagnosis on PFC zinc levels in the Combined Cohort, related to Figure S8a.

Table S23. Robust measures of zinc distribution in the Combined Cohort.

Table S24. Predicting zinc content by iron, controlling for diagnosis, related to Figure S8b.

Table S25. Exploring the effect of iron on diagnosis through zinc, related to Figure S9.

Table S26. Robust normality statistics for ferritin distribution in the Combined Cohort, grouped by diagnosis.

Table S27. Robust between-group comparison of PFC ferritin levels in the Combined Cohort, visualized in Figure 1b.

Table S28. Robust between-group comparison of PFC ferritin levels in the Combined Cohort, controlling for covariates.

Table S29. Robust between-group comparison of PFC iron-to-ferritin ratio in the Combined Cohort, visualized in Figure 1c.

Table S30. Robust analysis of the relationship between PFC iron and age among control individuals, visualized in Figure 2a.

Table S31. Robust analysis of the relationship between PFC iron and age among schizophrenia cases, visualized in Figure 2a.

Table S32. Logistic regression analyses predicting disease status based on PFC iron for sequential age cutoffs, visualized in Figure 2b.

Table S33. Logistic regression analyses predicting disease status based on PFC iron in young and old subcohorts, visualized in Figure 2c.

Table S34. Robust between-group comparison of PFC iron levels in the young and old subcohorts, visualized in Figure 2d.

Table S35. Robust analysis of the relationship between PFC ferritin and iron among control individuals, visualized in Figure 3a.

Table S36. Robust analysis of the relationship between PFC ferritin and iron among schizophrenia cases, visualized in Figure 3a.

Table S37. Predicting ferritin content by iron, controlling for diagnosis and iron × diagnosis interaction term, visualized in Figure 3a.

Table S38. Logistic regression analyses predicting disease status based on PFC ferritin for sequential iron cutoffs, visualized in Figure 2b.

Table S39. Logistic regression analyses predicting disease status based on PFC ferritin in low-iron and high-iron subcohorts, visualized in Figure 3c.

Table S40. Robust between-group comparison of PFC ferritin in low-iron and high-iron subcohorts, visualized in Figure 3d.

Table S41. Deriving optimal cutpoints, visualized in Figure 4a-b.

Table S42. Logistic regression model predicting disease based on a combination of three predictors following definition of optimal cutpoints.

Table S43. Likelihood-ratio tests comparing nested and full regression models.

Table S44. Deriving an optimal cutoff for a logistic regression model combining all three predictors.

Table S45. Genome-wide association of iron regulatory proteins and schizophrenia based on GWAS results of CLOZUK.

Table S46. *IREB2* - Association with schizophrenia and integrative (Mendelian randomization) analysis.

Table S47. Differential methylation of *TAX1BP1* in schizophrenia prefrontal cortex.

Supplementary Figures (pp. 60-77)

Figure S1. Comparison of prefrontal iron content across diagnostic groups, related to Figure 1.

Figure S2. Iron distribution in the Combined Cohort across diagnostic groups, related to Figure 1.

Figure S3. Iron distribution in the Combined Cohort, related to Figure 1.

Figure S4. Balancing plots for PFC iron across diagnosis based on regression-adjusted propensity-score matching, related to Figure 1.

Figure S5. Balancing plots for PFC iron across diagnosis based on regression-adjusted propensity-score matching, including death circumstances as a covariate, related to Figure 1.

Figure S6. Predicting iron content by antipsychotic treatment, related to Figure 1.

Figure S7. Prefrontal copper across diagnostic groups and its effect on iron.

Figure S8. Prefrontal zinc across diagnostic groups and its dependence on iron.

Figure S9. Exploring the effect of iron on diagnosis through zinc.

Figure S10. Ferritin across diagnostic groups, related to Figure 1.

Figure S11. Identifying highly influential/ irregular observations, related to Figure 2.

Figure S12. Identifying highly influential/ irregular observations, related to Figure 3.

Figure S13. Discriminating schizophrenia patients from controls based on prediction models, related to Figure 4.

Figure S14. Logistic regression model predicting schizophrenia based on iron-to-ferritin ratio, related to Figure 4.

Figure S15. Differential expression of FTH1 in schizophrenia prefrontal cortex.

Figure S16. Differential expression of FTL in schizophrenia prefrontal cortex.

Figure S17. Differential expression of IREB2 in schizophrenia prefrontal cortex.

Figure S18. Differential expression of TAX1BP1 in schizophrenia prefrontal cortex.

Supplementary References (pp. 78-79)

Supplementary Data File (Excel format, online)

Sheet 1: Raw data

Sheet 2: Stata code

Supplementary Methods

Brain samples

New South Wales Brain Tissue Resource Center (NSW-BTRC)

Frozen postmortem tissue was obtained from the New South Wales Brain Tissue Resource Center (Sydney, Australia). As previously described[1], cases with an unclear psychiatric diagnosis, evidence of cocaine or phencyclidine (PCP) abuse by history and/or toxicology, cerebrovascular disease, autolysis, subdural hematoma, neuritic pathology or other pathological features were excluded from the cohort. Unaffected controls were screened by telephone interviews of family members and/or police records for a history of medical and/or psychiatric problems, including alcohol abuse and illicit drug use. Any positive history of a psychiatric problem or excessive alcohol or drug use led to the exclusion of that participant from the normal control group.

Victoria Brain Bank Network (VBBN)

As previously described[2], human post-mortem CNS tissue was collected after obtaining approval from the Ethics Committee of the Victorian Institute of Forensic Medicine and gaining written consent from the nearest next-of-kin. Following tissue collection, case history reviews were conducted using the Diagnostic Instrument for Brain Studies[3,4] a post-mortem assessment tool which enables a diagnostic consensus to be reached using DSM-IV criteria[5]. Using data from the Diagnostic Instrument for Brain Studies, duration of illness (DI) was calculated as the time from first hospitalisation to death and post-mortem interval (PMI) was calculated as the time from death to autopsy. Where death was not witnessed, tissue was only collected from subjects who had been seen alive up to 5 h prior to being found dead. In these instances, the PMI was taken as the midpoint between the person being found dead and last seen alive. All cadavers from which tissue was collected were refrigerated within 5 h of being found to minimise the impact of tissue processing variables.

NIMH Human Brain Collection Core (NIMH-HBCC)

Brains were obtained through the Offices of the Chief Medical Examiner (OCMEs) of Virginia and the District of Columbia. Information about the decedent's medical and psychiatric history was obtained through interviews with the family and released medical records after obtaining written authorization from the next-of-kin. Patients with schizophrenia were matched by age, sex, PMI, pH and RIN to the controls. No subject in this cohort had a substance abuse diagnosis during their

lifetime. All procedures were approved by the HBCC Oversight Committee and by the NIH Department of Bioethics.

Tissue collection

New South Wales Brain Tissue Resource Center (NSW-BTRC)

As previously described[1], in RNAse-free conditions, blocks for tissue sections (14 µm) and for pulverisation/homogenisation were dissected from fresh frozen coronal slabs of prefrontal cortex which were identified as being on the ventral surface below the inferior frontal gyrus, and anterior to the appearance of the lateral ventricle. All blocks for sectioning contained gyrus rectus (Brodmann area [BA] 11) on the medial side. For homogenisation, approximately 0.5 g of predominantly grey matter tissue was excised on a dry ice platform using a dental drill (Cat# UP500-UG33, Brasseler, USA) at moderate speed (up to 40,000 rpm) to minimize heat generation during the dissection. Tissue from each case was then pulverised over a frozen tray placed in dry ice, weighed while frozen, and stored at –80ºC pending further analysis.

Victoria Brain Bank Network (VBBN)

The left hemisphere was removed at autopsy, rapidly processed and frozen to −80 °C using a standardised procedure[6] by the same individual in a way designed to minimise autolytic effects[7]. The pH of the brain tissue (BA 11) was measured as described previously[8] as this is the best indicator of tissue preservation[9].

NIMH Human Brain Collection Core (NIMH-HBCC)

Each brain was sliced into ~1 cm slabs, which were barcoded and stored in individual plastic bags in -80°C freezers until dissections of circumscribed brain regions (BA 10) were performed using a dental drill or a scalpel. Whole blood was sent to the National Medical Services Lab for toxicological analysis.

Statistical analysis

Preliminary analysis and data transformation

Demographic and tissue-quality variables were compared across diagnostic groups using independent-samples t-tests for continuous variables or Pearson's chi^2 test (Fisher's exact when expected cell counts<5) for categorical variables. Preliminary analyses were performed separately

for the NSW-BTRC samples and jointly for VBBN+NIMH-HBCC samples, as the latter were analyzed later and the VBBN group was relatively small to be analyzed on its own. For assessing differences in iron levels across diagnostic groups we used an independent-samples t-test with Welch correction (NSW-BTRC) and an ANCOVA (joint samples from VBBN+NIMH-HBCC, controlling for tissue origin) with robust standard errors. Following replication of our major iron finding in both preliminary analyses, we decided to generate a Combined Cohort based on control-derived z-scores. Specifically, using in-house Stata code, for each cohort separately we performed a z-score transformation on the data of the control group, and then adjusted the values of the schizophrenia cases group based on the control group's mean and standard deviation (SD). All subsequent analyses were carried out on z-scores across the entire Combined Cohort, except for specific endpoints (e.g. copper levels, iron levels among smokers) that were measured only in subsets of the combined cohort. In the case of heavily skewed distributions (as opposed to relatively normal distributions containing outliers), such as lifetime cumulative antipsychotic dose, a log-transformation was applied.

Linear regression models

Assessing covariates. Demographic data (i.e. sex, age, race, death circumstances [non-suicide vs. suicide]) and tissue-quality parameters (i.e. pH and postmortem interval) were available for all specimens. Additional demographic data (i.e. smoking, weight and BMI), disease-related data (age-of-onset, duration of illness, mean-daily and lifetime-cumulative doses of antipsychotics) and postmortem toxicology data were only available for subsets of individuals. Given its central role in the manuscript, for iron we performed robust linear regressions assessing the importance and effect of each covariate, by running a series of regressions where diagnosis and other covariates serve as predictors and assessing both inference statistics regarding the covariate and change in point statistics of diagnosis coefficient attributed to the addition of covariates. In the case of iron, we also assessed the effect of diagnosis following propensity score matching (also considering the death circumstances) using Stata's 'kmatch' command.

Rationale for robustness. In regression analysis, the presence of outliers in the dataset can strongly distort the classical least-squares estimator and lead to unreliable results. To deal with this, several robust-to-outliers methods have been proposed in the statistical literature. When initially fitting a least squares (OLS) regression to our data, we found some outliers and high leverage data points. We have decided that these data points were not data entry errors, neither were they from a different population than most of our data, so we had no compelling reason to exclude them from the analysis. In this scenario, robust regression seemed to be a good strategy since it is a compromise

between excluding these points entirely or alternatively including all the data points while treating them equally using OLS regression. The idea of robust regression is to weigh the observations differently based on how "well behaved" these observations are. In this sense, it is a form of weighted and reweighted least squares regression. In recent years, it seems that a consensus has emerged to recommend the MM-estimators as a well-suited estimation method, because these estimators combine a high resistance to outliers and high efficiency[10].

ROBREG. Extending the conceptual framework laid by Verardi et al[10], throughout our analysis we have used "ROBREG: Stata module providing robust regression estimators,"[11]. Specifically, we utilized the "robreg mm" command, which fits an efficient high breakdown MM-estimator. On the first stage, a high breakdown S-estimator (using default breakdown point of 0.5) is applied to estimate the residual scale and derive starting values for the coefficients vector. On the second stage, using iteratively reweighted least squares (IRWLS), an efficient bisquare MM-estimator is applied to obtain the final coefficient estimates. Retaining efficiency (set at 95%), this regression is considered robust to both vertical and high-leverage (i.e., influential) outliers. Stata's robust standard errors can also effectively deal with minor failures in meeting other assumptions such as normality of residuals. The robreg command has been used throughout most linear model analyses, including between-group comparisons (iron, ferritin and iron-to-ferritin ratio) and relationships (e.g., antipsychotic dose-iron, age-iron, iron-ferritin, iron-copper, iron-zinc). ROBREG computes, by default, robust standard errors, which account for heteroscedasticity in regression, and thus also addresses cases of unequal variances between two-groups (e.g., iron in cases vs. controls).

ROBSTAT. As for the assumption of normality of residuals, classical statistical tests proposed to find out whether a sample is drawn from a normal distribution or not, such as the Jarque-Bera test, are based on moments of the data, yielding a zero-breakdown value (i.e., a single outlier can make the test worthless). Thus, it has been proposed that following a robust regression procedure, normality of the residuals will subsequently be tested using a robust test for normality. These robust normality tests were executed in Stata using "ROBSTAT: Stata module to compute robust univariate statistics"[12].

FRACPOLY. Age-iron and iron-ferritin relationships were examined using Stata's "fracpoly" command, with models based on logarithmic, square-root, quadratic and cubic transformations of the independent variable compared to non-transformed models using Akaike's information criterion and Bayesian information criterion. Models with the highest information criteria were selected.

Logistic regression and ensuing prediction models

As in linear regression, influential data points may yield biased regression coefficient estimates in logistic regression as well. However, given limited outlier-robust alternatives to standard logistic regression, we decided to identify (and exclude) influential observations and outliers using Pregibon's dbeta ("predict dbeta") which provides summary information of influence on parameter estimates of each individual observation (more precisely each covariate pattern) and is very similar to Cook's D in ordinary linear regression. For assessing if the effect of iron on diagnosis was mediated via zinc, a generalized structural equation model (GSEM) with a logit link function for the dependent variable was built, with significance of indirect effect quantified via bootstrapped-derived confidence intervals. For determining discriminatory performance, receiver operating characteristic (ROC) curve analyses were performed, and optimal cutpoints were empirically determined using Stata's 'cutpt' command (Youden method). Following categorical transformation of continuous predictors using these cutpoints, the simultaneous contribution i) of iron among the subcohort of individuals who had died younger than 35 years; ii) of ferritin among the subcohort of individuals with below-mean iron and iii) of iron-to-ferritin ratio among the entire cohort was assessed using a multiple logistic regression model with categorical predictors denoting high-risk, low-risk and unavailable-data categories. The discriminatory performance of this model was compared to nested models through likelihood-ratio-test chi-square statistics.

Data mining

Mining data relating to genetic association and differential expression and methylation was performed using SZDB2.0: an updated comprehensive resource for schizophrenia research[13]. For assessing whether genes coding for of iron regulatory proteins were associated with schizophrenia, genes from a list of iron regulatory genes based on the study by McAllum et al[14] were mined in the CLOZUK GWAS[15], where a genome-wide significant association with schizophrenia was defined as a $P < 5 \times 10^{-8}$. For *IREB2*, significance of a Summary data–based Mendelian (SMR) randomization analysis (P_{SMR})[16] was mined. For *TAX1BP1*, differential methylation data[17] were mined. For FTH1, FTL, IREB2 and TAX1BP1, differential expression in the schizophrenia PFC[18] was mined.

Supplementary Tables

	NSW-BTRC New South Wales Brain Tissue Resource Center			VBBN Victoria Brain Bank Network			NIMH-HBCC National Institute of Mental Health Human Brain Collection Core		
	**Con**	**Scz**	**p[1]**	**Con**	**Scz**	**p[1]**	**Con**	**Scz**	**p[1]**
Number of samples from which iron and were quantified	37	38	-	18	19	-	30	29	-
Age, mean (sd) years	52.5 (14.7)	52.1 (14.4)	0.891	58.8 (10.4)	50.5 (18.0)	0.096	54.1 (17.1)	54.6 (17.6)	0.912
Sex, % (n) female	27 (10)	34 (13)	0.500	17 (3)	16 (3)	1.000	37 (11)	38 (11)	0.920
Ethnicity, % (n) Caucasian	100 (37)	97 (37)	1.000[2]	100 (18)	100 (19)	1.000[2]	47 (14)	48 (14)	0.902
Smokers[3], % (n/N[4])	42 (11/26)	76 (22/29)	**0.011**	~	~	~	24 (7/29)	72 (21/29)	**<0.001**
Alcohol users[5], % (n/N[4])	22 (5/23)	47 (15/32)	0.058	~	~	~	~	~	~
BMI, mean (sd) kg/m^2	~	~	~	~	~	~	29.3 (6.1)	27.9 (6.2)	0.376
pH, mean (sd)	6.68 (0.28)	6.61 (0.30)	0.334	6.42 (0.24)	6.36 (0.24)	0.452	6.46 (0.28)	6.41 (0.21)	0.429
PMI, mean (sd) hours	26.4 (11.8)	28.2 (13.6)	0.534	45.6 (13.0)	45.6 (13.0)	0.991	33.8 (15.9)	41.3 (22.4)	0.139
Number of samples from which copper was quantified[6]	36	38		0	0		30	29	
Ethics Committee responsible for study approval	University of New South Wales (HREC 07261)			Victorian Institute of Forensic Medicine; Tissue Access Committee of the VBBN			HBCC Oversight Committee; NIH Department of Bioethics		

Table S1

Clinical and postmortem characteristics of samples obtained from three brain banks.

[1]Significance of between-group (control vs. Scz) comparison based on independent-samples t-test (continuous variables) and Pearson -chi^2 (categorical variables).

[2]Pearson -chi^2 was replaced by Fisher's exact test due to expected cell count<5.

[3]Smokers were defined as current smokers, heavy ex-smokers, or individuals with a positive postmortem toxicology essay for nicotine.

[4]N, number of individuals for whom data on smoking habits or alcohol use were available.

[5]Alcohol users were defined as those with a history of drinking an average of ≥20 g ethanol/day.

[6]Copper was not measured in specimens from the Victoria Brain Bank Network (VBBN) as the glycerol-containing extraction buffer used during homogenization of these samples interfere with copper and thus bias its quantification. Copper value obtained from one of the NSW-BTRC control specimens was unreliable and thus excluded.

~Unavailable data. BMI, body mass index; PMI, post-mortem interval.

	NSW-BTRC	VBBN	NIMH-HBCC	Number of individuals for which data were available			
				**NSW**	**VBBN**	**NIMH**	**Total**
Age of onset, mean (sd) years	23.8 (6.2)	28.9 (12.7)	-	35	19	0	54
Duration of illness, mean (sd) years	27.1 (14.0)	21.8 (14.9)	-	35	19	0	54
[1]Exposure to atypical antipsychotics, % (n)	80.0 (28)	83.3 (10)	-	35	12	0	47
[2]Antipsychotic dose, mean (sd) CPZ-equivalents, mg/day	677 (506)	535 (440)	-	35	8	0	43
[2]Cumulative antipsychotic exposure, mean (sd) log_{10}(CPZ-equivalents [mg/day] x time [days])	15.3 (1.2)	14.4 (1.4)	-	35	8	0	43
Presence of antipsychotic drug on postmortem toxicology essay, % (n)	47.1 (16)	-	65.5 (19)	34	0	29	63

Table S2

Disease-related characteristics of patients in our samples.

[1]Dichotomous variable indicating whether antipsychotic treatment had always been limited to typical agents or whether it had also included use of atypical agents.

[2]Current/last CPZ equivalent dosage was calculated in all patients in all cohorts by following standard guidelines[19,20], included were cases with mean CPZ-equivalents $\geq$ 50 mg/day. Due to the large range over which data were dispersed and right-skewness, log-transformation was applied.

CPZ, chlorpromazine.

Antibody	Host	Dilution	Source	Catalogue #	Remarks
Ferritin	Rabbit	1: 1,000	Abcam	Ab75973	In human recognized both heavy and light chains
GAPDH	Mouse	1: 10,000	Sigma-Aldrich	G8795	
Anti-mouse IRDye (680RD; 800CW)	Goat	1:10,000	Li-Cor, Lincoln	Ab216776<br>Ab216772	
Anti-rabbit IRDye (680Rd; 800CW)	Goat	1:10,000	Li-Cor, Lincoln	Ab216777<br>Ab216773	

Table S3

Antibodies used for detection and quantification of ferritin in brain tissue.

Robust Statistics   Number of obs = 171

0: dx = Controls
1: dx = Scz

ziron	Coef.	Std. Err.	[95% Conf.	Interval]
0				
skewness	.6465839	.168508	.3139463	.9792215
kurtosis	3.870133	.4803464	2.921921	4.818345
SK25	.0346459	.0723053	-.1080861	.1773778
QW25	1.436813	.1134481	1.212864	1.660761
MC	.0897035	.060244	-.0292192	.2086262
LMC	.0540827	.0948149	-.1330834	.2412488
RMC	.2544658	.0902236	.0763628	.4325688
1				
skewness	.8057016	.0992231	.6098336	1.00157
kurtosis	3.365869	.2563931	2.859745	3.871993
SK25	.2146485	.0709307	.07463	.354667
QW25	1.348894	.110954	1.129869	1.567919
MC	.1235471	.0618757	.0014033	.2456908
LMC	.2469048	.0899998	.0692437	.424566
RMC	.0533033	.1039351	-.1518663	.2584729

Normality Tests

	chi2	df	Prob>chi2
0			
JB	8.60	2	0.0135
MOORS	1.21	2	0.5452
MC-LR	1.73	3	0.6309
1			
JB	9.78	2	0.0075
MOORS	2.54	2	0.2807
MC-LR	1.50	3	0.6819

Table S4

Robust normality statistics for iron distribution in the Combined Cohort grouped by diagnosis, visualized in Figure S2a.

Generalized Jarque-Bera tests for normality (right), as suggested by Brys et al.[21], were derived using ROBSTAT, a Stata module to compute robust univariate statistics[12]. **JB**, classic Jarque-Bera normality test based on skewness and kurtosis[22]; SK25, Yule and Kendall skewness measure; QW25, quantile tail weight measure, at 25% (default); MC, medcouple tail weight measure; LMC, left medcouple tail weight measure; RMC, right medcouple tail weight measure. **MOORS**, robust skewness and tail-weight normality test based on SK25 and QW25[23]. **MC-LR**, robust skewness and tail-weight normality test based on MC, LMC and RMC[21]. Based on the robust versions, iron was normally distributed in both groups. $n_{controls}$=85, n_{Scz}=86.

a

```
MM regression (95% efficiency)                    Number of obs     =        171
                                                  Wald chi2(1)      =       8.29
                                                  Prob > chi2       =     0.0040
                                                  Pseudo R2         =     0.0440
                                                  Breakdown point   =         50
                                                  M-estimate: k     =  4.6850649
                                                  S-estimate: k     =   1.547645
                                                  Scale             =  1.1491248
```

ziron	Coef.	Robust Std. Err.	t	P>|t|	[95% Conf.	Interval]
dx	.577584	.2006151	2.88	0.005	.1815496	.9736183
_cons	-.0421633	.1075615	-0.39	0.696	-.2545005	.1701738

b

```
Adjusted predictions                              Number of obs     =        171
Model VCE    : Robust

Expression   : Fitted values, predict()
```

	Margin	Delta-method Std. Err.	z	P>|z|	[95% Conf.	Interval]
dx						
Controls	-.0421633	.1075615	-0.39	0.695	-.2529799	.1686533
Scz	.5354206	.1760955	3.04	0.002	.1902797	.8805616

Table S5

Robust between-group comparison of PFC iron levels in the Combined Cohort, visualized in Figures 1a and S2b.

(a) The effect of diagnosis on iron. Robust regression estimates predicting PFC iron content (ziron, control-derived z-scores) by diagnosis. Regression coefficients, robust standard errors, t-statistics along with their corresponding significance and 95%CI are presented, based on a linear regression model using iteratively reweighted least squares MM-estimators, derived using Stata's "robreg mm" command (95% efficiency, default parameters)[24]. (b) Marginal means, std. errors, t-statistic and 95%CIs for iron in each diagnostic group, based on above regression and robust variance-covariance estimates. $n_{controls}$=85, n_{Scz}=86.

a

```
Summary for variables: ziron
     by categories of: dx (Diagnosis)

      dx |        sd  variance
---------+--------------------
Controls |         1         1
     Scz |  1.513347   2.29022
---------+--------------------
```

b

```
            |          Summary of ziron
  Diagnosis |        Mean   Std. Dev.       Freq.
------------+------------------------------------
   Controls |  -6.355e-10   .99999999          85
        Scz |   .73955856   1.5133472          86
------------+------------------------------------
      Total |   .37194173   1.3329531         171

W0  = 11.6460821   df(1, 169)     Pr > F = 0.00080518

W50 =  9.8442205   df(1, 169)     Pr > F = 0.00201037

W10 = 10.5241049   df(1, 169)     Pr > F = 0.00142017
```

Table S6

Robust measures of iron distribution in the Combined Cohort, visualized in Figure S3.

(a) Standard deviation and variance of PFC iron among diagnostic groups, as visualized in Figure S3a. (b) Robust tests of the hypothesis that the variance of ziron is the same across groups, including Levene's statistic (W0) and two statistics proposed by Brown and Forsythe that replace the mean in Levene's formula with alternative location estimators. The first alternative (W50) replaces the mean with the median. The second alternative replaces the mean with the 10 percent trimmed mean (W10). Note that the difference between groups remained prominent across both robust analyses, indicating that the increased iron heterogeneity in schizophrenia patients was not (primarily) driven by outliers, and thus likely represents a true biological difference among diagnostic groups. $n_{controls}$=85, n_{Scz}=86.

a

Row	Predictor 1	Predictor 2	t-statistic (diagnosis)	$P_{t\text{-statistic}}$ (diagnosis)	t-statistic (predictor 2)	$P_{t\text{-statistic}}$ (predictor 2)
1	Diagnosis	-	2.88	**0.007**	-	-
2	"	Age	2.94	**0.004**	1.28	0.201
3	"	Sex (male)	2.88	**0.004**	-0.96	0.339
4	"	Ethnicity (Non-Caucasian)	2.86	**0.005**	-0.46	0.644
5	"	pH	2.52	**0.013**	-1.94	0.054
6	"	PMI	2.84	**0.005**	-0.79	0.428
**7**	"	Mode of death (Non-natural)	2.62	**0.010**	-0.30	0.765

b

```
MM regression (95% efficiency)                   Number of obs     =        171
                                                 Wald chi2(7)      =       4.61
                                                 Prob > chi2       =     0.7079
                                                 Pseudo R2         =     0.0704
                                                 Breakdown point   =         50
                                                 M-estimate: k     =  4.6850649
                                                 S-estimate: k     =   1.547645
                                                 Scale             =  1.0865149
```

ziron	Coef.	Robust Std. Err.	t	P>|t|	[95% Conf.	Interval]
dx						
Controls	0	(empty)				
Scz	.5268977	.2608973	2.02	0.045	.0117234	1.042072
age	.0032462	.0066452	0.49	0.626	-.0098756	.016368
sex						
Female	0	(empty)				
Male	-.1546183	.1889463	-0.82	0.414	-.5277163	.2184797
race01						
Caucasian	0	(empty)				
Non-Caucasian	-.1140292	.2621244	-0.44	0.664	-.6316264	.4035681
ph	-.5821794	.3912882	-1.49	0.139	-1.354827	.190468
pmi	-.0068085	.0076706	-0.89	0.376	-.021955	.008338
dm01						
Natural	0	(empty)				
Non-natural	.0089347	.2752922	0.03	0.974	-.5346641	.5525336
_cons	3.950472	2.790925	1.42	0.159	-1.560558	9.461502

Table S7

Predicting iron content by diagnosis, controlling for demographic and tissue-quality variables one at a time and simultaneously.

(a) Robust regression estimates predicting PFC iron content (control-derived z-scores) using either diagnosis alone (first row) or combinations of diagnosis and one additional predictor (Predictor 2, rows 2-7). For categorical variables, reference values of "0" were assigned to control individuals, female sex, Caucasian race, and natural death. *t*-statistics along with their corresponding significance are presented for both diagnosis and each of the other co-variates tested alongside. (b) Same as above with all covariates entered simultaneously. Based on linear regression models using iteratively reweighted least squares MM-estimators, derived using Stata's "robreg mm" command (95% efficiency, default parameters)[24]. PMI, post-mortem interval; dm01, mode of death. $n_{controls}$=85, n_{Scz}=86.

```
Propensity-score kernel matching               Number of obs      =        171
                                               Kernel             =       epan

Treatment   : dx = 1
Covariates  : age i.sex i.race01 ph pmi
PS model    : logit (pr)
RA equations: ziron = age ph pmi sex race01 _cons
```

Matching statistics

	Matched			Controls			Bandwidth
	Yes	No	Total	Used	Unused	Total	
Treated	79	7	86	81	4	85	.012866

Treatment-effects estimation

ziron	Coef.	Std. Err.	t	P>|t|	[95% Conf.	Interval]
ATT	.6878009	.1969418	3.49	0.001	.2990344	1.076567
NATE	.7395586	.1953723	3.79	0.000	.3538905	1.125227

Table S8

PFC iron across diagnosis based on regression-adjusted propensity-score matching, visualized in Figure S4.

Regression-adjusted propensity-score kernel matching with age, sex, race, pH and PMI as covariates, based on a logit model where diagnosis of schizophrenia was defined as Treatment. Seven patients could not be matched, and four controls were unused. Estimated effect of diagnosis in patients following PS-matching (ATT) was similar to the non-matched effect (NATE). Based on KMATCH, Stata's module for multivariate-distance and propensity-score matching[25]. PMI, post-mortem interval. $n_{controls}$=85, n_{Scz}=86.

```
Propensity-score kernel matching                 Number of obs     =        171
                                                 Kernel            =       epan

Treatment     : dx = 1
Covariates    : age i.sex i.race01 ph pmi i.dm01
PS model      : logit (pr)
RA equations: ziron = age sex race01 ph pmi i.dm01 _cons
```

Matching statistics

	Matched			Controls			Bandwidth
	Yes	No	Total	Used	Unused	Total	
Treated	84	2	86	83	2	85	.0402399

Treatment-effects estimation

ziron	Coef.	Std. Err.	t	P>|t|	[95% Conf.	Interval]
ATT	.5342674	.2078261	2.57	0.011	.1240151	.9445196
NATE	.7395586	.1953723	3.79	0.000	.3538905	1.125227

Table S9

PFC iron across diagnosis based on regression-adjusted propensity-score matching including mode of death as a covariate, visualized in Figure S5.

Regression-adjusted propensity-score kernel matching with age, sex, race, pH, PMI and mode of death as covariates, based on a logit model where diagnosis of schizophrenia was defined as Treatment. Two patients could not be matched, and two controls were unused. Given the gross inequality in mode of death across groups, bandwidth was considerably larger in this analysis compared to that of Table S8. Estimated effect of diagnosis in patients following PS-matching (ATT) was only slightly weaker compared to the non-matched effect (NATE). Based on KMATCH, Stata's module for multivariate-distance and propensity-score matching[25]. PMI, post-mortem interval; dm01, mode of death. $n_{controls}$=85, n_{Scz}=86.

-> dx = Controls

smoker	Freq.
No	37
Yes	18
Unknown	30
Total	85

-> dx = Scz

smoker	Freq.
No	15
Yes	43
Unknown	28
Total	86

MM regression (95% efficiency)

Number of obs	=	171
Wald chi2(3)	=	7.79
Prob > chi2	=	0.0506
Pseudo R2	=	0.0440
Breakdown point	=	50
M-estimate: k	=	4.6850649
S-estimate: k	=	1.547645
Scale	=	1.1002882

ziron	Coef.	Robust Std. Err.	t	P>|t|	[95% Conf.	Interval]
dx	.5978718	.2185192	2.74	0.007	.1664557	1.029288
smoker						
No	0	(empty)				
Yes	-.0737236	.2348611	-0.31	0.754	-.5374031	.3899558
Unknown	.0854732	.2399858	0.36	0.722	-.3883239	.5592702
_cons	-.0609283	.1493642	-0.41	0.684	-.3558137	.233957

Table S10

Predicting iron content by diagnosis, controlling for smoking.

Robust regression estimates predicting FPC iron levels (ziron, control-derived z-scores) by diagnosis controlling for smoking status (categorical predictor with categories No, Yes and Unknown). Smokers were defined as either current smokers or heavy ex-smokers and/or individuals with a positive postmortem toxicology essay for nicotine. Regression coefficients, robust standard errors, t-statistics along with their corresponding significance and 95%CI are presented, based on a linear regression model using iteratively reweighted least squares MM-estimators, derived using Stata's "robreg mm" command (95% efficiency, default parameters)[24]. $n_{controls}$=85, n_{Scz}=86.

-> dx = Controls

etoh	Freq.
No	18
Yes	5
Unknown	62
Total	85

-> dx = Scz

etoh	Freq.
No	17
Yes	15
Unknown	54
Total	86

MM regression (95% efficiency)

Number of obs	=	171
Wald chi2(3)	=	8.28
Prob > chi2	=	0.0405
Pseudo R2	=	0.0514
Breakdown point	=	50
M-estimate: k	=	4.6850649
S-estimate: k	=	1.547645
Scale	=	1.1503383

ziron	Coef.	Robust Std. Err.	t	P>|t|	[95% Conf.	Interval]
dx	.5247794	.2037879	2.58	0.011	.1224469	.9271118
etoh						
No	0	(empty)				
Yes	.3809811	.382048	1.00	0.320	-.3732852	1.135247
Unknown	-.0064053	.2196912	-0.03	0.977	-.4401353	.4273248
_cons	-.0552525	.2018147	-0.27	0.785	-.4536895	.3431844

Table S11

Predicting iron content by diagnosis, controlling for alcohol use.

Robust regression estimates predicting FPC iron levels (ziron, control-derived z-scores) by diagnosis controlling for alcohol use (categorical predictor with categories No, Yes and Unknown). Alcohol users were defined as defined as those with a history of drinking an average of ≥20 g ethanol/day. Regression coefficients, robust standard errors, t-statistics along with their corresponding significance and 95%CI are presented, based on a linear regression model using iteratively reweighted least squares MM-estimators, derived using Stata's "robreg mm" command (95% efficiency, default parameters)[24]. $n_{controls}$=85, n_{Scz}=86.

```
Logistic regression                             Number of obs     =         56
                                                Wald chi2(2)      =       5.83
                                                Prob > chi2       =     0.0543
Log pseudolikelihood =  -36.40079               Pseudo R2         =     0.0622
```

dx	Odds Ratio	Robust Std. Err.	z	P>|z|	[95% Conf.	Interval]
adj_iron	1.112217	.0518999	2.28	0.023	1.015008	1.218736
bmi	.9308275	.0451298	-1.48	0.139	.8464474	1.023619
_cons	7.786244	10.82756	1.48	0.140	.5100888	118.853

Table S12

Predicting disease status based on adjusted PFC iron, controlling for BMI.

Robust logistic regression analysis predicting disease status based on PFC iron (adj_iron, µmol/g), after iron has been adjusted for age, sex, ethnicity, sample pH and log-post-mortem interval, among 56 individuals from the New South Wales Brain Tissue Resource Centre for whom BMI data were available (Table 1). Odds ratios (for having been diagnosed with schizophrenia) per 1 µmol/g increase in adjusted iron, standard errors, z-statistics along with their corresponding significance and 95%CIs are presented. $n_{controls}$=28, n_{Scz}=28.

a

MM regression (95% efficiency)

Number of obs	=	43
Wald chi2(1)	=	0.17
Prob > chi2	=	0.6817
Pseudo R2	=	0.0047
Breakdown point	=	50
M-estimate: k	=	4.6850649
S-estimate: k	=	1.547645
Scale	=	1.4288616

ziron	Coef.	Robust Std. Err.	t	P>|t|	[95% Conf.	Interval]
log10dailydose	-.3419167	.8336801	-0.41	0.684	-2.025568	1.341734
_cons	1.693613	2.236319	0.76	0.453	-2.822725	6.209951

b

MM regression (95% efficiency)

Number of obs	=	43
Wald chi2(1)	=	0.42
Prob > chi2	=	0.5167
Pseudo R2	=	0.0082
Breakdown point	=	50
M-estimate: k	=	4.6850649
S-estimate: k	=	1.547645
Scale	=	1.3898914

ziron	Coef.	Robust Std. Err.	t	P>|t|	[95% Conf.	Interval]
log10lifetime	-.2274896	.3508486	-0.65	0.520	-.9360426	.4810635
_cons	2.253638	2.237076	1.01	0.320	-2.264228	6.771504

Table S13

Predicting iron content by antipsychotic treatment, visualized in Figure S7.

Robust regression estimates predicting PFC iron content (ziron, control-derived z-scores) by (**a**) mean antipsychotic dose (log_{10}dailydose, log_{10}[chlorpromazine equivalent, mg/day) or (**b**) cumulative antipsychotic exposure (log_{10}lifetime, log_{10}[chlorpromazine equivalent, mg/day) × (time, days]). Regression coefficients, robust standard errors, t-statistics along with their corresponding significance and 95%CI are presented, based on a linear regression model using iteratively reweighted least squares MM-estimators, derived using Stata's "robreg mm" command (95% efficiency, default parameters)[24]. n_{Scz}=43.

a

MM regression (95% efficiency)

Number of obs	=	43
Wald chi2(3)	=	0.40
Prob > chi2	=	0.9402
Pseudo R2	=	0.0084
Breakdown point	=	50
M-estimate: k	=	4.6850649
S-estimate: k	=	1.547645
Scale	=	1.4522601

ziron	Coef.	Robust Std. Err.	t	P>|t|	[95% Conf.	Interval]
log10dailydose	-.2352091	.9545412	-0.25	0.807	-2.165951	1.695533
onsetage	.0035447	.0377472	0.09	0.926	-.0728062	.0798956
duration	-.005704	.015688	-0.36	0.718	-.037436	.026028
_cons	1.466839	2.663119	0.55	0.585	-3.919827	6.853506

b

MM regression (95% efficiency)

Number of obs	=	43
Wald chi2(3)	=	0.47
Prob > chi2	=	0.9262
Pseudo R2	=	0.0088
Breakdown point	=	50
M-estimate: k	=	4.6850649
S-estimate: k	=	1.547645
Scale	=	1.3434095

ziron	Coef.	Robust Std. Err.	t	P>|t|	[95% Conf.	Interval]
log10lifetime	-.2002567	.9153252	-0.22	0.828	-2.051677	1.651163
onsetage	.0046373	.0379788	0.12	0.903	-.0721821	.0814566
duration	-.0011275	.0361034	-0.03	0.975	-.0741537	.0718986
_cons	1.991416	5.194034	0.38	0.704	-8.51451	12.49734

Table S14

Predicting iron content by antipsychotic treatment, controlling for onset age and duration of illness.

Robust regression estimates predicting PFC iron content (ziron, control-derived z-scores) by (**a**) mean antipsychotic dose (log_{10}dailydose, log_{10}[chlorpromazine equivalent, mg/day) or (**b**) cumulative antipsychotic exposure (log_{10}lifetime, log_{10}[chlorpromazine equivalent, mg/day) × (time, days]), controlling for age of disease onset (onsetage, years) and duration of illness (duration, years). Regression coefficients, robust standard errors, t-statistics along with their corresponding significance and 95%CI are presented, based on a linear regression model using iteratively reweighted least squares MM-estimators, derived using Stata's "robreg mm" command (95% efficiency, default parameters)[24]. n_{Scz}=43.

a

MM regression (95% efficiency)

Number of obs	=	43
Wald chi2(2)	=	2.15
Prob > chi2	=	0.3416
Pseudo R2	=	0.0416
Breakdown point	=	50
M-estimate: k	=	4.6850649
S-estimate: k	=	1.547645
Scale	=	1.4825501

ziron	Coef.	Robust Std. Err.	t	P>|t|	[95% Conf.	Interval]
atypicalexposure						
No	0	(empty)				
Yes	.6704768	.4574576	1.47	0.151	-.2540794	1.595033
log10dailydose	-.2779597	.7819512	-0.36	0.724	-1.858342	1.302423
_cons	.9826048	2.104062	0.47	0.643	-3.269863	5.235073

b

MM regression (95% efficiency)

Number of obs	=	43
Wald chi2(2)	=	1.78
Prob > chi2	=	0.4113
Pseudo R2	=	0.0399
Breakdown point	=	50
M-estimate: k	=	4.6850649
S-estimate: k	=	1.547645
Scale	=	1.4302932

ziron	Coef.	Robust Std. Err.	t	P>|t|	[95% Conf.	Interval]
atypicalexposure						
No	0	(empty)				
Yes	.6473229	.4856012	1.33	0.190	-.3341138	1.62876
log10lifetime	-.100338	.3511701	-0.29	0.777	-.8100791	.6094032
_cons	.9023798	2.392903	0.38	0.708	-3.933857	5.738616

Table S15

Predicting iron content by type of antipsychotics used.

Robust regression estimates predicting PFC iron content (ziron, control-derived z-scores) by type of antipsychotics used (i.e. whether antipsychotic treatment had also included atypical agents [atypicalexposure, dichotomous variable]), controlling for (**a**) mean antipsychotic dose (dailydose, $\log_{10}$-chlorpromazine equivalent, mg/day) or (**b**) cumulative antipsychotic exposure (loglifetimedose, $\log_{10}$[chlorpromazine equivalent, mg/day) × (time, days]). Regression coefficients, robust standard errors, t-statistics along with their corresponding significance and 95%CI are presented, based on a linear regression model using iteratively reweighted least squares MM-estimators, derived using Stata's "robreg mm" command (95% efficiency, default parameters)[24]. n_{Scz}=43.

```
MM regression (95% efficiency)                  Number of obs     =         63
                                                Wald chi2(1)      =       0.00
                                                Prob > chi2       =     1.0000
                                                Pseudo R2         =     0.0105
                                                Breakdown point   =         50
                                                M-estimate: k     =  4.6850649
                                                S-estimate: k     =   1.547645
                                                Scale             =  1.2660709
```

ziron	Coef.	Robust Std. Err.	t	P>|t|	[95% Conf.	Interval]
toxap						
Negative	0	(empty)				
Positive	-.2934095	.3582204	-0.82	0.416	-1.009715	.4228965
_cons	.6373863	.2300192	2.77	0.007	.1774345	1.097338

Table S16

Predicting iron content by results of a postmortem toxicology essay for antipsychotics.

Robust regression estimates predicting PFC iron content (ziron, control-derived z-scores) based on a postmortem toxicology essay for antipsychotics (toxap). Regression coefficients, robust standard errors, t-statistics along with their corresponding significance and 95%CI are presented, based on a linear regression model using iteratively reweighted least squares MM-estimators, derived using Stata's "robreg mm" command (95% efficiency, default parameters)[24]. n_{Scz}=63.

Robust Statistics Number of obs = 133

0: dx = Controls
1: dx = Scz

zcopper	Coef.	Std. Err.	[95% Conf.	Interval]
0				
skewness	1.222157	.2503772	.7268862	1.717428
kurtosis	5.997486	.8157118	4.383928	7.611045
SK25	.2510199	.0761342	.100419	.4016208
QW25	.9438811	.0943264	.7572942	1.130468
MC	.2313643	.0642697	.1042325	.3584961
LMC	.0867667	.1051813	-.1212923	.2948257
RMC	-.0135731	.1055281	-.222318	.1951718
1				
skewness	.7146516	.1321791	.4531882	.9761151
kurtosis	3.934199	.3044865	3.331895	4.536504
SK25	.0079326	.0828576	-.155968	.1718333
QW25	1.047013	.0980925	.8529767	1.24105
MC	.0957511	.0774527	-.057458	.2489602
LMC	-.1318101	.1180784	-.3653808	.1017606
RMC	.3493572	.1086718	.1343938	.5643207

Normality Tests

	chi2	df	Prob>chi2
0			
JB	41.14	2	0.0000
MOORS	3.98	2	0.1366
MC-LR	4.17	3	0.2440
1			
JB	8.14	2	0.0171
MOORS	0.72	2	0.6988
MC-LR	4.74	3	0.1922

Table S17

Robust normality statistics for copper distribution in the Combined Cohort, grouped by diagnosis.

Generalized Jarque-Bera tests for normality, as suggested by Brys et al.[21], were derived using ROBSTAT, a Stata module to compute robust univariate statistics[12]. **JB**, classic Jarque-Bera normality test based on skewness and kurtosis[22]; SK25, Yule and Kendall skewness measure; QW25, quantile tail weight measure, at 25% (default); MC, medcouple tail weight measure; LMC, left medcouple tail weight measure; RMC, right medcouple tail weight measure. **MOORS**, robust skewness and tail-weight normality test based on SK25 and QW25[23]. **MC-LR**, robust skewness and tail-weight normality test based on MC, LMC and RMC[21]. $n_{controls}$=66, n_{Scz}=67.

a

MM regression (95% efficiency)

Number of obs	=	133
Wald chi2(1)	=	0.21
Prob > chi2	=	0.6500
Pseudo R2	=	0.0012
Breakdown point	=	50
M-estimate: k	=	4.6850649
S-estimate: k	=	1.547645
Scale	=	1.0049108

zcopper	Coef.	Robust Std. Err.	t	P>|t|	[95% Conf.	Interval]
dx	.0822517	.1812912	0.45	0.651	-.2763857	.440889
_cons	-.0854681	.116674	-0.73	0.465	-.3162771	.145341

b

Adjusted predictions Number of obs = 133
Model VCE : Robust

Expression : Fitted values, predict()

	Margin	Delta-method Std. Err.	z	P>|z|	[95% Conf.	Interval]
dx						
Controls	-.0854681	.116674	-0.73	0.464	-.314145	.1432088
Scz	-.0032164	.1493806	-0.02	0.983	-.2959971	.2895642

Table S18

The effect of diagnosis on PFC copper levels in the Combined Cohort, related to Figure S7.

(a) The effect of diagnosis on copper. Robust regression estimates predicting PFC copper levels (zcopper, control-derived z-scores) by diagnosis. Regression coefficients, robust standard errors, t-statistics along with their corresponding significance and 95%CI are presented, based on a linear regression model using iteratively reweighted least squares MM-estimators, derived using Stata's "robreg mm" command (95% efficiency, default parameters)[24]. (b) Robust marginal means, std. errors, t-statistic and 95%CIs for copper in each diagnostic group, based on above regression and robust variance-covariance estimates. $n_{controls}$=66, n_{Scz}=67.

a

Summary for variables: zcopper
by categories of: dx (Diagnosis)

dx	sd	variance
Controls	1	1
Scz	1.281519	1.64229

b

Diagnosis	Summary of zcopper Mean	Std. Dev.	Freq.
Controls	2.568e-09	1	66
Scz	.13292536	1.2815186	67
Total	.0669624	1.1480504	133

W0 = 2.1872994 df(1, 131) Pr > F = 0.14155371

W50 = 2.2856626 df(1, 131) Pr > F = 0.132983

W10 = 2.1785766 df(1, 131) Pr > F = 0.14234351

Table S19

Robust measures of copper distribution in the Combined Cohort.

(a) Standard deviation and variance of PFC copper among diagnostic groups. (b) Robust tests of the hypothesis that the variance of zcopper is the same across groups, including Levene's statistic (W0) and two statistics proposed by Brown and Forsythe that replace the mean in Levene's formula with alternative location estimators. The first alternative (W50) replaces the mean with the median. The second alternative replaces the mean with the 10 percent trimmed mean (W10). Note that the difference between groups was small and statistically insignificant in all analyses. $n_{controls}$=66, n_{Scz}=67.

a

MM regression (95% efficiency)

Number of obs	=	133
Wald chi2(3)	=	11.41
Prob > chi2	=	0.0097
Pseudo R2	=	0.1206
Breakdown point	=	50
M-estimate: k	=	4.6850649
S-estimate: k	=	1.547645
Scale	=	1.0356475

ziron	Coef.	Robust Std. Err.	t	P>|t|	[95% Conf.	Interval]
dx						
Controls	0	(empty)				
Scz	.5295934	.1986032	2.67	0.009	.1366522	.9225347
zcopper	.3434397	.173871	1.98	0.050	-.0005684	.6874478
dx#c.zcopper						
Controls	0	(empty)				
Scz	-.0916137	.1988682	-0.46	0.646	-.4850793	.3018518
_cons	-.0684018	.1106103	-0.62	0.537	-.2872471	.1504434

b

Average marginal effects | Number of obs = 133
Model VCE : Robust

Expression : Fitted values, predict()
dy/dx w.r.t. : zcopper

1._at : dx = 0

2._at : dx = 1

	dy/dx	Delta-method Std. Err.	z	P>|z|	[95% Conf.	Interval]
zcopper						
_at						
1	.3434397	.173871	1.98	0.048	.0026587	.6842206
2	.2518259	.0978376	2.57	0.010	.0600678	.4435841

Table S20

Predicting iron content by copper, controlling for diagnosis and copper × diagnosis interaction term, related to Figure S7b.

Robust regression estimates predicting PFC iron content (ziron, control-derived z-scores) based on copper (zcopper, control-derived z-scores), diagnosis (dx) and copper × diagnosis interaction term. Regression coefficients, robust standard errors, t-statistics along with their corresponding significance and 95%CI are presented, , based on a linear regression model using iteratively reweighted least squares MM-estimators, derived using Stata's "robreg mm" command (95% efficiency, default parameters)[24]. The effect of schizophrenia on iron at mean copper is highlighted. (b) Based on the regression model above, average marginal effects of copper on iron are depicted according to diagnosis. $n_{controls}$=66, n_{Scz}=67.

Robust Statistics Number of obs = 171

0: dx = Controls
1: dx = Scz

zzinc	Coef.	Std. Err.	[95% Conf.	Interval]
**0**				
skewness	1.336388	.374928	.5962743	2.076503
kurtosis	8.878379	1.173791	6.561296	11.19546
SK25	.111111	.0740726	-.0351095	.2573315
QW25	1.397191	.1205773	1.159169	1.635213
MC	.1110089	.0619684	-.0113178	.2333355
LMC	.0787863	.0883613	-.0956405	.253213
RMC	.1066588	.0920136	-.0749776	.2882951
**1**				
skewness	1.260537	.3081372	.6522691	1.868805
kurtosis	7.417602	1.060325	5.324503	9.510701
SK25	.069536	.0793964	-.0871938	.2262659
QW25	1.574624	.134839	1.30845	1.840798
MC	-1.62e-08	.0693534	-.1369047	.1369047
LMC	.0747068	.0851397	-.0933604	.242774
RMC	.2334512	.0854555	.0647606	.4021418

Normality Tests

	chi2	df	Prob>chi2
**0**			
JB	147.68	2	0.0000
MOORS	1.33	2	0.5150
MC-LR	1.70	3	0.6370
**1**			
JB	92.70	2	0.0000
MOORS	3.48	2	0.1756
MC-LR	0.57	3	0.9027

Table S21

Robust normality statistics for zinc distribution in the Combined Cohort, grouped by diagnosis.

Generalized Jarque-Bera tests for normality, as suggested by Brys et al.[21], were derived using ROBSTAT, a Stata module to compute robust univariate statistics[12]. **JB**, classic Jarque-Bera normality test based on skewness and kurtosis[22]; SK25, Yule and Kendall skewness measure; QW25, quantile tail weight measure, at 25% (default); MC, medcouple tail weight measure; LMC, left medcouple tail weight measure; RMC, right medcouple tail weight measure. **MOORS**, robust skewness and tail-weight normality test based on SK25 and QW25[23]. **MC-LR**, robust skewness and tail-weight normality test based on MC, LMC and RMC[21]. $n_{controls}$=85, n_{Scz}=86.

a

MM regression (95% efficiency)

Number of obs	=	171
Wald chi2(1)	=	0.00
Prob > chi2	=	1.0000
Pseudo R2	=	0.0222
Breakdown point	=	50
M-estimate: k	=	4.6850649
S-estimate: k	=	1.547645
Scale	=	.77424803

zzinc	Coef.	Robust Std. Err.	t	P>|t|	[95% Conf.	Interval]
dx						
Controls	0	(empty)				
Scz	.2846932	.1291864	2.20	0.029	.0296662	.5397201
_cons	-.0663504	.0910076	-0.73	0.467	-.2460086	.1133078

b

	Margin	Delta-method Std. Err.	z	P>|z|	[95% Conf.	Interval]
dx						
Controls	-.0663504	.0910076	-0.73	0.466	-.2447221	.1120213
Scz	.2183427	.0932274	2.34	0.019	.0356203	.4010652

Table S22

The effect of diagnosis on PFC zinc levels in the Combined Cohort, related to Figure S8.

(a) The effect of diagnosis on zinc. Robust regression estimates predicting PFC zinc levels (zzinc, control-derived z-scores) by diagnosis. Regression coefficients, robust standard errors, t-statistics along with their corresponding significance and 95%CI are presented, based on a linear regression model using iteratively reweighted least squares MM-estimators, derived using Stata's "robreg mm" command (95% efficiency, default parameters)[24]. (b) Robust marginal means, std. errors, t-statistic and 95%CIs for zinc in each diagnostic group, based on above regression and robust variance-covariance estimates.

Diagnosis	Summary of zzinc Mean	Std. Dev.	Freq.
Controls	2.695e-09	1	85
Scz	.30104325	.98458865	86
Total	.15140187	1.0008068	171

```
W0  =  0.00010517   df(1, 169)     Pr > F = 0.99182982

W50 =  0.00011568   df(1, 169)     Pr > F = 0.99143139

W10 =  0.00003390   df(1, 169)     Pr > F = 0.99536114
```

Table S23

Robust measures of zinc distribution in the Combined Cohort.

Robust tests of the hypothesis that the variance of PFC zinc (zzinc) is the same across groups, including Levene's statistic (W0) and two statistics proposed by Brown and Forsythe that replace the mean in Levene's formula with alternative location estimators. The first alternative (W50) replaces the mean with the median. The second alternative replaces the mean with the 10 percent trimmed mean (W10). No difference between groups was evident. $n_{controls}$=85, n_{Scz}=86.

a

MM regression (95% efficiency)

Number of obs	=	171
Wald chi2(3)	=	8.07
Prob > chi2	=	0.0446
Pseudo R2	=	0.0662
Breakdown point	=	50
M-estimate: k	=	4.6850649
S-estimate: k	=	1.547645
Scale	=	.7760008

zzinc	Coef.	Robust Std. Err.	t	P>|t|	[95% Conf.	Interval]
dx						
Controls	0	(empty)				
Scz	.1832276	.1363553	1.34375	0.1809	-.0859747	.4524299
ziron	.1976373	.0790904	2.49888	0.0134	.0414915	.3537831
dx#c.ziron						
Controls	0	(empty)				
Scz	-.0561578	.1082656	-.518704	0.6047	-.2699035	.1575879
_cons	-.0649933	.0889676	-.730528	0.4661	-.2406393	.1106528

b

Average marginal effects
Model VCE : Robust

Number of obs = 171

Expression : Fitted values, predict()
dy/dx w.r.t. : ziron

	dy/dx	Delta-method Std. Err.	z	P>|z|	[95% Conf.	Interval]
ziron						
dx						
Controls	.1976373	.0790904	2.50	0.012	.0426231	.3526516
Scz	.1414795	.075535	1.87	0.061	-.0065664	.2895255

Table S24

Predicting zinc content by iron, controlling for diagnosis, related to Figure S8b.

(a) Robust regression estimates predicting PFC zinc content (zzinc, control-derived z-scores) based on iron (ziron, control-derived z-scores), diagnosis (dx) and iron × diagnosis interaction term. Regression coefficients, robust standard errors, t-statistics along with their corresponding significance and 95%CI are presented, , based on a linear regression model using iteratively reweighted least squares MM-estimators, derived using Stata's "robreg mm" command (95% efficiency, default parameters)[24]. (b) Robust marginal means, std. errors, t-statistic and 95%CIs for iron in each diagnostic group, based on above regression and robust variance-covariance estimates. $n_{controls}$=85, n_{Scz}=86.

a

Generalized structural equation model   Number of obs = 170

Response : rzinc
Family : Gaussian
Link : identity

Response : dx
Family : Bernoulli
Link : logit

Log likelihood = -323.08823

	Coef.	Std. Err.	z	P>|z|	[95% Conf.	Interval]
**rzinc**						
riron	.1719068	.0502076	3.42	0.001	.0735018	.2703118
_cons	.1202317	.0702603	1.71	0.087	-.017476	.2579394
**dx**						
riron	.4548772	.1454578	3.13	0.002	.1697852	.7399693
rzinc	.4129499	.203323	2.03	0.042	.0144442	.8114556
_cons	-.2581428	.1740504	-1.48	0.138	-.5992754	.0829898
var(e.rzinc)	.7368439	.0799219			.5957304	.9113837

b

Bootstrap results   Number of obs = 170
Replications = 200

command: boot1 estat boot, bc percentile
_bs_1: r(iz_direct)
_bs_2: r(iz_indirect)
_bs_3: r(iz_total)
_bs_4: r(iz_directtotoal)
_bs_5: r(iz_indirecttotoal)

	Observed Coef.	Bootstrap Std. Err.	z	P>|z|	Normal-based [95% Conf.	Interval]
_bs_1	.4548772	.1413562	3.22	0.001	.1778242	.7319303
_bs_2	.0709889	.0387449	1.83	0.067	-.0049497	.1469275
_bs_3	.5258661	.1469085	3.58	0.000	.2379308	.8138015
_bs_4	.8650058	.0813335	10.64	0.000	.7055951	1.024416
_bs_5	.1349942	.0813335	1.66	0.097	-.0244164	.2944049

Table S25

Exploring the effect of iron on diagnosis through zinc, related to Figure S9.

(a) Generalized structural equation model with iron and zinc as continuous variables (z-scores adjusted for the covariates sex, age, race, pH and log_2PMI) and diagnosis as a binomial variable.
(b) Direct effect of iron on diagnosis, indirect effect of iron on diagnosis (via zinc), total effect of iron on diagnosis and the ratio of direct and indirect effect to the total effect were estimated using bootstrap-generated confidence intervals. The highly significant direct effect of iron on diagnosis was over five times larger than the non-significant indirect effect of iron on diagnosis, indicating that from a statistical viewpoint, the risk for a schizophrenia diagnosis conferred by elevated iron was unlikely to be mediated via zinc. N=170.

Robust Statistics Number of obs = 171

0: dx = Controls
1: dx = Scz

zfer	Coef.	Std. Err.	[95% Conf.	Interval]
0				
skewness	.1428266	.1056559	-.06574	.3513931
kurtosis	2.628252	.1495396	2.333059	2.923446
SK25	.032758	.0722402	-.1098454	.1753613
QW25	.9392639	.0844935	.7724724	1.106055
MC	.0063667	.0641266	-.1202204	.1329537
LMC	.1737927	.0975353	-.0187435	.366329
RMC	-.0953761	.0997599	-.2923038	.1015516
1				
skewness	1.333225	.1166087	1.103038	1.563413
kurtosis	5.568402	.5006535	4.580103	6.5567
SK25	-.0957544	.0778973	-.2495249	.0580161
QW25	1.537868	.1432419	1.255106	1.820629
MC	.0320769	.0674763	-.1011225	.1652762
LMC	.2302789	.0915118	.049633	.4109248
RMC	.5342992	.0782846	.379764	.6888344

Normality Tests

	chi2	df	Prob>chi2
0			
JB	0.78	2	0.6776
MOORS	2.34	2	0.3107
MC-LR	2.89	3	0.4095
1			
JB	49.12	2	0.0000
MOORS	3.02	2	0.2204
MC-LR	4.08	3	0.2530

Table S26

Robust normality statistics for ferritin distribution in the Combined Cohort, grouped by diagnosis.

Generalized Jarque-Bera tests for normality (right), as suggested by Brys et al.[21], were derived using ROBSTAT, a Stata module to compute robust univariate statistics[12]. **JB**, classic Jarque-Bera normality test based on skewness and kurtosis[22]; SK25, Yule and Kendall skewness measure; QW25, quantile tail weight measure, at 25% (default); MC, medcouple tail weight measure; LMC, left medcouple tail weight measure; RMC, right medcouple tail weight measure. **MOORS**, robust skewness and tail-weight normality test based on SK25 and QW25[23]. **MC-LR**, robust skewness and tail-weight normality test based on MC, LMC and RMC[21]. Based on the robust versions, ferritin was normally distributed in both groups. $n_{controls}$=85, n_{Scz}=86.

a

```
MM regression (95% efficiency)                 Number of obs     =        171
                                               Wald chi2(1)      =       5.82
                                               Prob > chi2       =     0.0159
                                               Pseudo R2         =     0.0260
                                               Breakdown point   =         50
                                               M-estimate: k     =  4.6850649
                                               S-estimate: k     =   1.547645
                                               Scale             =  1.1878906
```

zfer	Coef.	Robust Std. Err.	t	P>|t|	[95% Conf.	Interval]
dx	-.4487999	.1860977	-2.41	0.017	-.8161756	-.0814243
_cons	-.0097303	.1123905	-0.09	0.931	-.2316004	.2121399

b

```
Adjusted predictions                           Number of obs     =        171
Model VCE    : Robust

Expression   : Fitted values, predict()
```

	Margin	Delta-method Std. Err.	z	P>|z|	[95% Conf.	Interval]
dx						
Controls	-.0097303	.1123905	-0.09	0.931	-.2300116	.2105511
Scz	-.4585302	.1493181	-3.07	0.002	-.7511882	-.1658722

Table S27

Robust between-group comparison of PFC ferritin levels in the Combined Cohort, visualized in Figures 1b and S10b.

(a) The effect of diagnosis on ferritin. Robust regression estimates predicting PFC ferritin (fer, control-derived z-scores) by diagnosis. Regression coefficients, robust standard errors, t-statistics along with their corresponding significance and 95%CI are presented, based on an iteratively reweighted least squares MM-estimators regression model, derived using Stata's "robreg mm" command (efficiency 95% and default parameters)[24]. (b) Robust marginal means, std. errors, t-statistic and 95%CI for ferritin in each diagnostic group, based on above robust regression and robust variance-covariance estimates. $n_{controls}$=85, n_{Scz}=86.

```
MM regression (95% efficiency)                  Number of obs     =        171
                                                Wald chi2(6)      =       9.61
                                                Prob > chi2       =     0.1422
                                                Pseudo R2         =     0.0608
                                                Breakdown point   =         50
                                                M-estimate: k     =  4.6850649
                                                S-estimate: k     =   1.547645
                                                Scale             =  1.1514637
```

zfer	Coef.	Robust Std. Err.	t	P>|t|	[95% Conf.	Interval]
dx	-.4761295	.1795888	-2.65	0.009	-.8307337	-.1215253
age	.0025185	.0056853	0.44	0.658	-.0087073	.0137443
sex						
Female	0	(empty)				
Male	-.0992149	.2023327	-0.49	0.625	-.4987279	.3002981
race01						
Caucasian	0	(empty)				
Non-Caucasian	.4436908	.2233614	1.99	0.049	.0026559	.8847256
ph	-.2154674	.375729	-0.57	0.567	-.9573572	.5264224
log2pmi	-.2251547	.1053352	-2.14	0.034	-.4331427	-.0171667
_cons	2.345594	2.487308	0.94	0.347	-2.565681	7.25687

Table S28

Robust between-group comparison of PFC ferritin levels in the Combined Cohort, controlling for covariates.

Robust regression estimates predicting PFC ferritin levels (zfer, control-derived z-scores) by diagnosis, controlling for age, sex, race, sample pH and post-mortem interval log-PMI (log_2-pmi, hours). For binary variables, coefficients relating to the value denoted beneath variable name (compared to the baseline level) are provided. Regression coefficients, robust standard errors, t-statistics along with their corresponding significance and 95%CI are presented, based on an iteratively reweighted least squares MM-estimators regression model, derived using Stata's "robreg mm" command (efficiency 95% and default parameters)[24]. $n_{controls}$=85, n_{Scz}=86.

a

```
MM regression (95% efficiency)                 Number of obs     =        171
                                               Wald chi2(1)      =       9.21
                                               Prob > chi2       =     0.0024
                                               Pseudo R2         =     0.0472
                                               Breakdown point   =         50
                                               M-estimate: k     =  4.6850649
                                               S-estimate: k     =   1.547645
                                               Scale             =  1.0317872
```

zirontofer	Coef.	Robust Std. Err.	t	P>|t|	[95% Conf.	Interval]
dx	.6190859	.204032	3.03	0.003	.2163063	1.021865
_cons	-.0822745	.1097139	-0.75	0.454	-.2988608	.1343117

b

```
Adjusted predictions                           Number of obs     =        171
Model VCE      : Robust

Expression     : Fitted values, predict()
```

	Margin	Delta-method Std. Err.	z	P>|z|	[95% Conf.	Interval]
dx						
Controls	-.0822745	.1097139	-0.75	0.453	-.2973098	.1327607
Scz	.5368113	.1902453	2.82	0.005	.1639375	.9096852

Table S29

Robust between-group comparison of PFC iron-to-ferritin ratio in the Combined Cohort, visualized in Figures 1c and S10c.

(a) The effect of diagnosis on iron-to-ferritin ratio. Robust regression estimates predicting PFC iron-to-ferritin ratio (zirontofer, control-derived z-scores) by diagnosis. Regression coefficients, robust standard errors, t-statistics along with their corresponding significance and 95%CI are presented, based on an iteratively reweighted least squares MM-estimators regression model, derived using Stata's "robreg mm" command (efficiency 95% and default parameters)[24]. (b) Robust marginal means, std. errors, t-statistic and 95%CI for iron-to-ferritin ratio in each diagnostic group, based on above robust regression and robust variance-covariance estimates. $n_{controls}$=85, n_{Scz}=86.

a

Akaike's information criterion and Bayesian information criterion

Model	N	ll(null)	ll(model)	df	AIC	BIC
Log	85	-120.1068	-117.9891	2	239.9782	244.8635
SQRT	85	-120.1068	-118.2135	2	240.4269	245.3122
Linear	85	-120.1068	-118.4643	2	240.9286	245.8139
Quadratic	85	-120.1068	-118.9454	2	241.8909	246.7762
Cubic	85	-120.1068	-119.3197	2	242.6394	247.5247

b

MM regression (95% efficiency)

Number of obs	=	85
Wald chi2(1)	=	5.72
Prob > chi2	=	0.0168
Pseudo R2	=	0.0438
Breakdown point	=	50
M-estimate: k	=	4.6850649
S-estimate: k	=	1.547645
Scale	=	.94467502

ziron	Coef.	Robust Std. Err.	t	P>|t|	[95% Conf.	Interval]
lnage	.638109	.2667877	2.39	0.019	.107479	1.168739
_cons	-2.581698	1.060207	-2.44	0.017	-4.690406	-.4729893

Table S30

Robust analysis of the relationship between PFC iron and age among control individuals, visualized in Figures 2a and S11b.

(a) Information criteria (IC) for five alternative linear regression models predicting PFC iron among control individuals based on either the logarithm (Log), the square-root (SQRT), the non-transformed (Linear), the 2nd power (Quadratic) or the 3rd power (Cubic) of age. While all models that included 'age' as a predictor displayed higher log-likelihood (ll) values compared to the null model, a Log model displayed the lowest IC among alternative models, providing a rationale for estimating the age-iron relationship using a linear-logarithmic model. (b) The effect of age on iron. Robust regression estimates predicting PFC iron (ziron, control-derived z-scores) by the natural logarithm of age (lnage). Regression coefficients, robust standard errors, t-statistics along with their corresponding significance and 95%CI are presented, based on an iteratively reweighted least squares MM-estimators regression model, derived using Stata's "robreg mm" command (efficiency 95% and default parameters)[24]. $n_{controls}$=85.

a

Akaike's information criterion and Bayesian information criterion

Model	N	ll(null)	ll(model)	df	AIC	BIC
Log	86	-157.1576	-157.0676	2	318.1352	323.0439
SQRT	86	-157.1576	-157.0761	2	318.1522	323.0609
Linear	86	-157.1576	-157.0886	2	318.1772	323.0859
Quadratic	86	-157.1576	-157.119	2	318.238	323.1467
Cubic	86	-157.1576	-157.1438	2	318.2875	323.1962

b

MM regression (95% efficiency)

Number of obs	=	86
Wald chi2(1)	=	0.07
Prob > chi2	=	0.7931
Pseudo R2	=	0.0008
Breakdown point	=	50
M-estimate: k	=	4.6850649
S-estimate: k	=	1.547645
Scale	=	1.4218448

ziron	Coef.	Robust Std. Err.	t	P>|t|	[95% Conf.	Interval]
age	.0029434	.0112221	0.26	0.794	-.019373	.0252599
_cons	.4520327	.5991902	0.75	0.453	-.7395228	1.643588

Table S31

Robust analysis of the relationship between PFC iron and age among schizophrenia cases, visualized in Figures 2a and S11b.

(a) Information criteria (IC) for five alternative linear regression models predicting PFC iron among schizophrenia cases based on either the logarithm (Log), the square-root (SQRT), the non-transformed (Linear), the 2nd power (Quadratic) or the 3rd power (Cubic) of age. As none of the models that included 'age' as a predictor displayed log-likelihood (ll) values that were different than the null model, and IC were very similar across models, the simplest (i.e., linear) model was selected. (b) The effect of age on iron. Robust regression estimates predicting PFC iron (ziron, control-derived z-scores) by age. Regression coefficients, robust standard errors, t-statistics along with their corresponding significance and 95%CI are presented, based on an iteratively reweighted least squares MM-estimators regression model, derived using Stata’s “robreg mm” command (efficiency 95% and default parameters)[24]. n_{Scz}=86.

age<	n	OR	p-beta	-log10p-beta
25	11	2.14	0.219	0.659556
27	12	2.36	0.2	0.69897
29	15	2.64	0.135	0.869666
31	18	2.56	0.096	1.017729
33	19	2.62	0.097	1.013228
**35**	**26**	**3.72**	**0.027**	**1.568636**
37	28	1.96	0.061	1.21467
39	32	1.9	0.047	1.327902
41	33	1.8	0.061	1.21467
43	38	1.65	0.057	1.244125
45	44	1.58	0.066	1.180456
47	48	1.65	0.038	1.420216
49	52	1.54	0.059	1.229148
51	59	1.53	0.048	1.318759
53	67	1.51	0.04	1.39794
55	77	1.51	0.041	1.387216
57	92	1.62	0.008	2.09691
59	107	1.64	0.003	2.522879
61	118	1.63	0.0017	2.769551
63	126	1.65	0.001	3
65	131	1.69	0.0006	3.221849
67	141	1.76	0.0002	3.69897
69	145	1.7	0.0003	3.522879
71	146	1.72	0.0002	3.69897
73	150	1.63	0.0004	3.39794
75	157	1.63	0.0004	3.39794
77	161	1.61	0.0004	3.39794
79	164	1.61	0.0004	3.39794
81	164	1.61	0.0004	3.39794
83	166	1.61	0.0003	3.522879
85	169	1.59	0.0004	3.39794
87	170	1.6	0.0003	3.522879

Table S32

Logistic regression analyses predicting disease status based on PFC iron for sequential age cutoffs, visualized in Figure 2b.

Each row represents one of a series of logistic regression analyses predicting disease status based on PFC iron, after iron had been adjusted for relevant covariates (sex, ethnicity, sample pH and post-mortem interval). Beginning at age 31, for each analysis, age cutoff (years), number of

individuals (n) who had died younger than the designated age cutoff and were thus entered into the analysis, odds ratio (OR) of having been diagnosed with of schizophrenia (as compared to being a control) attributed to a 1 SD increase in covariate-adjusted iron, significance of the beta-coefficient for iron as a predictor in the model (p-beta) and its negative $\log_{10}$-transformed value (-log10p-beta) are presented. As the odds ratio was maximal when the analysis included only individuals who had died younger than 35, this age (bolded) was selected as a cutoff for generating subcohorts to be contrasted in subsequent panels. $N_{whole\ sample}$=170 (see also Figure S11).

a

Logistic regression		Number of obs	=	26
		LR chi2(1)	=	9.45
		Prob > chi2	=	0.0021
Log likelihood = -12.045177		Pseudo R2	=	0.2818

dx	Odds Ratio	Std. Err.	z	P>|z|	[95% Conf.	Interval]
zriron	3.719361	2.210866	2.21	0.027	1.160102	11.92451
_cons	1.929725	1.016142	1.25	0.212	.6875041	5.41646

Note: _cons estimates baseline odds.

b

Logistic regression		Number of obs	=	144
		LR chi2(1)	=	9.50
		Prob > chi2	=	0.0021
Log likelihood = -94.93655		Pseudo R2	=	0.0477

dx	Odds Ratio	Std. Err.	z	P>|z|	[95% Conf.	Interval]
zriron	1.489974	.207099	2.87	0.004	1.13466	1.956552
_cons	.7846348	.1406998	-1.35	0.176	.552117	1.115075

Note: _cons estimates baseline odds.

Table S33

Logistic regression analyses predicting disease status based on PFC iron in young and old subcohorts, visualized in Figure 2c.

Logistic regression analyses predicting disease status based on PFC iron (zriron), after iron had been adjusted for relevant covariates (sex, ethnicity, sample pH and post-mortem interval), among individuals who had died (a) younger or (b) older than 35. Odds ratios, standard errors, z-statistics along with their corresponding significance and 95%CIs are presented. $n_{age<35}$=26, $n_{age\geq35/con}$=144.

a

MM regression (95% efficiency)

Number of obs	=	26
Wald chi2(1)	=	10.43
Prob > chi2	=	0.0012
Pseudo R2	=	0.2264
Breakdown point	=	50
M-estimate: k	=	4.6850649
S-estimate: k	=	1.547645
Scale	=	1.3167348

zriron1	Coef.	Robust Std. Err.	t	P>|t|	[95% Conf.	Interval]
dx	1.530622	.4738344	3.23	0.004	.5526756	2.508568
_cons	.3297846	.2164106	1.52	0.141	-.1168649	.776434

b

MM regression (95% efficiency)

Number of obs	=	144
Wald chi2(1)	=	3.58
Prob > chi2	=	0.0585
Pseudo R2	=	0.0252
Breakdown point	=	50
M-estimate: k	=	4.6850649
S-estimate: k	=	1.547645
Scale	=	1.1966403

zriron1	Coef.	Robust Std. Err.	t	P>|t|	[95% Conf.	Interval]
dx	.4580092	.2420547	1.89	0.061	-.0204872	.9365057
_cons	1.063518	.1202748	8.84	0.000	.8257574	1.301278

Table S34

Robust between-group comparison of PFC iron levels in the young and old subcohorts, visualized in Figure 2d.

The effect of diagnosis on iron in age subcohorts. Robust regression estimates predicting PFC iron (zriron, after iron had been adjusted for relevant covariates [sex, ethnicity, sample pH and post-mortem interval] and then normalised to control distribution and control values were mean-centred at 1) by diagnosis. For both the (a) young (age<35) and (b) old (age≥35) subcohorts, regression coefficients, robust standard errors, t-statistics along with their corresponding significance and 95%CI are presented, based on an iteratively reweighted least squares MM-estimators regression model, derived using Stata's "robreg mm" command (95% efficiency, default parameters)[24]. $n_{age<35/con}$=9, $n_{age<35/Scz}$=17; $n_{age\geq35/con}$ =76, $n_{age\geq35/Scz}$=69.

a

Akaike's information criterion and Bayesian information criterion

Model	N	ll(null)	ll(model)	df	AIC	BIC
Log	85	-120.1068	-117.6717	2	239.3434	244.2287
SQRT	85	-120.1068	-118.1638	2	240.3276	245.2129
Linear	85	-120.1068	-118.6837	2	241.3674	246.2527
Quadratic	85	-120.1068	-119.2775	2	242.555	247.4403
Cubic	85	-120.1068	-119.5282	2	243.0564	247.9417

b

MM regression (95% efficiency)

Number of obs	=	85
Wald chi2(1)	=	7.05
Prob > chi2	=	0.0079
Pseudo R2	=	0.0564
Breakdown point	=	50
M-estimate: k	=	4.6850649
S-estimate: k	=	1.547645
Scale	=	1.07575

zfer	Coef.	Robust Std. Err.	t	P>|t|	[95% Conf.	Interval]
lnziron2	.3631582	.1368195	2.65	0.010	.0910297	.6352867
_cons	-.1937498	.1153374	-1.68	0.097	-.4231513	.0356517

Table S35

Robust analysis of the relationship between PFC ferritin and iron among control individuals, visualized in Figures 3a and S12a.

(a) Information criteria (IC) for five alternative linear regression models predicting PFC ferritin among control individuals based on either the logarithm (Log), the square-root (SQRT), the non-transformed (Linear), the 2nd power (Quadratic) or the 3rd power (Cubic) of iron (z-scores mean-centred at 2). While all models that included iron as a predictor displayed higher log-likelihood (ll) values compared to the null model, a Log model displayed the lowest IC among alternative models, providing a rationale for estimating the iron-ferritin relationship using a linear-logarithmic model. (b) The effect of iron on ferritin. Robust regression estimates predicting PFC ferritin (zfer, control-derived z-scores) by the natural logarithm of iron (lzniron2, ln[z-scores+2]). Regression coefficients, robust standard errors, t-statistics along with their corresponding significance and 95%CI are presented, based on an iteratively reweighted least squares MM-estimators regression model, derived using Stata's "robreg mm" command (efficiency 95% and default parameters)[24]. $n_{controls}$=85.

a

Model	N	ll(null)	ll(model)	df	AIC	BIC
Log	86	-172.0171	-170.727	2	345.4541	350.3628
SQRT	86	-172.0171	-171.0997	2	346.1993	351.108
Linear	86	-172.0171	-171.3436	2	346.6871	351.5958
Quadratic	86	-172.0171	-171.5007	2	347.0014	351.9101
Cubic	86	-172.0171	-171.4291	2	346.8581	351.7668

b

MM regression (95% efficiency)

Number of obs	=	86
Wald chi2(1)	=	7.52
Prob > chi2	=	0.0061
Pseudo R2	=	0.0597
Breakdown point	=	50
M-estimate: k	=	4.6850649
S-estimate: k	=	1.547645
Scale	=	1.3201531

zfer	Coef.	Robust Std. Err.	t	P>|t|	[95% Conf.	Interval]
lnziron2	.6518512	.2376912	2.74	0.007	.1791761	1.124526
_cons	-.9750405	.2115329	-4.61	0.000	-1.395697	-.5543841

Table S36

Robust analysis of the relationship between PFC ferritin and iron among schizophrenia cases, visualized in Figures 3a and S12a.

(a) Information criteria (IC) for five alternative linear regression models predicting PFC ferritin among schizophrenia cases based on either the logarithm (Log), the square-root (SQRT), the non-transformed (Linear), the 2nd power (Quadratic) or the 3rd power (Cubic) of iron (z-scores mean-centred at 2). While all models that included iron as a predictor displayed higher log-likelihood (ll) values compared to the null model, a Log model displayed the lowest IC among alternative models, providing a rationale for estimating the iron-ferritin relationship using a linear-logarithmic model. (b) The effect of iron on ferritin. Robust regression estimates predicting PFC ferritin (zfer, control-derived z-scores) by the natural logarithm of iron (lzniron2, ln[z-scores+2]). Regression coefficients, robust standard errors, t-statistics along with their corresponding significance and 95%CI are presented, based on an iteratively reweighted least squares MM-estimators regression model, derived using Stata's "robreg mm" command (efficiency 95% and default parameters)[24]. n_{Scz}=86.

a

MM regression (95% efficiency)			
	Number of obs	=	171
	Wald chi2(3)	=	21.40
	Prob > chi2	=	0.0001
	Pseudo R2	=	0.0824
	Breakdown point	=	50
	M-estimate: k	=	4.6850649
	S-estimate: k	=	1.547645
	Scale	=	1.1726643

zfer	Coef.	Robust Std. Err.	t	P>|t|	[95% Conf.	Interval]
lnziron2	.3605118	.1365412	2.64	0.009	.0909424	.6300812
dx						
Controls	0	(empty)				
Scz	-.7916501	.243711	-3.25	0.001	-1.272802	-.3104987
dx#c.lnziron2						
Controls	0	(empty)				
Scz	.2807686	.2762914	1.02	0.311	-.2647055	.8262427
_cons	-.1919033	.1148384	-1.67	0.097	-.4186255	.0348189

Table S37

Predicting ferritin content by iron, controlling for diagnosis and iron × diagnosis interaction term, visualized in Figure 3a.

(Robust regression estimates predicting PFC ferritin content (zfer, control-derived z-scores) based on the logarithm of iron (lzniron2, ln[z-scores+2]), diagnosis (dx) and lzniron2 × diagnosis interaction term. Regression coefficients, robust standard errors, t-statistics along with their corresponding significance and 95%CI are presented, based on an iteratively reweighted least squares MM-estimators regression model, derived using Stata's "robreg mm" command (efficiency 95% and default parameters)[24]. $n_{controls}$=85, n_{Scz}=86.

ziron<	n	OR	p-beta	-log10p-beta
-1	20	0.296913	0.0526	1.279014
-0.9	25	0.265176	0.0336	1.473661
-0.8	31	0.251903	0.0111	1.954677
-0.7	38	0.390341	0.0243	1.614394
-0.6	44	0.371927	0.015	1.823909
-0.5	46	0.364014	0.0127	1.896196
-0.4	49	0.334775	0.0074	2.130768
-0.3	53	0.303957	0.0033	2.481486
-0.2	62	0.283759	0.0015	2.823909
-0.1	68	0.288612	0.0007	3.154902
**0**	**69**	**0.283994**	**0.0005**	3.30103
0.1	80	0.515795	0.0074	2.130768
0.2	89	0.504238	0.0044	2.356547
0.3	93	0.599543	0.0184	1.735182
0.4	97	0.706049	0.075	1.124939
0.5	101	0.701461	0.061	1.21467
0.6	108	0.683682	0.039	1.408935
0.7	114	0.805725	0.183	0.737549
0.8	118	0.835142	0.256	0.59176
0.9	124	0.828896	0.23	0.638272
1	127	0.902324	0.464	0.333482

Table S38

Logistic regression analyses predicting disease status based on PFC ferritin for sequential iron cutoffs, visualized in Figure 2b.

Each row represents one of a series of logistic regression analyses predicting disease status based on PFC ferritin, after ferritin had been adjusted for relevant covariates (age, sex, ethnicity, sample pH and post-mortem interval). Beginning at -1 SDs, for each analysis, iron cutoff (z-score based on distribution of control individuals), number of individuals (n) who had iron levels below the designated iron cutoff and were thus entered into the analysis, odds ratio (OR) of having been diagnosed with of schizophrenia (as compared to being a control) attributed to a 1 SD increase in covariate-adjusted ferritin, significance of the beta-coefficient for ferritin as a predictor in the model (p-beta) and its negative $\log_{10}$-transformed value (-log10p-beta) are presented. As the odds ratio had a (regional) minimum when the analysis included only individuals who had iron levels below the control group's mean, the value 0 (bolded) was selected as a cutoff for generating subcohorts to be contrasted in subsequent panels. $N_{whole\ sample}$=169 (see also Figure S10).

a

Logistic regression

	Number of obs	=	69
	LR chi2(1)	=	17.88
	Prob > chi2	=	0.0000
Log likelihood = -36.772686	Pseudo R2	=	0.1955

dx	Odds Ratio	Std. Err.	z	P>|z|	[95% Conf.	Interval]
zrfer	.283994	.1030452	-3.46928	0.0005	.1394623	.578311
_cons	.28257	.1068833	-3.34122	0.0008	.1346342	.5930573

b

Logistic regression

	Number of obs	=	100
	LR chi2(1)	=	0.07
	Prob > chi2	=	0.7933
Log likelihood = -67.994865	Pseudo R2	=	0.0005

dx	Odds Ratio	Std. Err.	z	P>|z|	[95% Conf.	Interval]
zrfer	1.038162	.1487128	0.26	0.794	.7840321	1.374664
_cons	1.374492	.2795578	1.56	0.118	.9226065	2.047708

Table S39

Logistic regression analyses predicting disease status based on PFC ferritin in low-iron and high-iron subcohorts, visualized in Figure 3c.

Logistic regression analyses predicting disease status based on PFC ferritin (zfer), after ferritin had been adjusted for relevant covariates (age, sex, ethnicity, sample pH and post-mortem interval), among individuals who had PFC iron levels (a) below or (b) above the control group's mean. Odds ratios, standard errors, z-statistics along with their corresponding significance and 95%CIs are presented. $n_{iron<mean}$=69, $n_{iron\geq mean}$=100.

a

MM regression (95% efficiency)		
Number of obs	=	69
Wald chi2(1)	=	15.34
Prob > chi2	=	0.0001
Pseudo R2	=	0.1806
Breakdown point	=	50
M-estimate: k	=	4.6850649
S-estimate: k	=	1.547645
Scale	=	.97467704

zrfer2	Coef.	Robust Std. Err.	t	P>|t|	[95% Conf.	Interval]
dx	-.96069	.2452903	-3.91654	0.0002	-1.450292	-.4710885
_cons	1.879251	.1508747	12.4557	0.0000	1.578104	2.180399

b

MM regression (95% efficiency)		
Number of obs	=	100
Wald chi2(1)	=	0.45
Prob > chi2	=	0.5011
Pseudo R2	=	0.0035
Breakdown point	=	50
M-estimate: k	=	4.6850649
S-estimate: k	=	1.547645
Scale	=	1.2385585

zrfer2	Coef.	Robust Std. Err.	t	P>|t|	[95% Conf.	Interval]
dx	-.1695387	.2520135	-.672736	0.5027	-.6696513	.330574
_cons	2.085432	.1630607	12.7893	0.0000	1.761844	2.409021

Table S40

Robust between-group comparison of PFC ferritin in low-iron and high-iron subcohorts, visualized in Figure 3d.

The effect of diagnosis on ferritin in iron subcohorts. Robust regression estimates predicting PFC ferritin (zfer2, after ferritin had been adjusted for relevant covariates [age, sex, ethnicity, sample pH and post-mortem interval] and then normalised to control distribution and control values were mean-centred at 2) by diagnosis. For both the (a) low-iron (ziron<0) and (b) high-iron (ziron>0) subcohorts, regression coefficients, robust standard errors, t-statistics along with their corresponding significance and 95%CI are presented, based on an iteratively reweighted least squares M-estimators regression model, derived using Stata's "robreg m" command (efficiency 95%, default parameters)[24]. $n_{iron<mean/con}$=43, $n_{iron<mean/Scz}$=26; $n_{iron>mean/con}$=42, $n_{iron>mean/Scz}$=58.

a

```
Empirical cutpoint estimation
Method:                              Nearest to (0,1)
Reference variable:                  dx (0=neg, 1=pos)
Classification variable:             zriron
Empirical optimal cutpoint:          .99145541
Sensitivity at cutpoint:             0.71
Specificity at cutpoint:             0.89
```

b

```
Empirical cutpoint estimation
Method:                              Youden
Reference variable:                  rdx (0=neg, 1=pos)
Classification variable:             zrfer
Empirical optimal cutpoint:          1.1653351
Youden index (J):                    0.406
SE(J):                               0.1138
Sensitivity at cutpoint:             0.79
Specificity at cutpoint:             0.62
```

c

```
Empirical cutpoint estimation
Method:                              Youden
Reference variable:                  dx (0=neg, 1=pos)
Classification variable:             zr_zirontofer
Empirical optimal cutpoint:          .1521758
Youden index (J):                    0.342
SE(J):                               0.0727
Sensitivity at cutpoint:             0.66
Specificity at cutpoint:             0.68
```

Table S41

Deriving optimal cutpoints, visualized in Figure 4.

Optimal cutpoints were empirically derived from individual ROCs (Figure 4a) based on (a) iron in individuals younger than 35, on (b) ferritin in individuals with below-mean iron and on (c) the iron-to-ferritin ratio. Optimal cutoffs were derived using Youden method (except for iron which was based on near method since Youden provided a false-positive rate of 0). For each cutoff, high risk values and accuracy are depicted in Figure 4b. N=167.

Model **FullModel**

Logistic regression		Number of obs	=	167
		LR chi2(5)	=	35.45
		Prob > chi2	=	0.0000
Log likelihood = -98.005225		Pseudo R2	=	0.1531

dxx	Odds Ratio	Std. Err.	z	P>|z|	[95% Conf.	Interval]
dxiron						
no_data	.8055758	.6004085	-0.29	0.772	.1869357	3.471527
Scz	7.605292	9.801953	1.57	0.115	.6082178	95.09829
dxfer						
no_data	2.9083	1.453144	2.14	0.033	1.092277	7.743648
Scz	5.132431	3.063959	2.74	0.006	1.592842	16.53764
dxirontofer						
Scz	2.449582	.9301858	2.36	0.018	1.163759	5.156094
_cons	.2977764	.2093948	-1.72	0.085	.0750464	1.181547

Note: _cons estimates baseline odds.

Table S42

Logistic regression model predicting disease based on a combination of three predictors following definition of optimal cutpoints, visualized in Figure 4.

A logistic regression model that predicts disease based on a combination of three optimal cutpoints (iron in individuals younger than 35, ferritin in individuals with below-mean iron and iron-to-ferritin ratio). Based on these cutpoints, for each continuous predictor a categorical predictor denoting the high-risk category (Scz, Figure 4b) and a category for no data have been generated. N=167.

a

Model **NoIronModel**

Logistic regression	Number of obs	=	167
	LR chi2(3)	=	28.10
	Prob > chi2	=	0.0000
Log likelihood = -101.67993	Pseudo R2	=	0.1214

dxx	Odds Ratio	Std. Err.	z	P>|z|	[95% Conf.	Interval]
dxfer						
no_data	2.909269	1.335666	2.33	0.020	1.183018	7.154455
Scz	4.558505	2.680728	2.58	0.010	1.439643	14.43411
dxirontofer						
Scz	2.805834	1.035462	2.80	0.005	1.361243	5.78347
_cons	.2620356	.096133	-3.65	0.000	.1276673	.5378247

Note: _cons estimates baseline odds.

```
Likelihood-ratio test                                 LR chi2(2)  =      7.35
(Assumption: NoIronModel nested in FullModel)         Prob > chi2 =    0.0254
```

b

Model **NoFerModel**

Logistic regression	Number of obs	=	167
	LR chi2(3)	=	26.92
	Prob > chi2	=	0.0000
Log likelihood = -102.26726	Pseudo R2	=	0.1163

dxx	Odds Ratio	Std. Err.	z	P>|z|	[95% Conf.	Interval]
dxiron						
no_data	1.088708	.6964988	0.13	0.894	.3107112	3.814749
Scz	9.831514	12.05148	1.86	0.062	.8896441	108.6487
dxirontofer						
Scz	3.645883	1.229736	3.84	0.000	1.882336	7.061685
_cons	.4554583	.2845578	-1.26	0.208	.1338571	1.54973

Note: _cons estimates baseline odds.

```
Likelihood-ratio test                                 LR chi2(2)  =      8.52
(Assumption: NoFerModel nested in FullModel)          Prob > chi2 =    0.0141
```

Table S43

Likelihood-ratio tests comparing nested and full regression models, related to Figure 4.

Likelihood-ratio tests assessing whether the full three-predictor model (presented in Table S37) was superior to nested models based on two predictors only (iron-to-ferritin ratio and either [a] ferritin or [b] iron alone). N=167.

```
Empirical cutpoint estimation
Method:                              Youden
Reference variable:                  dxx (0=neg, 1=pos)
Classification variable:             xbi
Empirical optimal cutpoint:          .20796901
Youden index (J):                    0.414
SE(J):                               0.0704
Sensitivity at cutpoint:             0.69
Specificity at cutpoint:             0.72
Area under ROC curve at cutpoint:    0.71
```

Table S44

Deriving an optimal cutoff for a logistic regression model combining all three predictors, visualized in Figure 4.

Optimal cutoff (Youden method) was empirically derived with the classification variable (xbi) representing linear prediction from a logistic regression model predicting disease based on a combination of three predictors (Table S37). N=167.

Gene	Protein	SNP	Allele	Location	OR	*P*-value	RegulomeDB[a]	LINSIGHT[b]
TFRC	Transferrin receptor	rs74199047	G/C	intron	0.97	9.51e-02	3a	0.05
SLC11A2	Divalent metal transporter-1	rs17216107		intron	0.95	2.28e-01	5	0.96
TF	Transferrin	rs145651545	A/G	intron	1.15	6.17e-04	7	0.04
SLC40A1	Ferroportin	rs72916297	T/G	intron	1.04	7.74e-02	7	0.10
HAMP	Hepcidin antimicrobial peptide	rs55863037	G/A	intron	0.96	4.00e-03	5	0.07
CP	Ceruloplasmin	rs772904	T/G	intron	0.92	1.20e-03	6	0.05
FTL	Ferritin, light polypeptide	rs2230267	T/C	synonymous-coding	1.01	2.04e-01	4	-
FTH1	Ferritin, heavy polypeptide	rs76059597	T/C	intron	0.98	5.25e-01	4	0.07
FTMT	Ferritin, mitochondrial	rs142007771	C/A	missense	0.97	3.01e-01	5	-
ACO1	Aconitase-1	rs77465798	C/T	intron	0.91	2.20e-03	7	0.05
FBXL5	F-box/LRR-repeat protein 5	rs146796998	C/T	intron	0.92	1.49e-02	7	0.05
HIF1A	Hypoxia inducible factor 1, alpha subunit	rs79678604	G/C	intron	0.93	2.30e-03	5	0.05
EPAS1	Endothelial PAS domain protein 1 (hypoxia inducible factor 2, alpha subunit)	rs9973653	G/T	intron	1.02	2.20e-02	5	0.05
FXN	Frataxin	rs11145047	T/G	utr-3	1.07	7.97e-04	7	0.05
PCBP1	Poly(rC) binding protein 1	rs12319	C/T	synonymous-coding	0.97	2.56e-01	4	-
NCOA4	Nuclear Receptor Coactivator 4	rs10761618	T/C	intron	0.99	5.25e-01	2a	0.35
TAX1BP1	Tax1 Binding Protein 1	rs62449631	G/A	intron	1.03	4.56e-04	5	0.15

Table S45

Genome-wide association of iron regulatory proteins and schizophrenia based on GWAS results of CLOZUK.

A list of iron regulatory genes was based on the study by McAllum et al[14]. *NCOA4*, coding for the cargo receptor mediating ferritinophagy[26], and *TAX1BP1*, implicated in NCOA4-mediated ferritinophagy[27,28], were added to the list. Based on the CLOZUK GWAS[15], for each iron regulatory gene, the single nucleotide polymorphism (SNP) displaying the highest significance of association with schizophrenia is provided, alongside disease odds ratio (OR), association significance and the RegulomeDB and LINSIGHT scores. Except for *IREB2* (presented in Table S46), none of the iron regulatory genes displayed a genome-wide significant association with schizophrenia, defined as a $P < 5 \times 10^{-8}$. Data were extracted using SZDB2.0: an updated comprehensive resource for schizophrenia research[13].

[a]RegulomeDB is a database that annotates SNPs with known and predicted regulatory elements in the intergenic regions of the H. sapiens genome[29]. Lower scores are associated with stronger supporting data.

[b]LINSIGHT combines a generalized linear model for prediction of deleterious noncoding variants from functional and population genomic data, with scores ranging 0-1 (higher scores are more likely to have deleterious fitness consequences)[30].

a

Gene	Protein	Gene location	Index SNP	*P*-value (CLOZUK)	*P*-value (PGC2)
IREB2	Iron Responsive Element Binding Protein 2	chr15:78729773-78793798	rs3743078	3.11E-12	2.44E-13

b

Gene	Probe	Gene position	Index SNP	SNP position	P_{GWAS}	P_{eQTL}	P_{SMR}
IREB2	ENSG00000136381	chr15:78761785	rs4526984	chr15:78712119	1.67-e06	9.92-e10	1.63-e04

Table S46

***IREB2* - Association with schizophrenia and integrative (Mendelian randomization) analysis.**

(a) Based on the CLOZUK and PGC2, the index SNP (SNP with smallest P-value in this loci) alongside P-values of the index SNP in each GWAS are denoted. In both GWASs, genome-wide significant association with schizophrenia, defined as a $P < 5 \times 10^{-8}$, was detected.

(b) Summary data–based Mendelian (SMR) randomization analysis, with CLOZUK + PGC GWAS result[15,31] as GWAS input (P_{GWAS}) and Brain-eMeta eQTL summary data[32] as eQTL input (P_{GWAS}). Significance for the Summary data–based Mendelian (SMR) randomization analysis (P_{SMR})[16] was mined using SZDB2.0: an updated comprehensive resource for schizophrenia research[13].

Probe	Probe location[a]	MeanDiff[b]	P-value	q-value[c]	P-Bonf[d]	Description[e]
cg25428423	chr7:27780234	-0.008	7.05e-09	3.57e-06	3.22e-03	inside

Table S47

Differential methylation of *TAX1BP1* in schizophrenia prefrontal cortex.

Differential methylation data[17] were mined using SZDB2.0: an updated comprehensive resource for schizophrenia research[13].

[a]Position was based on hg19 genome assembly.

[b]If Methylation Change > 0, this methylation site is hypermethylated in schizophrenia. Otherwise, this site is hypermethylated in controls.

[c]The minimum false discovery rate at which the test may be called significant.

[d]A Bonferroni test is a type of multiple comparison test used in statistical analysis. "Bonferroni adjustment" suggests that the "p" value for each test must be equal to alpha divided by the number of tests.

[e]The relative location of methylation site and gene.

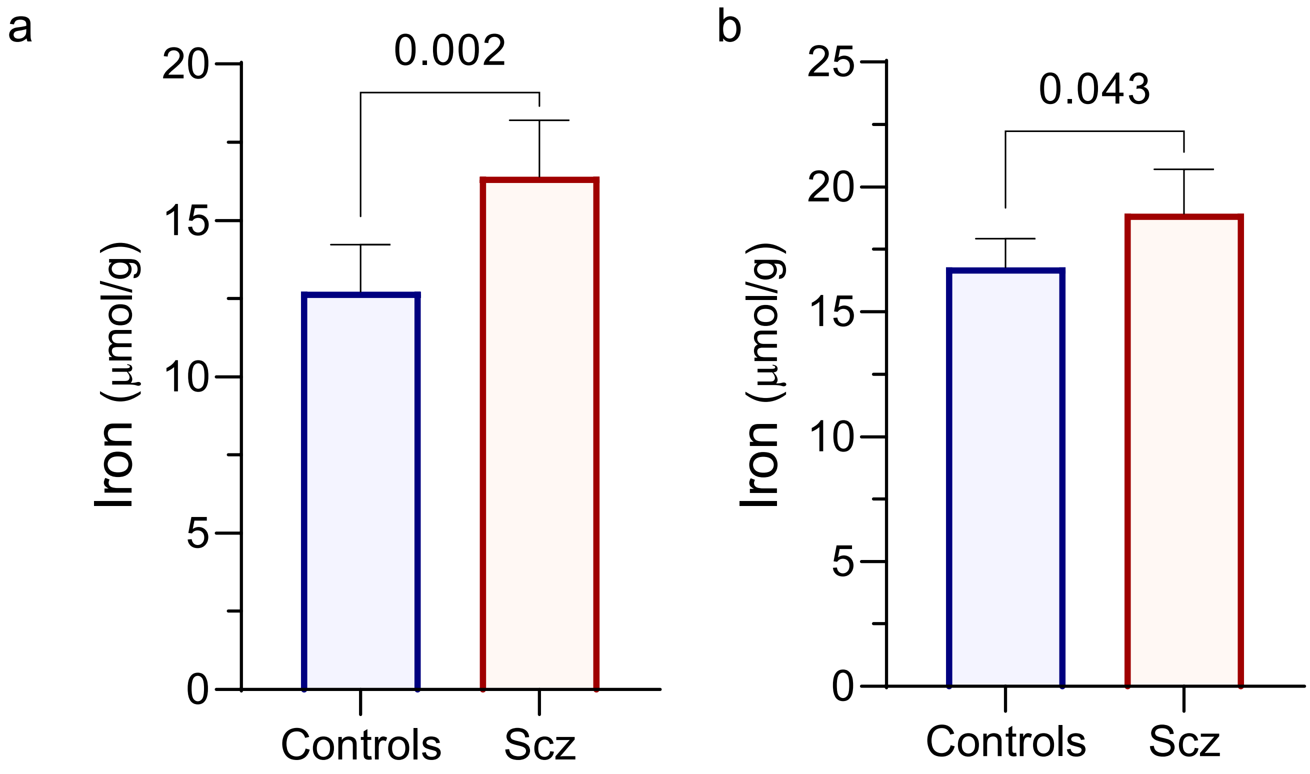

Figure S1 **Comparison of prefrontal iron content across diagnostic groups, related to Figure 1.**

(a) Bar graph depicting means (±95%CI) of iron (µmol/g) in samples of control subjects (n=37) and Scz cases (n=38) obtained from the NSW-BTRC. Significance of a two-sided student's t-test is denoted. Assumptions of equality of variances ($F_{36,37}$=0.672, p=0.235) and normality of residuals (Shapiro-Wilk W=0.969, p=0.060) were not violated.

(b) Bar graph depicting marginal means (±95%CI) of iron (µmol/g) in samples of control subjects (n=48) and Scz patients (n=48) obtained from the VBBN and NIMH-HBCC. Significance of an ANCOVA (with brain bank as co-variate, robust standard errors) is denoted.

As absolute iron values in the replication sample were higher, possibly reflecting slight differences in extraction procedures applied for specimens across different brain banks, we used z-scores derived from the control distribution of each cohort when pooling all samples together.

NSW-BTRC, New South Wales Brain Tissue Resource Centre; VBBN, Victoria Brain Bank Network; NIMH-HBCC, National Institute of Mental Health Human Brain Collection Core.

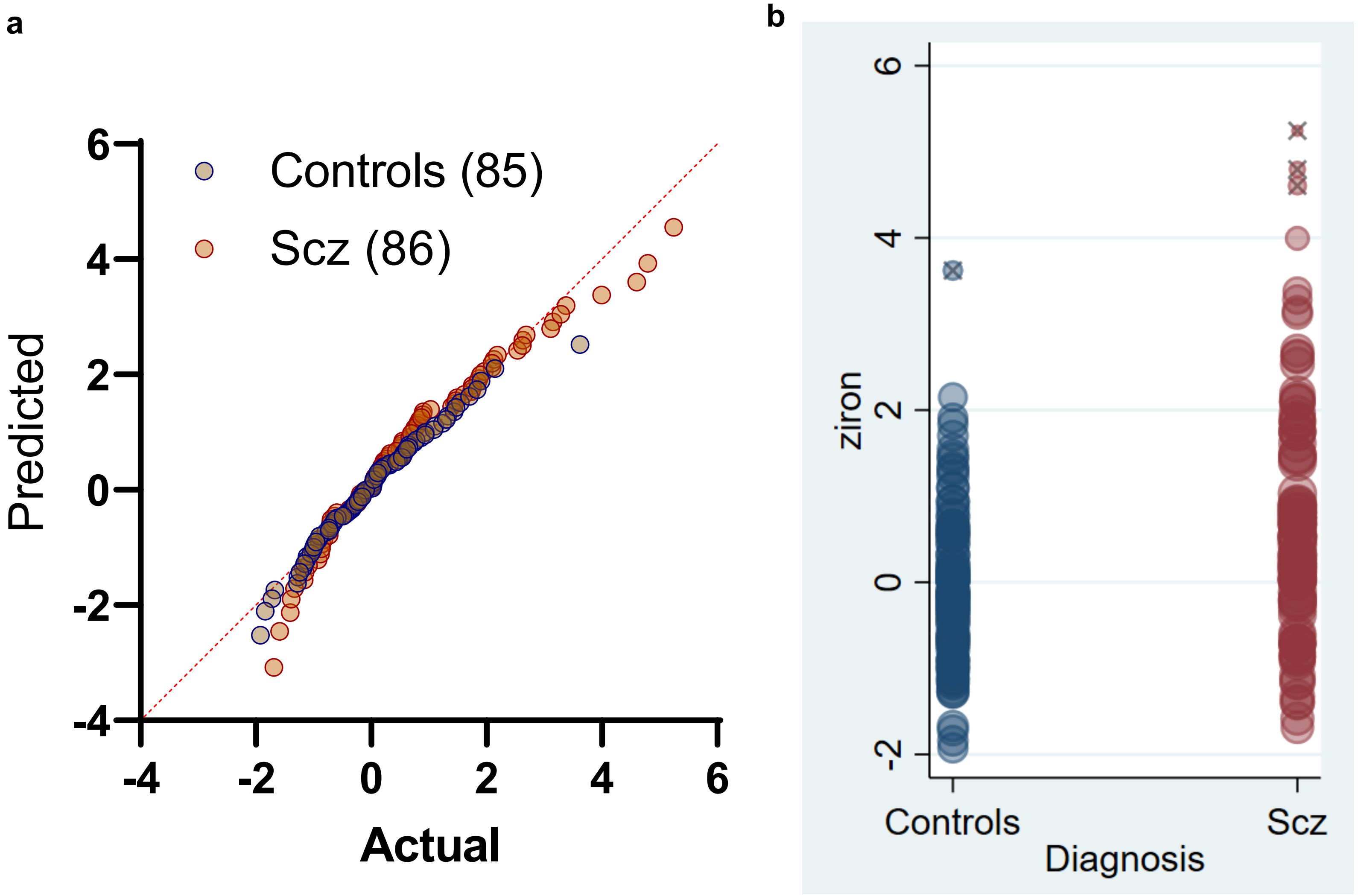

Figure S2 **Iron distribution in the Combined Cohort across diagnostic groups, related to Figure 1.**

(a) Normal Q-Q plot depicting actual (x-axis) vs. predicted (y-axis) iron values (presented as z-scores derived from the control distribution), for 85 control subjects (blue-border circles) and 86 Scz cases (red-border circles) in the combined cohort. Assumptions of normality were not violated, based on robust tests of normality (Table S4).

(b) Robust comparison of iron levels across diagnostic groups. Individual data points depicting prefrontal iron (y-axis, z-scores) against diagnosis (x-axis) are presented. Symbol size corresponds to analytic weight derived using a robust (IRWLS MM-estimators) regression model. Outliers markedly downweighed in the analysis (i.e., weight<0.3) are overlayed with a gray cross, and while included in the robust regression model, these points were excluded from the graphical display in the Main Figure. z-scores were derived using controls' distribution. $n_{controls}$=85, $n_{schizophrenia}$ =86. Related to Figure 1a

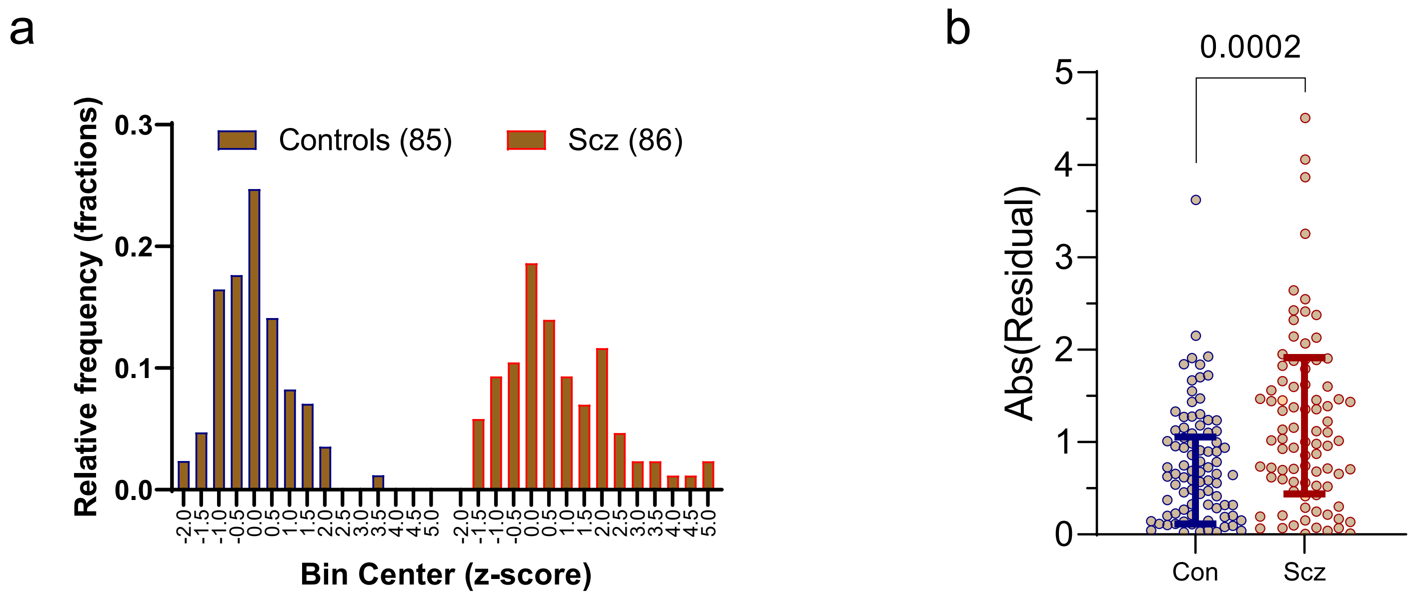

Figure S3 **Iron distribution in the Combined Cohort, related to Figure 1.**

(a) Frequency histogram. Relative frequency (y-axis) of iron levels (binned control-derived z-scores, x-axis) among control subjects (brown boxes) and Scz patients (red boxes) is presented. Patients' distribution is wider.

(b) Homoscedasticity plot. Scatter plot depicting absolute residuals (±SD) derived from an unpaired t-test of iron by diagnosis. Significance of F test to compare variances (F=2.29, DFn=85, Dfd=84) is denoted (see also Table S6).

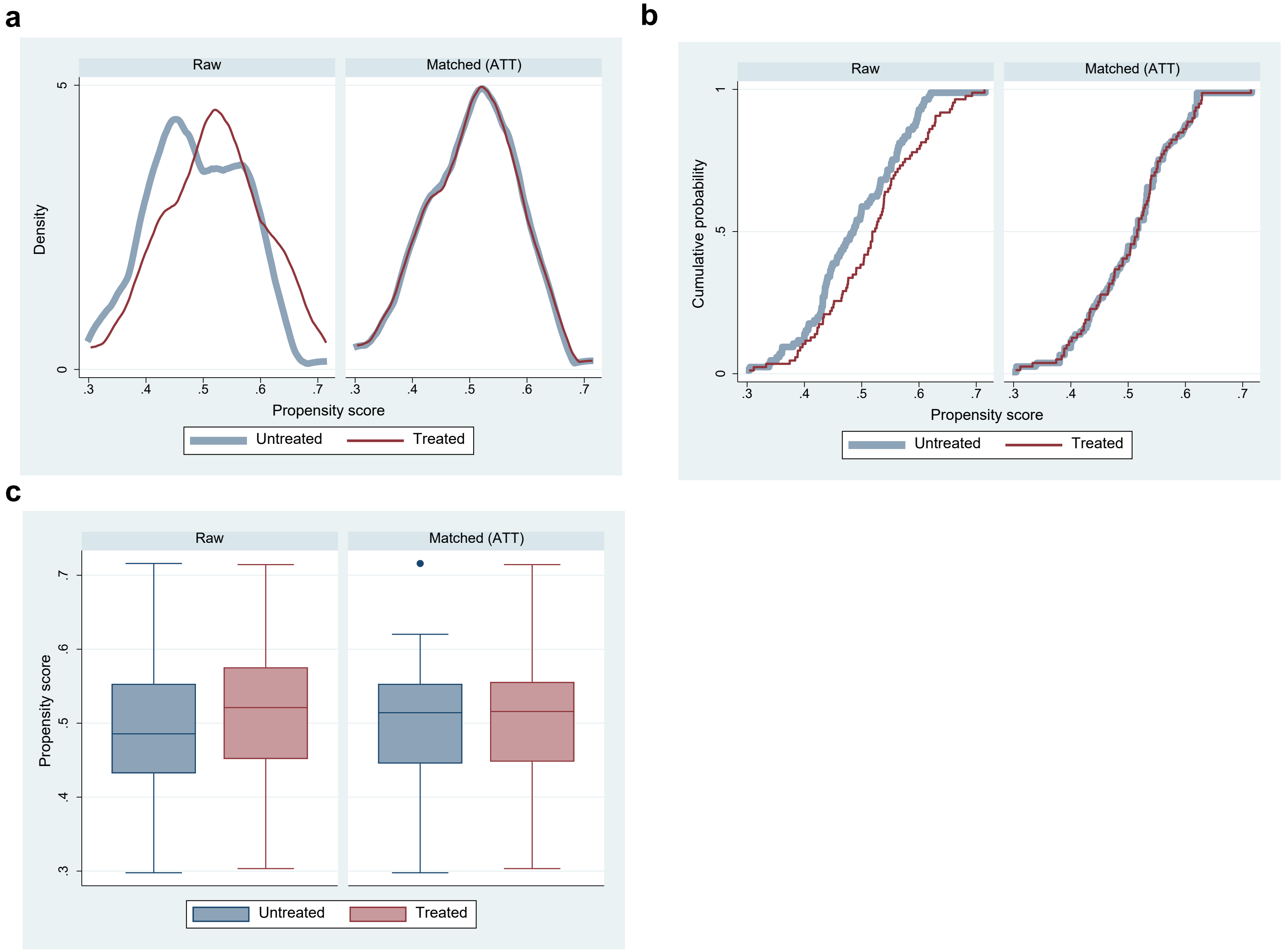

Figure S4 **Balancing plots for PFC iron across diagnosis based on regression-adjusted propensity-score matching, related to Figure 1.**

Following propensity-score kernel matching with age, sex, race, pH and PMI as covariates, based on a logit model where diagnosis of schizophrenia was defined as Treatment, balancing plots depicting raw (left) and PS-matched (right) data are presented, based on KMATCH, Stata's module for multivariate-distance and propensity-score matching.

(a) Kernel density balancing plots.

(b) Kernel cumulative probability balancing plots.

(c) Kernel propensity-score balancing box plots.

PMI, post-mortem interval. PS, propensity-score. See also Table S8.

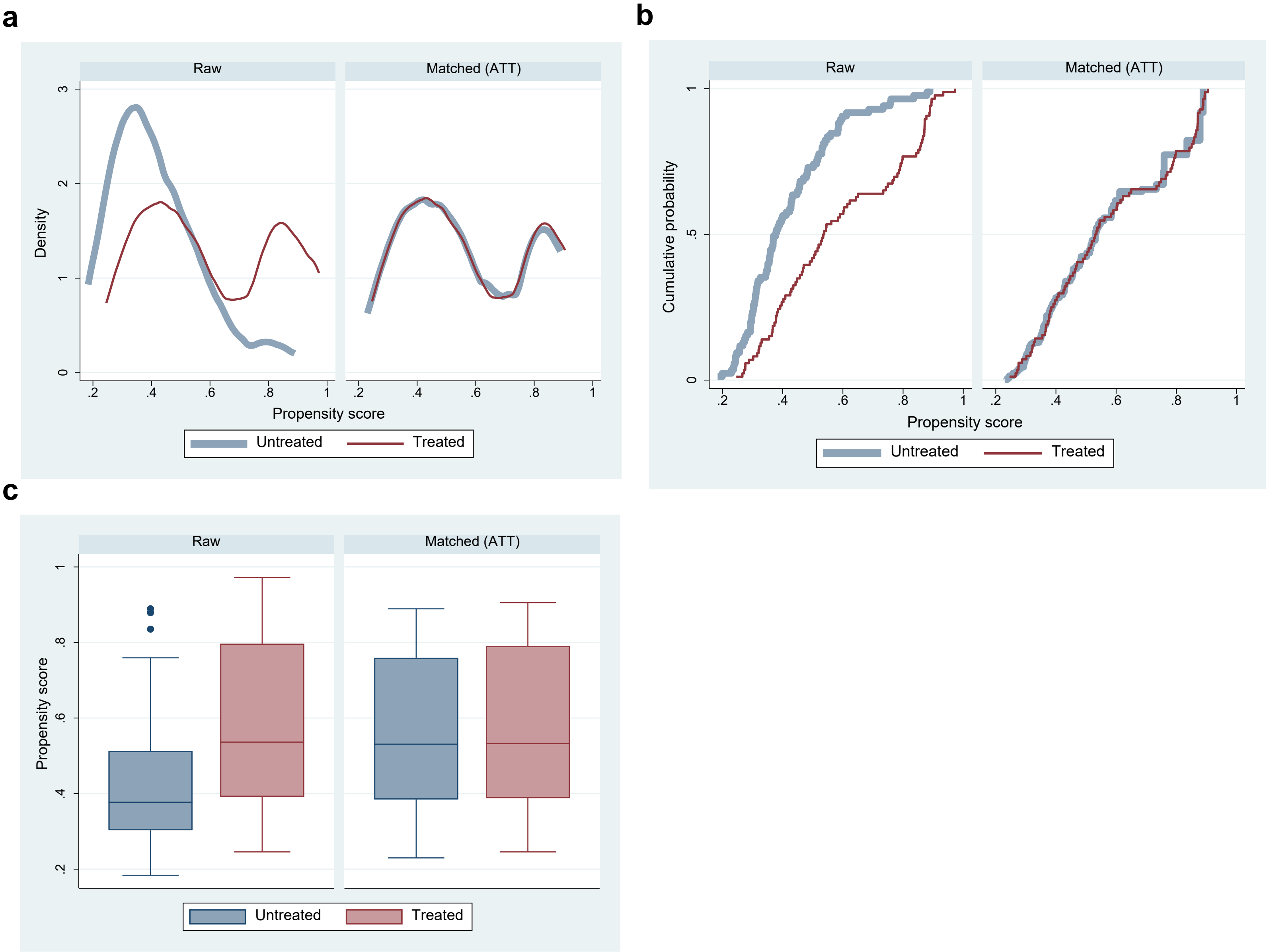

Figure S5 **Balancing plots for PFC iron across diagnosis based on regression-adjusted propensity-score matching, including mode of death as a covariate, related to Figure 1.**

Following propensity-score kernel matching with age, pH and PMI as covariates, and including also mode of death as a covariate, based on a logit model where diagnosis of schizophrenia was defined as Treatment, balancing plots depicting raw (left) and PS-matched (right) data are presented, based on KMATCH, Stata's module for multivariate-distance and propensity-score matching. Note marked separation between raw and adjusted data (compared to Figure S4), largely reflecting gross inequality in mode of death across groups.

(a) Kernel density balancing plots.
(b) Kernel cumulative probability balancing plots.
(c) Kernel propensity-score balancing box plots.

PMI, post-mortem interval. PS, propensity-score. See also Table S9.

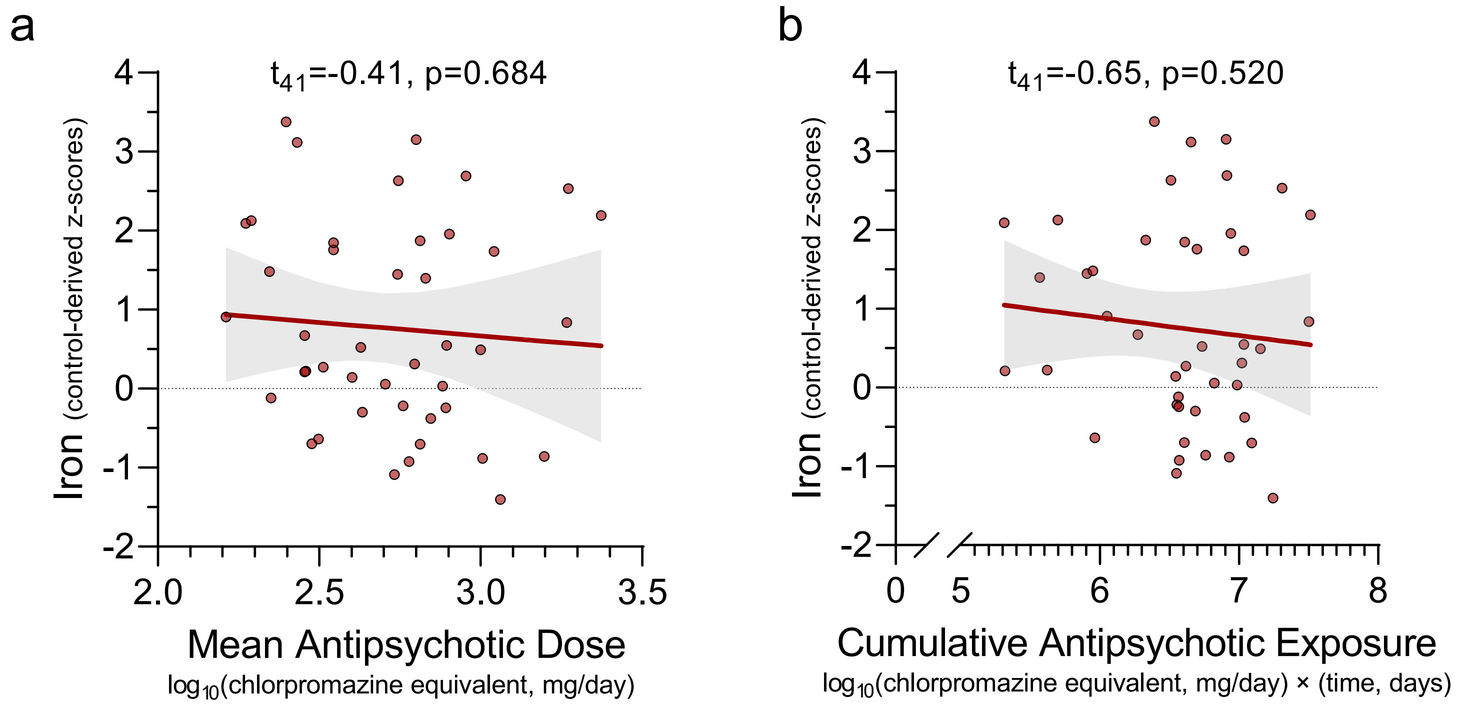

Figure S6 **Predicting iron content by antipsychotic treatment, related to Figure 1.**

Robust regression estimates predicting PFC iron content (y-axis, control-derived z-scores) based on (a) mean daily dose of antipsychotic medication (x-axis, log_{10}[chlorpromazine equivalent, mg/day) or on (b) cumulative lifetime exposure to antipsychotic medication (x-axis, log_{10}[chlorpromazine equivalent, mg/day) × (time, days]). Regression lines with 95% Cis are depicted. Denoted above are t-statistics and corresponding significance values for the predictor. Based on Stata's "robreg mm" command. Red circles denote individual values. n_{Scz}=43.

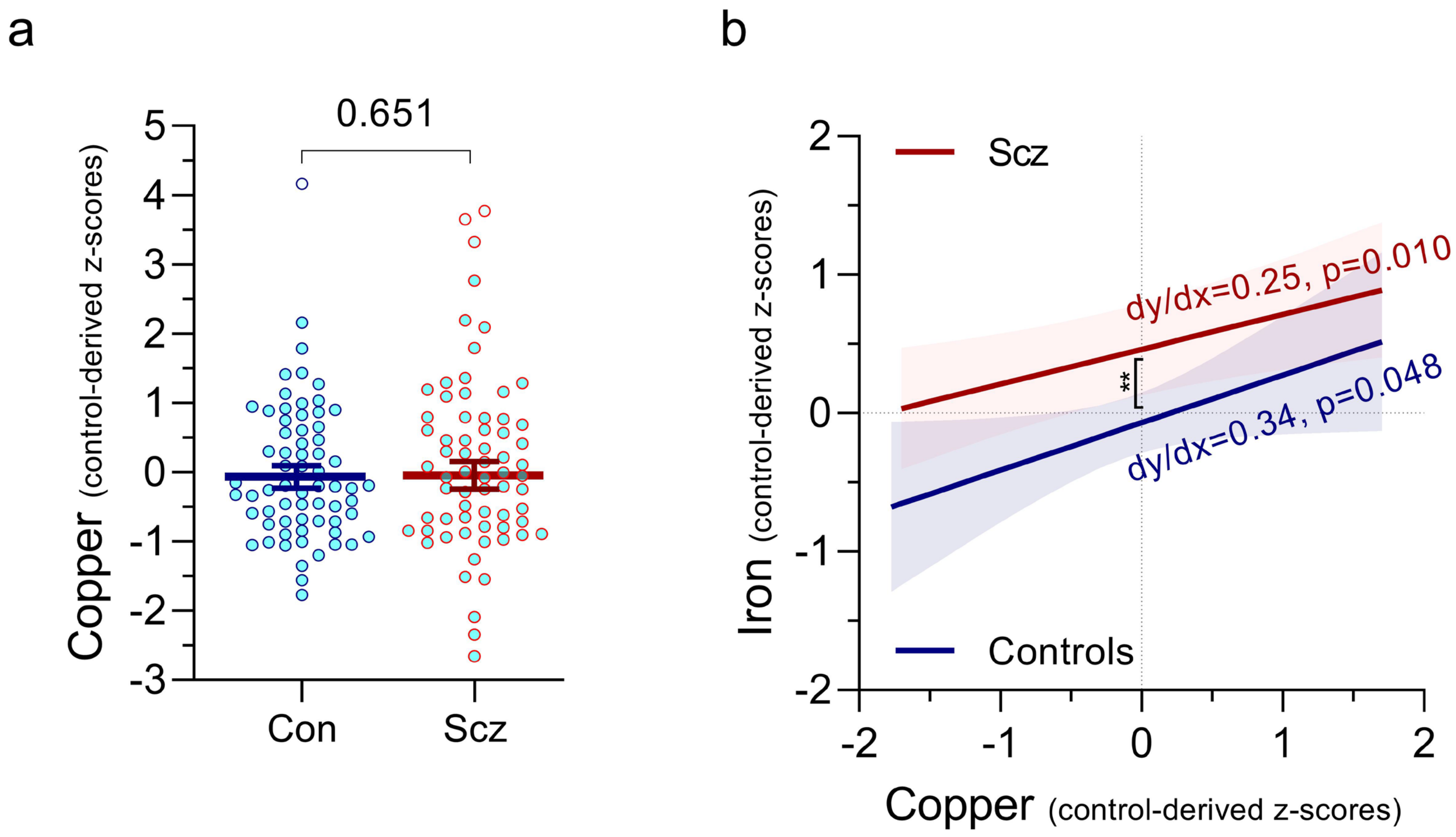

Figure S7 **Prefrontal copper across diagnostic groups and its effect on iron.**

(a) Comparison of copper levels across diagnostic groups. Scatter plot with bars depicting marginal means (±95%CI) of PFC copper (presented as z-scores) among control individuals and schizophrenia cases. Value above the bar represents significance of diagnosis effect. Outliers downweighed in the analysis appear as transparent data points. Based on a robust (IRWLS MM-estimators regression model, Table S19).

(b) Effects of copper on iron in controls and cases. Predicted marginal means (±95%CI) of iron (y-axis, control derived z-scores) according to copper (x-axis, control derived z-scores) plotted for each diagnostic group. Based on a robust (IRWLS MM-estimators) regression model with copper, diagnosis and their interaction term as predictors (Table S20). **P<0.01 for between-group difference in iron at control-derived mean copper levels.

$n_{controls}$=66, n_{scz}=67.

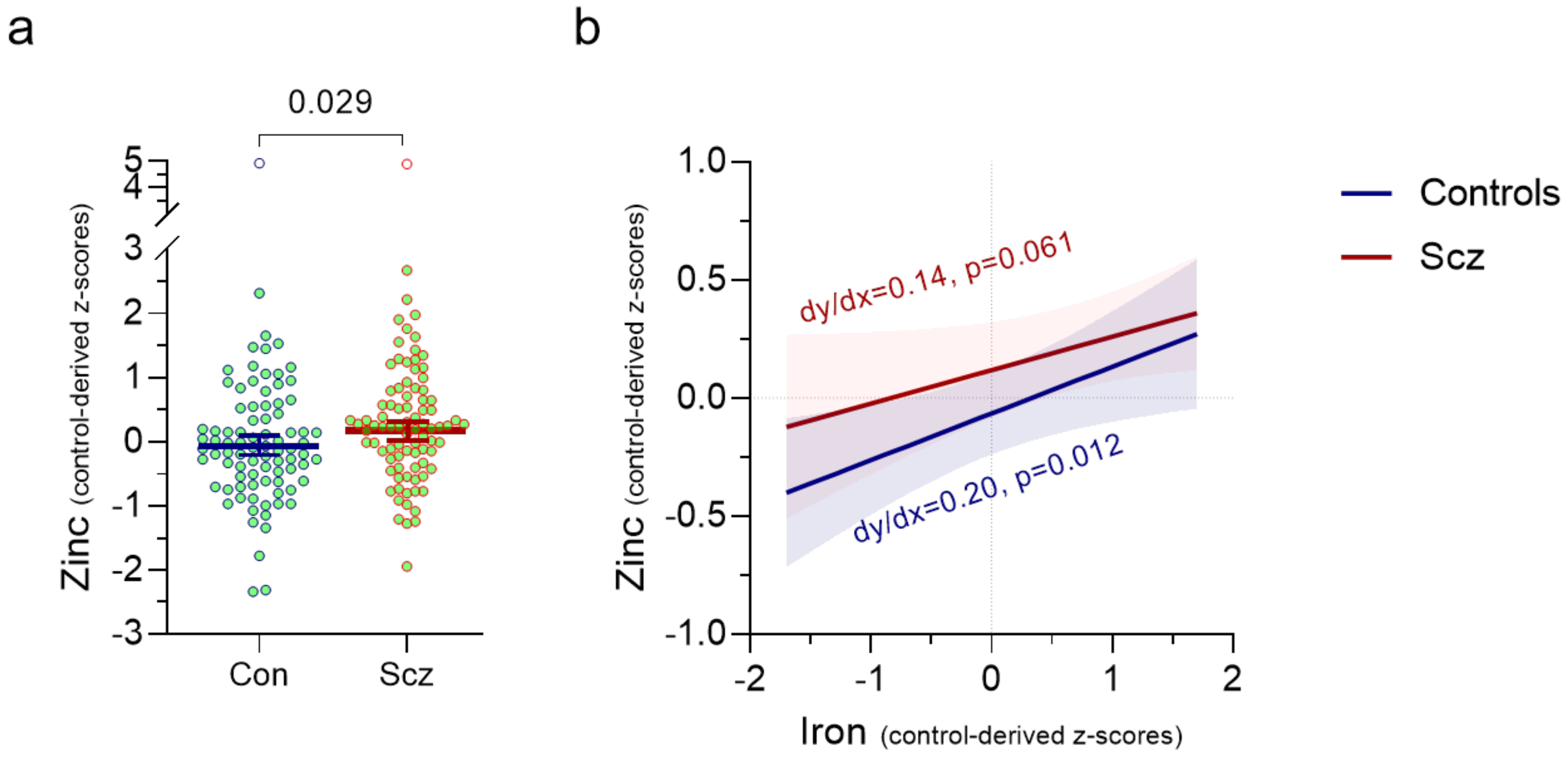

Figure S8 **Prefrontal zinc across diagnostic groups and its dependence on iron.**

(a) Comparison of zinc levels across diagnostic groups. Scatter plot with bars depicting marginal means (±95%CI) of PFC zinc (presented as z-scores) among control individuals and schizophrenia cases. Value above the bar represents significance of diagnosis effect. Outliers downweighed in the analysis appear as transparent data points. Based on a robust (IRWLS MM-estimators regression model, Table S22).

(b) Effects of iron on zinc in controls and cases. Predicted marginal means (±95%CI) of zinc (y-axis, control derived z-scores) according to iron (x-axis, control derived z-scores) plotted for each diagnostic group. Based on a robust (IRWLS MM-estimators) regression model with iron, diagnosis and their interaction term as predictors (Table S23).

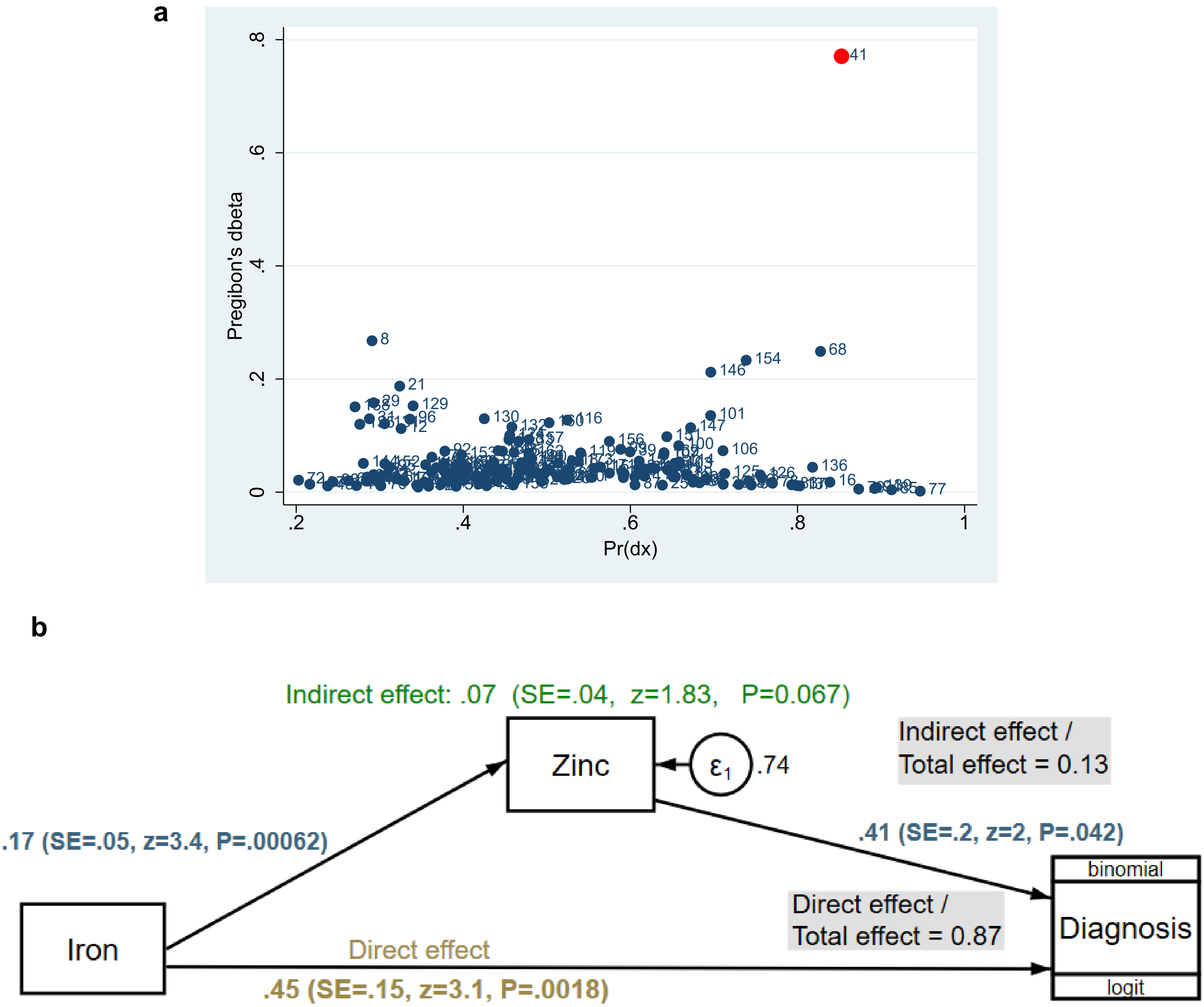

Figure S9 **Exploring the effect of iron on diagnosis through zinc.**

(a) Following a logistic regression predicting diagnosis by iron and zinc (N=171) while controlling for sex, ethnicity, sample pH and post-mortem interval, each observation's influence on the regression coefficient (Pregibon's dbeta) is depicted (y-axis) against the predicted probability for diagnosis of schizophrenia (x-axis). As visualized, the influence exerted by sample #41 was unusually high so this sample was excluded. Thus, the structural equation model presented in panel b was based on a total of 170 samples.

(b) Exploring the effect of iron on diagnosis through zinc. With iron and zinc as continuous variables (z-scores adjusted for the covariates sex, age, race, pH and log_2PMI) and diagnosis as a binomial variable, the direct effect of iron on diagnosis and the effects of iron on zinc and zinc on diagnosis were quantified using a general structural equation model. For each effect, coefficient is provided alongside standard error (SE), z-score and significance in brackets. Significance of indirect effect (iron on diagnosis via zinc) was estimated using bootstrap-generated confidence intervals. The highly significant direct effect of iron on diagnosis was over five times larger than the non-significant indirect effect of iron on diagnosis, indicating that from a statistical viewpoint, the risk for a schizophrenia diagnosis conferred by elevated iron was unlikely to be mediated via zinc.

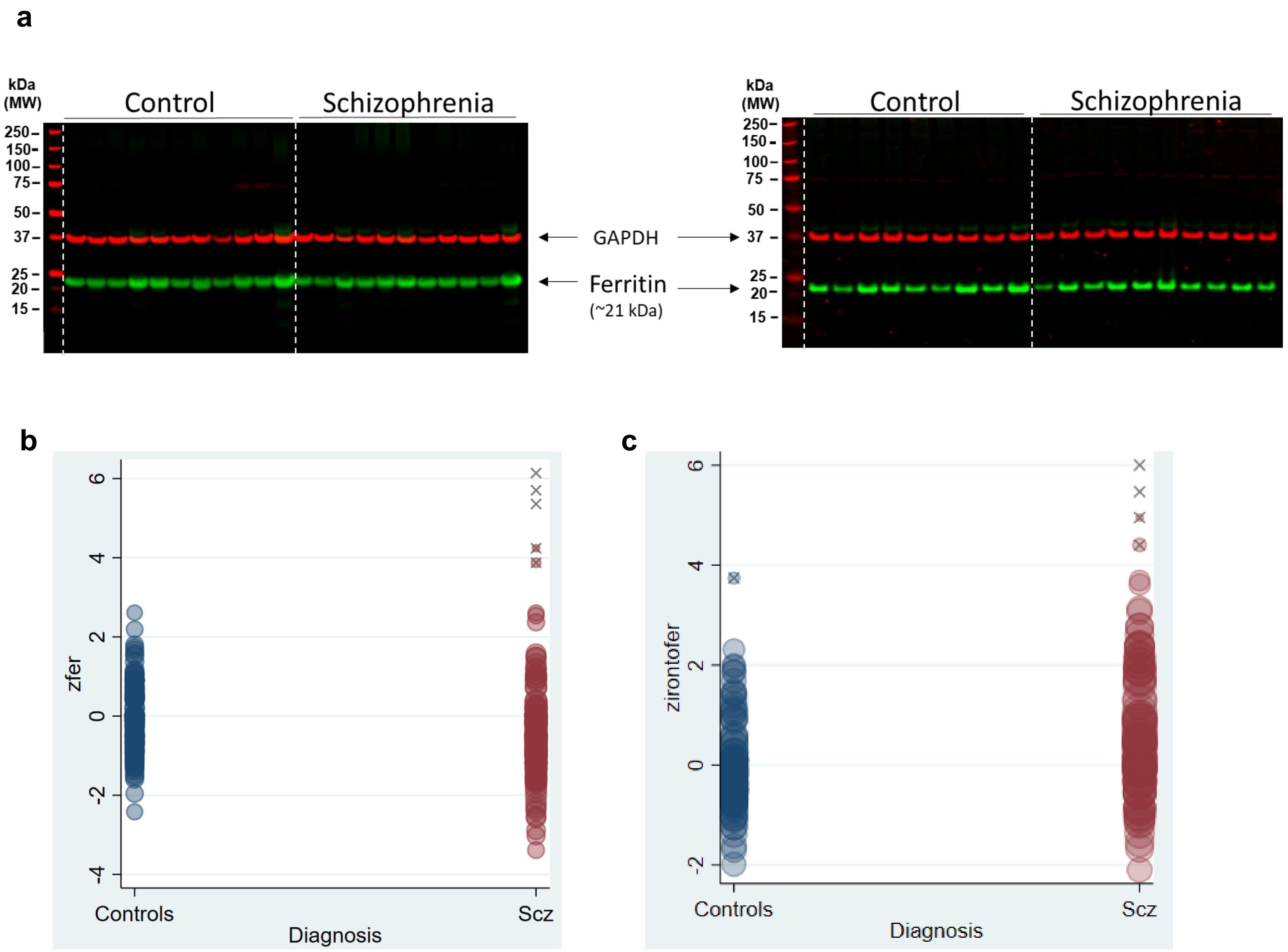

Figure S10 **Ferritin across diagnostic groups, related to Figure 1.**

(a) Western blot. Representative blots from control subjects and Scz patients demonstrating an expected band at ~21 kDa reflecting ferritin.

(b) Robust comparison of ferritin levels across diagnostic groups. Individual data points depicting prefrontal ferritin (y-axis, z-scores) against diagnosis (x-axis) are presented. Symbol size corresponds to analytic weight derived using a robust (IRWLS MM-estimators) regression model. Outliers markedly downweighed in the analysis (i.e. weight<0.3) are overlayed with a gray cross, and while included in the robust regression model, these points were excluded from the graphical display in the Main Figure. z-scores were derived using controls' distribution. $N_{controls}$=85, $N_{schizophrenia}$ =86. Related to Figure 1b.

(c) Robust comparison of iron-to-ferritin ratio across diagnostic groups. Individual data points depicting prefrontal iron-to-ferritin ratio (y-axis, z-scores) against diagnosis (x-axis) are presented. Symbol size corresponds to analytic weight derived using a robust (IRWLS MM-estimators) regression model. Outliers markedly downweighed in the analysis (i.e. weight<0.3) are overlayed with a gray cross, and while included in the robust regression model, these points were excluded from the graphical display in the Main Figure. z-scores were derived using controls' distribution. $N_{controls}$=85, $N_{schizophrenia}$ =86. Related to Figure 1c.

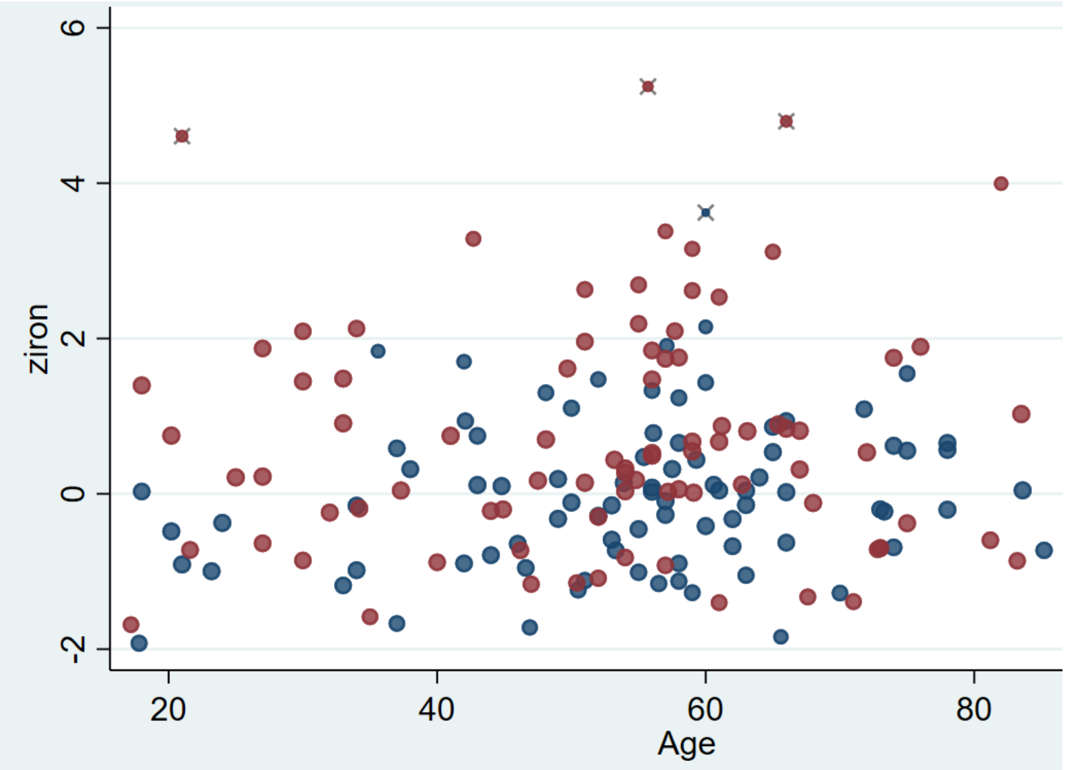

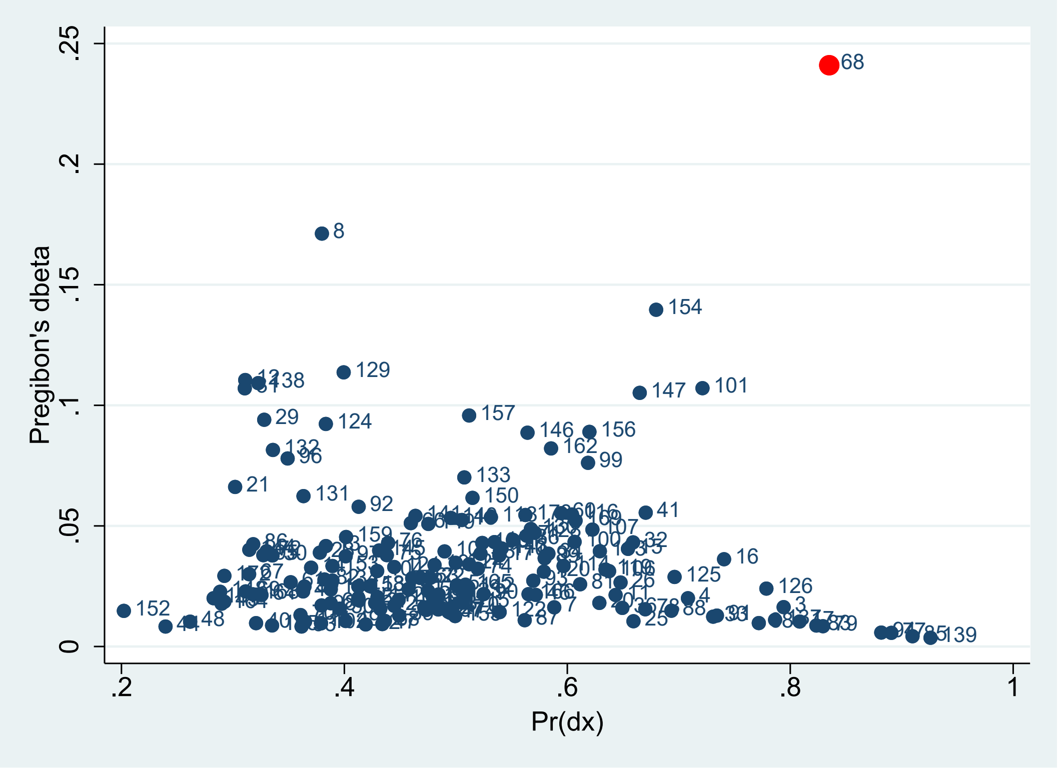

Figure S11 **Identifying highly influential/ irregular observations, related to Figure 2.**

(a) Relationship between age and iron in controls and patients. Individual data points depicting prefrontal iron (y-axis, z-scores) against age of death (x-axis, years) are presented for each diagnostic group (blue, controls; red, cases). Symbol size corresponds to analytic weight derived using robust (IRWLS MM-estimators) regression models. Outliers downweighed in the analysis (i.e. weight<0.5) are overlayed with a gray cross, and while included in the robust regression model, these points were excluded from the graphical display in the Main Figure. z-scores were derived using controls' distribution. $n_{controls}$=85, $n_{schizophrenia}$ =86. Related to Figure 2a**.**

(b) Following a logistic regression predicting diagnosis by iron (N=171) while controlling for sex, ethnicity, sample pH and post-mortem interval, each observation's influence on the regression coefficient (Pregibon's dbeta) is depicted (y-axis) against the predicted probability for diagnosis of schizophrenia (x-axis). As visualized, the influence exerted by sample #68 (an individual in the control group) was unusually high so this sample was excluded. Thus, the analyses presented in Figure 2b-d were based on a total of 170 (84 controls, 86 cases) samples. Related to Figure 2b-d.

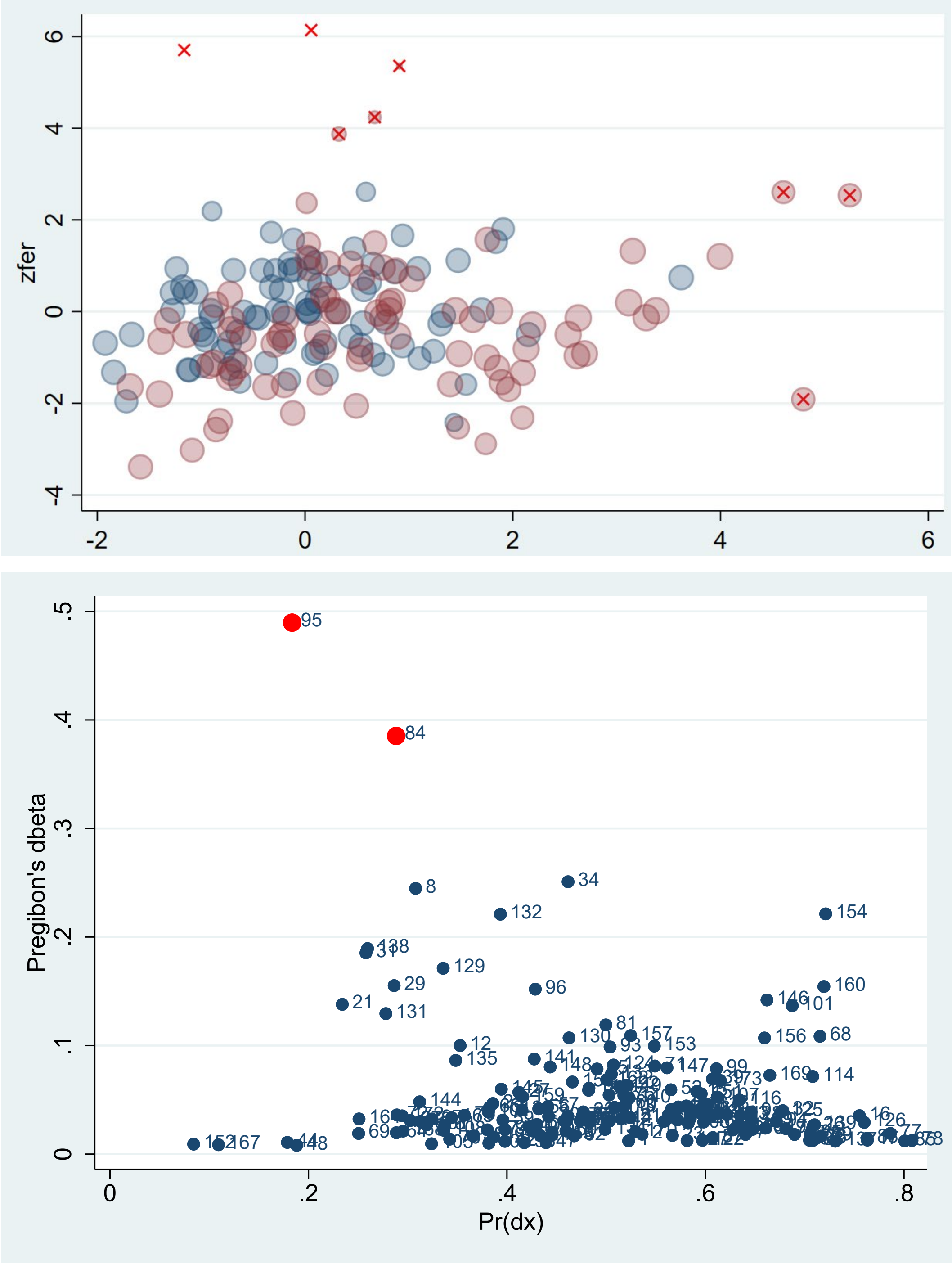

Figure S12 **Identifying highly influential/ irregular observations, related to Figure 3.**

(a) Relationship between iron and ferritin in controls and patients. Individual data points depicting prefrontal ferritin (y-axis, z-scores) against iron (x-axis, z-scores) are presented for each diagnostic group (blue, controls; red, cases). Symbol size corresponds to analytic weight derived using robust (IRWLS MM-estimators) regression models. Outliers markedly downweighed in the analysis (i.e., weight<0.5) and high-leverage data points (i.e., iron>4 SD) are overlayed with a red cross, and while included in the robust regression model, these points were excluded from the graphical display in the Main Figure. z-scores were derived using controls' distribution. $n_{controls}$=85, $n_{schizophrenia}$ =86. Related to Figure 3a**.**

(b) Following a logistic regression predicting diagnosis by ferritin (N=171) while controlling for log-iron, age, sex, ethnicity, sample pH and post-mortem interval, each observation's influence on the regression coefficient (Pregibon's dbeta) is depicted (y-axis) against the predicted probability for diagnosis of schizophrenia (x-axis). As visualized, the influence exerted by samples #84 and #95 (two individuals in the schizophrenia group) was unusually high so these samples were excluded. Thus, the analyses presented in Figure 3b-d were based on a total of 169 (85 controls, 84 cases) samples.

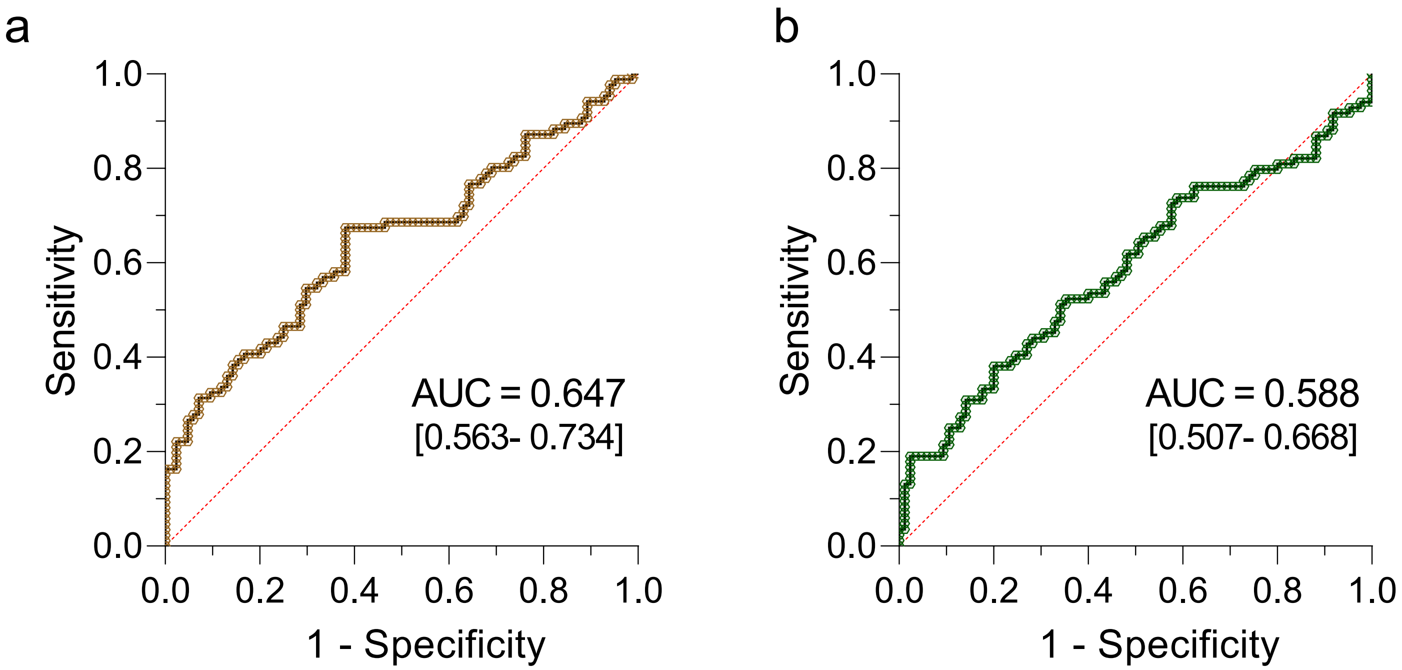

Figure S13 **Discriminating schizophrenia patients from controls based on prediction models, related to Figure 4.**

(a) Discriminatory performance of iron (adjusted for age, sex, ethnicity, sample pH and post-mortem interval) among 170 individuals.

(b) Discriminatory performance of ferritin (adjusted for age, sex, ethnicity, sample pH and post-mortem interval) among 169 individuals.

For each logistic mode, the area-under-curve [AUC] parameter [95% bias-corrected CIs] of a receiver operating characteristic [ROC]) is denoted.

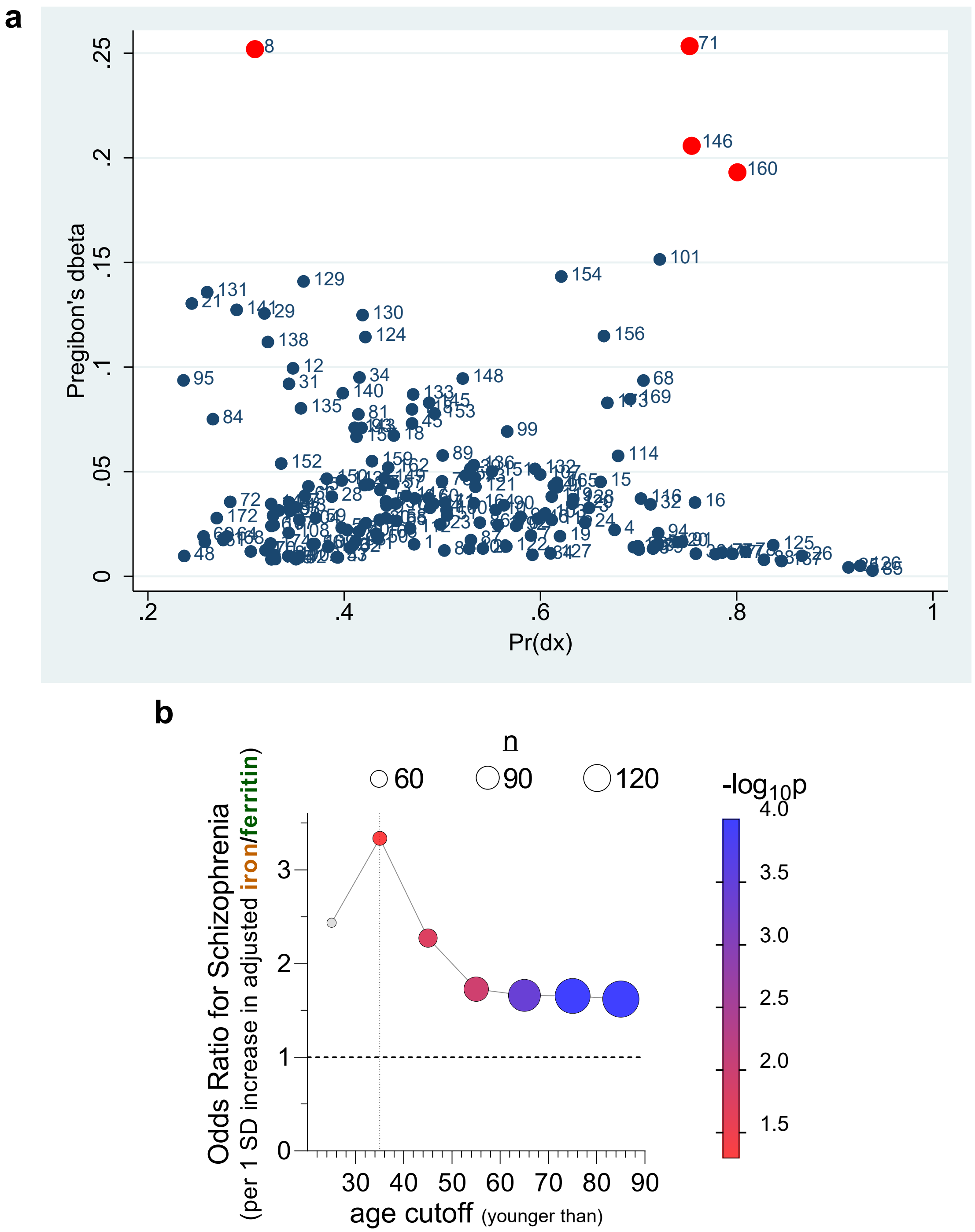

Figure S14 **Logistic regression model predicting schizophrenia based on iron-to-ferritin ratio, related to Figure 4.**

(a) Following a logistic regression predicting diagnosis by iron-to-ferritin ratio (N=171) while controlling for age, sex, ethnicity, sample pH and log-post-mortem interval, each observation's influence on the regression coefficient (Pregibon's dbeta) is depicted (y-axis) against the predicted probability for diagnosis of schizophrenia (x-axis). As visualized, the influence exerted by samples #8, #71, #146 and #160 was unusually high so these samples were excluded. Thus, the analyses presented in Figure 4 and in the subsequent panel was based on a total of 167 samples.

(b) Odds ratio of having schizophrenia per unit iron/ferritin increase, according to age of death. Based on serial logistic regression analyses, the odds ratio of having a diagnosis of schizophrenia (as compared to being a control) attributed to a 1 SD increase in covariate (sex, ethnicity, sample pH and post-mortem interval)-adjusted iron-to-ferritin ratio (y-axis) is plotted against age cutoff (x-axis, years). Using ten-year steps beginning at age 25, each analysis included only individuals younger than the designated age cutoff. For each analysis, symbol size represents number of individuals included (n, upper-center legend), and symbol colour represents significance of iron in predicting diagnosis (-log10-p-value, right-hand legend). While the odds ratio was maximal when the analysis included only individuals younger than 35, at this age cutoff the model did not provide superior discrimination compared to a model based on iron alone.

a

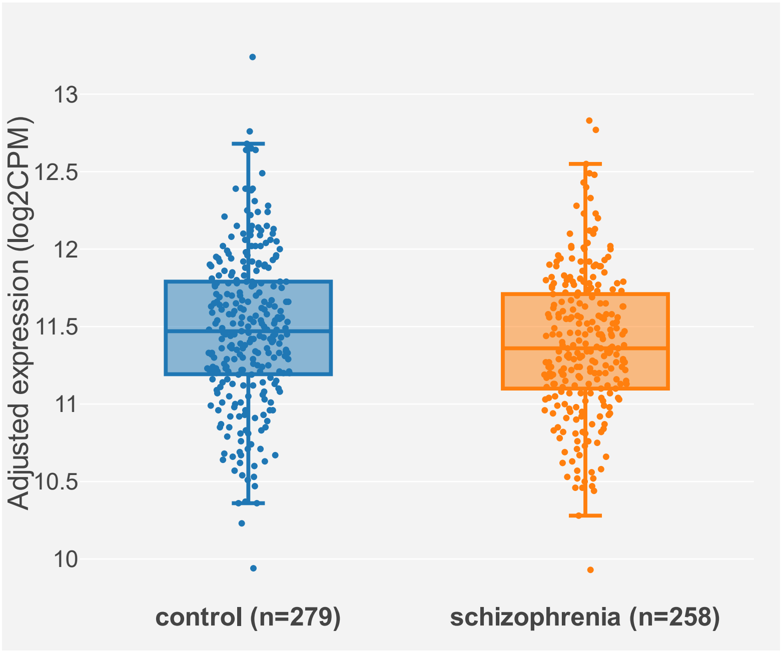

b

Gene	Ensembl	Genome Position[a]	logFC[b]	Avg Expression	T Statistic[c]	*P*-value	FDR[d]
FTH1	ENSG00000167996	chr11:61727190-61735132	-0.091	6.549	-1.972	4.91e-02	2.34e-01

Figure S15 **Differential expression of FTH1 in schizophrenia prefrontal cortex.**

(a) Adjusted expression (log2CPM) of FTH1.
(b) Parameters and results for differential expression.
Data are based on the CommonMind Consortium sequenced RNA from dorsolateral prefrontal cortex of people with schizophrenia (N = 258) and control subjects (N = 279, Nature Neuroscience, 19(11):1442-1453), obtained via SZDB2.0, an updated comprehensive resource for schizophrenia research (Human Genetics (2020) 139:1285–1297).

[a]Genome Position, based on hg19 genome assembly.

[b]logFC, log2(Fold Change). Fold change is the ratio of the expression value of a gene in schizophrenia cases to the expression of healthy controls. If logFC > 0, this gene is highly expressed in schizophrenia. Otherwise, this gene is highly expressed in controls.

[c]T Statistic, the T Statistic is used in a T test when deciding whether we should support or reject the null hypothesis.

[d]FDR, the false discovery rate (FDR) is a method of conceptualizing the rate of type I errors in null hypothesis testing when conducting multiple comparisons.

a

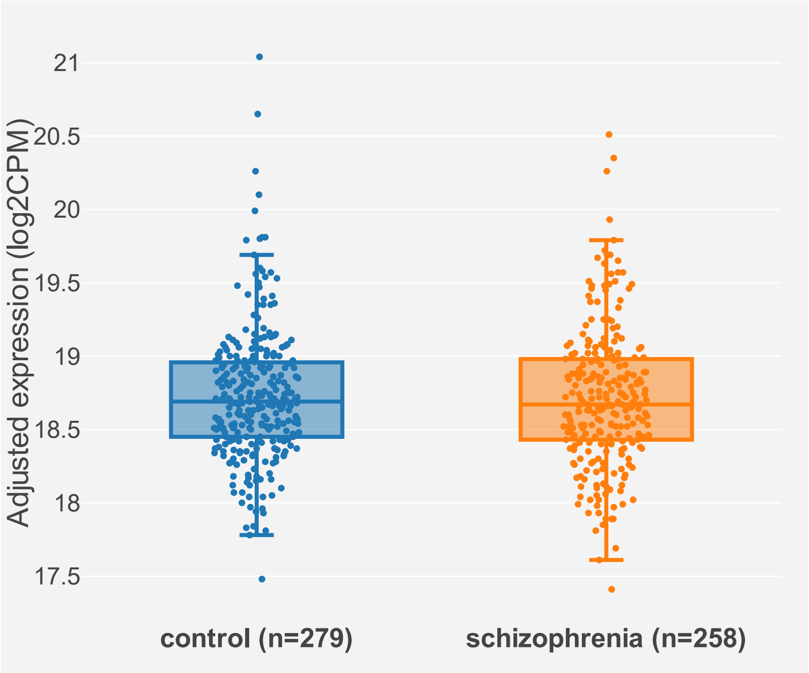

b

Gene	Ensembl	Genome Position[a]	logFC[b]	Avg Expression	T Statistic[c]	*P*-value	FDR[d]
FTL	ENSG00000087086	chr19:49468558-49470135	-0.021	8.816	-0.488	6.26e-01	8.15e-01

Figure S16 **Differential expression of FTL in schizophrenia prefrontal cortex.**

(a) Adjusted expression (log2CPM) of FTL.
(b) Parameters and results for differential expression.
Data are based on the CommonMind Consortium sequenced RNA from dorsolateral prefrontal cortex of people with schizophrenia (N = 258) and control subjects (N = 279, Nature Neuroscience, 19(11):1442-1453), obtained via SZDB2.0, an updated comprehensive resource for schizophrenia research (Human Genetics (2020) 139:1285–1297).

[a]Genome Position, based on hg19 genome assembly.

[b]logFC, log2(Fold Change). Fold change is the ratio of the expression value of a gene in schizophrenia cases to the expression of healthy controls. If logFC > 0, this gene is highly expressed in schizophrenia. Otherwise, this gene is highly expressed in controls.

[c]T Statistic, the T Statistic is used in a T test when deciding whether we should support or reject the null hypothesis.

[d]FDR, the false discovery rate (FDR) is a method of conceptualizing the rate of type I errors in null hypothesis testing when conducting multiple comparisons.

a

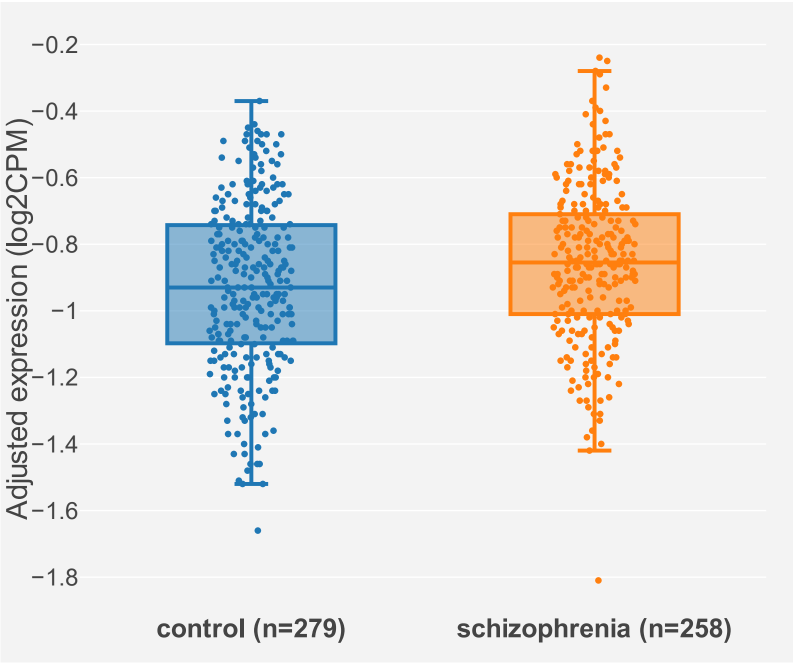

b

Gene	Ensembl	Genome Position[a]	logFC[b]	Avg Expression	T Statistic[c]	*P*-value	FDR[d]
IREB2	ENSG00000136381	chr15:78729773-78793798	0.073	6.909	3.041	2.47e-03	5.71e-02

Figure S17 **Differential expression of IREB2 in schizophrenia prefrontal cortex.**

(a) Adjusted expression (log2CPM) of IREB2.
(b) Parameters and results for differential expression.

Data are based on the CommonMind Consortium sequenced RNA from dorsolateral prefrontal cortex of people with schizophrenia (N = 258) and control subjects (N = 279, Nature Neuroscience, 19(11):1442-1453), obtained via SZDB2.0, an updated comprehensive resource for schizophrenia research (Human Genetics (2020) 139:1285–1297).

[a]Genome Position, based on hg19 genome assembly.

[b]logFC, log2(Fold Change). Fold change is the ratio of the expression value of a gene in schizophrenia cases to the expression of healthy controls. If logFC > 0, this gene is highly expressed in schizophrenia. Otherwise, this gene is highly expressed in controls.

[c]T Statistic, the T Statistic is used in a T test when deciding whether we should support or reject the null hypothesis.

[d]FDR, the false discovery rate (FDR) is a method of conceptualizing the rate of type I errors in null hypothesis testing when conducting multiple comparisons.

a

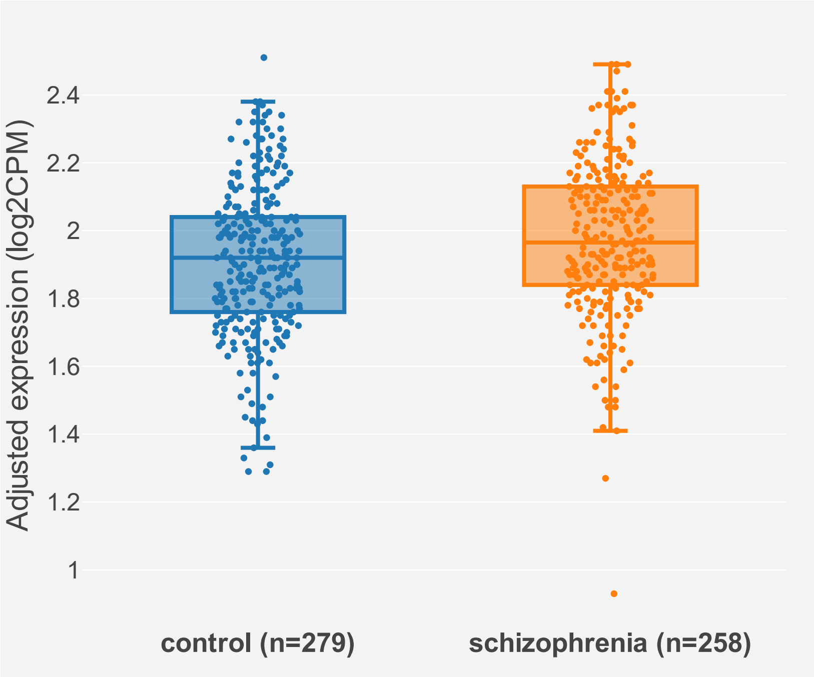

b

Gene	Ensembl	Genome Position[a]	logFC[b]	Avg Expression	T Statistic[c]	*P*-value	FDR[d]
TAX1BP1	ENSG00000106052	chr7:27778950-27884183	0.063	7.819	2.840	4.67e-03	7.77e-02

Figure S18 **Differential expression of TAX1BP1 in schizophrenia prefrontal cortex.**

(a) Adjusted expression (log2CPM) of TAX1BP1.
(b) Parameters and results for differential expression of TAX1BP1.
Data are based on the CommonMind Consortium sequenced RNA from dorsolateral prefrontal cortex of people with schizophrenia (N = 258) and control subjects (N = 279, Nature Neuroscience, 19(11):1442-1453), obtained via SZDB2.0, an updated comprehensive resource for schizophrenia research (Human Genetics (2020) 139:1285–1297).

[a]Genome Position, based on hg19 genome assembly.

[b]logFC, log2(Fold Change). Fold change is the ratio of the expression value of a gene in schizophrenia cases to the expression of healthy controls. If logFC > 0, this gene is highly expressed in schizophrenia. Otherwise, this gene is highly expressed in controls.

[c]T Statistic, the T Statistic is used in a T test when deciding whether we should support or reject the null hypothesis.

[d]FDR, the false discovery rate (FDR) is a method of conceptualizing the rate of type I errors in null hypothesis testing when conducting multiple comparisons.

Supplementary References

1 Bousman, C. A. *et al.* Elevated ubiquitinated proteins in brain and blood of individuals with schizophrenia. *Scientific Reports* **9**, 2307, doi:10.1038/s41598-019-38490-1 (2019).

2 Dean, B., Tsatsanis, A., Lam, L. Q., Scarr, E. & Duce, J. A. Changes in cortical protein markers of iron transport with gender, major depressive disorder and suicide. *The World Journal of Biological Psychiatry* **21**, 119-126, doi:10.1080/15622975.2018.1555377 (2020).

3 Hill, C. *et al.* Problem of diagnosis in postmortem brain studies of schizophrenia. *The American journal of psychiatry* **153**, 533-537 (1996).

4 Roberts, S. B. *et al.* Confirmation of the diagnosis of schizophrenia after death using DSM-IV: a Victorian experience. *Australian & New Zealand Journal of Psychiatry* **32**, 73-76 (1998).

5 Bell, C. C. DSM-IV: diagnostic and statistical manual of mental disorders. *Jama* **272**, 828-829 (1994).

6 Huang, G. M. C. Using CNS Tissue in Psychiatric Research. A Practical Guide. (1999).

7 Ferrer, I. *et al.* Brain protein preservation largely depends on the postmortem storage temperature: implications for study of proteins in human neurologic diseases and management of brain banks: a BrainNet Europe Study. *Journal of Neuropathology & Experimental Neurology* **66**, 35-46 (2007).

8 Kingsbury, A. E. *et al.* Tissue pH as an indicator of mRNA preservation in human post-mortem brain. *Molecular Brain Research* **28**, 311-318 (1995).

9 Stan, A. D. *et al.* Human postmortem tissue: what quality markers matter? *Brain research* **1123**, 1-11 (2006).

10 Verardi, V. & Croux, C. Robust regression in Stata. *The Stata Journal* **9**, 439-453 (2009).

11 ROBREG10: Stata module providing robust regression estimators v. S457114 (Boston College Department of Economics, 2010).

12 ROBSTAT: Stata module to compute robust univariate statistics v. S458524 (Boston College Department of Economics, 2018).

13 Wu, Y., Li, X., Liu, J., Luo, X.-J. & Yao, Y.-G. SZDB2.0: an updated comprehensive resource for schizophrenia research. *Human Genetics* **139**, 1285-1297, doi:10.1007/s00439-020-02171-1 (2020).

14 McAllum, E. J. *et al.* Regional iron distribution and soluble ferroprotein profiles in the healthy human brain. *Progress in Neurobiology* **186**, 101744, doi:https://doi.org/10.1016/j.pneurobio.2019.101744 (2020).

15 Pardiñas, A. F. *et al.* Common schizophrenia alleles are enriched in mutation-intolerant genes and in regions under strong background selection. *Nature Genetics* **50**, 381-389, doi:10.1038/s41588-018-0059-2 (2018).

16 Zhu, Z. *et al.* Integration of summary data from GWAS and eQTL studies predicts complex trait gene targets. *Nat Genet* **48**, 481-487, doi:10.1038/ng.3538 (2016).

17 Jaffe, A. E. *et al.* Mapping DNA methylation across development, genotype and schizophrenia in the human frontal cortex. *Nature Neuroscience* **19**, 40-47, doi:10.1038/nn.4181 (2016).

18 Fromer, M. *et al.* Gene expression elucidates functional impact of polygenic risk for schizophrenia. *Nature Neuroscience* **19**, 1442-1453, doi:10.1038/nn.4399 (2016).

`

19 Atkins, M., Burgess, A., Bottomley, C. & Riccio, M. Chlorpromazine equivalents: a consensus of opinion for both clinical and research applications. *Psychiatric Bulletin* **21**, 224-226 (1997).
20 Woods, S. W. Chlorpromazine equivalent doses for the newer atypical antipsychotics. *The Journal of clinical psychiatry* **64**, 663-667 (2003).
21 Brys, G., Hubert, M. & Struyf, A. Goodness-of-fit tests based on a robust measure of skewness. *Computational statistics* **23**, 429-442 (2008).
22 Bera, A. K. & Jarque, C. M. Efficient tests for normality, homoscedasticity and serial independence of regression residuals: Monte Carlo evidence. *Economics letters* **7**, 313-318 (1981).
23 Moors, J., Wagemakers, R. T. A., Coenen, V., Heuts, R. & Janssens, M. Characterizing systems of distributions by quantile measures. *Statistica Neerlandica* **50**, 417-430 (1996).
24 ROBREG: Stata module providing robust regression estimators v. S457114 (Boston College Department of Economics, 2010).
25 KMATCH: Stata module module for multivariate-distance and propensity-score matching, including entropy balancing, inverse probability weighting, (coarsened) exact matching, and regression adjustment v. S458346 (Boston College Department of Economics, 2017).
26 Mancias, J. D., Wang, X., Gygi, S. P., Harper, J. W. & Kimmelman, A. C. Quantitative proteomics identifies NCOA4 as the cargo receptor mediating ferritinophagy. *Nature* **509**, 105-109, doi:10.1038/nature13148 (2014).
27 Kuno, S., Fujita, H., Tanaka, Y.-k., Ogra, Y. & Iwai, K. Iron-induced NCOA4 condensation regulates ferritin fate and iron homeostasis. *EMBO reports* **23**, e54278, doi:https://doi.org/10.15252/embr.202154278 (2022).
28 Wang, Z. & Zhang, H. NCOA4: More than a receptor for ferritinophagy. *Journal of Cell Biology* **221**, doi:10.1083/jcb.202209004 (2022).
29 Boyle, A. P. *et al.* Annotation of functional variation in personal genomes using RegulomeDB. *Genome research* **22**, 1790-1797 (2012).
30 Huang, Y.-F., Gulko, B. & Siepel, A. Fast, scalable prediction of deleterious noncoding variants from functional and population genomic data. *Nature Genetics* **49**, 618-624, doi:10.1038/ng.3810 (2017).
31 Biological insights from 108 schizophrenia-associated genetic loci. *Nature* **511**, 421-427, doi:10.1038/nature13595 (2014).
32 Qi, T. *et al.* Identifying gene targets for brain-related traits using transcriptomic and methylomic data from blood. *Nature Communications* **9**, doi:10.1038/s41467-018-04558-1 (2018).

`
